# Doubling of the known set of RNA viruses by metagenomic analysis of an aquatic virome

**DOI:** 10.1038/s41564-020-0755-4

**Published:** 2020-07-20

**Authors:** Yuri I. Wolf, Sukrit Silas, Yongjie Wang, Shuang Wu, Michael Bocek, Darius Kazlauskas, Mart Krupovic, Andrew Fire, Valerian V. Dolja, Eugene V. Koonin

**Affiliations:** 1 National Center for Biotechnology Information, National Library of Medicine, National Institutes of Health, Bethesda, MD USA; 2 Department of Cellular and Molecular Pharmacology, University of California San Francisco, San Francisco, CA USA; 3 College of Food Science and Technology, Shanghai Ocean University, Shanghai, China; 4 Laboratory for Marine Biology and Biotechnology, Qingdao National Laboratory for Marine Science and Technology, Qingdao, China; 5 Institute of Biotechnology, Life Sciences Center, Vilnius University, Vilnius, Lithuania; 6 Archaeal Virology Unit, Institut Pasteur, Paris, France; 7 Department of Pathology, Stanford University School of Medicine, Stanford, CA USA; 8 Department of Genetics, Stanford University School of Medicine, Stanford, CA USA; 9 Department of Botany and Plant Pathology, Oregon State University, Corvallis, OR USA

**Keywords:** Water microbiology, Computational biology and bioinformatics, Metagenomics, Microbial ecology, Phylogeny

## Abstract

RNA viruses in aquatic environments remain poorly studied. Here, we analysed the RNA virome from approximately 10 l water from Yangshan Deep-Water Harbour near the Yangtze River estuary in China and identified more than 4,500 distinct RNA viruses, doubling the previously known set of viruses. Phylogenomic analysis identified several major lineages, roughly, at the taxonomic ranks of class, order and family. The 719-member-strong Yangshan virus assemblage is the sister clade to the expansive class *Alsuviricetes* and consists of viruses with simple genomes that typically encode only RNA-dependent RNA polymerase (RdRP), capping enzyme and capsid protein. Several clades within the Yangshan assemblage independently evolved domain permutation in the RdRP. Another previously unknown clade shares ancestry with *Potyviridae*, the largest known plant virus family. The ‘Aquatic picorna-like viruses/*Marnaviridae*’ clade was greatly expanded, with more than 800 added viruses. Several RdRP-linked protein domains not previously detected in any RNA viruses were identified, such as the small ubiquitin-like modifier (SUMO) domain, phospholipase A2 and PrsW-family protease domain. Multiple viruses utilize alternative genetic codes implying protist (especially ciliate) hosts. The results reveal a vast RNA virome that includes many previously unknown groups. However, phylogenetic analysis of the RdRPs supports the previously established five-branch structure of the RNA virus evolutionary tree, with no additional phyla.

## Abstract (web summary)

Metagenomic analysis of a single RNA virome from the Yangshan Deep-Water Harbour in China enabled the recovery of more than 4,500 distinct RNA viruses, doubling the known set of RNA viruses to date, and provided insights into their biology.

## Main

Metagenomics and metaviromics (that is, sequencing of DNA or RNA from virus particle fractions isolated from diverse environments or organisms) have led to rapid progress in virus discovery^1–9^. The International Committee on Taxonomy of Viruses has approved formal classification of viruses characterized solely by metagenomics^10^. The rapid advances in metaviromics have substantially expanded the known diversity of RNA viruses, yielding vast amounts of sequences for comprehensive studies on RNA virus evolution^11–17^.

Metagenomic investigation of various aquatic environments provides access to viromes of diverse prokaryotes and unicellular eukaryotes that could harbour ancient lineages of the RNA viruses^2^. Rich RNA viromes have been described in aquatic environments as diverse as Antarctic seas and wastewater^11,14,15,18–20^. Although metaviromic analyses do not typically identify the virus hosts, some of the marine RNA virome components have been phylogenetically anchored through similarity to viruses with known hosts. Perhaps the best characterized group of such viruses is the family *Marnaviridae*, which combines picorna-like viruses of diatoms and other stramenopiles^21–26^ with a growing number of species defined by metagenomics as probably infecting related aquatic unicellular eukaryotes^27,28^ (hereafter referred to as ‘protists’).

Another key development has been meta-transcriptome sequencing of invertebrate holobionts, doubling the size of the known RNA virome^29–33^. The high diversity of the invertebrate RNA virome suggests that RNA viruses of land plants and vertebrates evolved from viruses infecting invertebrates^2^. The known RNA viromes of plants, fungi, protists and bacteria have also expanded through meta-transcriptome sequencing, albeit not as massively as the invertebrate virome^19,34–41^.

A comprehensive phylogenetic analysis of RdRP, the only universally conserved protein of RNA viruses, produced a phylogenetic tree comprised of five major branches^42^. The deepest branch 1 includes the only known group of positive-sense (+)RNA viruses of prokaryotes, the leviviruses and their eukaryote-infecting descendants (narna- and ourmia-like viruses). The remaining four branches consist mostly of RNA viruses that infect eukaryotes. Branch 2 includes the assemblage of +RNA viruses denoted ‘picornavirus-like supergroup’, along with some of the smallest +RNA viruses in the *Solemoviridae* family and the largest +RNA viruses of the order *Nidovirales*. Branch 2 also contains two families of double-stranded RNA (dsRNA) viruses, *Partitiviridae* and *Picobirnaviridae*. Branch 3 consists solely of +RNA viruses, including the ‘Alphavirus supergroup’, a variety of viruses with small genomes resembling tombusviruses and nodaviruses, and the ‘Flavivirus supergroup’. Branch 4 consists of diverse dsRNA viruses, including the large families *Reoviridae* and *Totiviridae*, and the only known family of prokaryotic dsRNA viruses, *Cystoviridae*. Finally, branch 5 includes all known negative-sense (−)RNA viruses. A comprehensive virus ‘megataxonomy’ has been recently proposed and subsequently formally approved by the International Committee on Taxonomy of Viruses, in which the five major branches of the RdRPs correspond to five phyla in the kingdom *Orthornavirae*^43,44^. Despite these advances, a pressing question remains: would the current view of the RNA virome change substantially with deeper sampling, or are we getting close to an effectively complete coarse-grain picture of the global RNA virome? Is it likely that additional phyla of RNA viruses remain to be discovered?

**Fig. 1:**
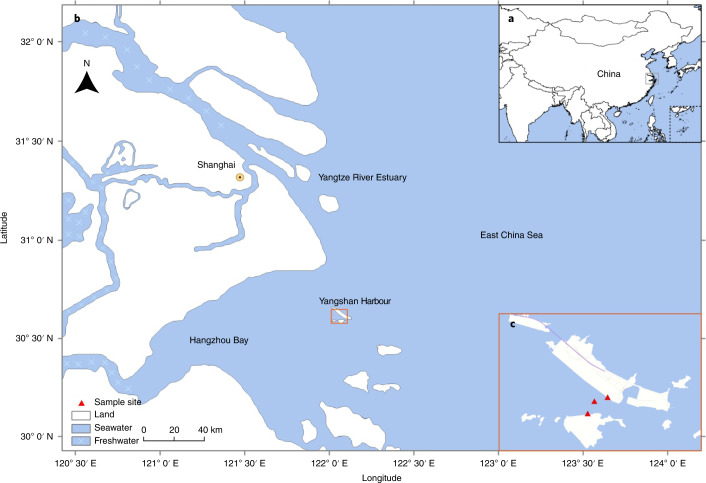
The Yangshan Deep-Water Harbour. **a**–**c**, Map of the Yangshan Deep-Water Harbour at three scales. **a**, Location within China. **b**, Magnified view of the region bounded by the grey box in **a**. **c**, Expanded view of the region marked by the orange box in **b**, showing the Yangshan Deep-Water Harbour. Red triangles in **c** mark seawater sampling sites. Map data: Google, 2014; Mapabc.com, 2014; CNES/Astrium, 2014 TerraMetics.

Here we report an extensive analysis of an RNA virome in water samples from Yangshan Deep-Water Harbour near Shanghai, China, where the Yangtze River meets the East China Sea (Fig. 1). This analysis of the RNA virome from a single, albeit complex, aquatic habitat doubles the known diversity of RNA viruses, identifying several previously unrecognized groups of +RNA viruses (roughly, at the class, order or family taxonomic ranks). Despite the discovery of numerous virus groups, phylogenetic analysis of the RdRPs shows that a substantial majority of the identified viruses belong to already established phyla of RNA viruses^44^.

## Results

### Diversity of RNA viruses in the Yangshan harbour virome

RNA virome analysis performed using complementary DNA derived from approximately 10 l of samples from Yangshan Deep-Water Harbour yielded 4,593 nearly full-length RNA virus RdRPs that formed 2,192 clusters at 75% amino acid identity which represents virus diversity at a level between species and genus. Among the RdRP sequences from GenBank (October 2018), 2,021 comparable clusters were detected. Thus, the 10 l water sample analysed here more than doubles the known diversity of RNA viruses.

**Fig. 2:**
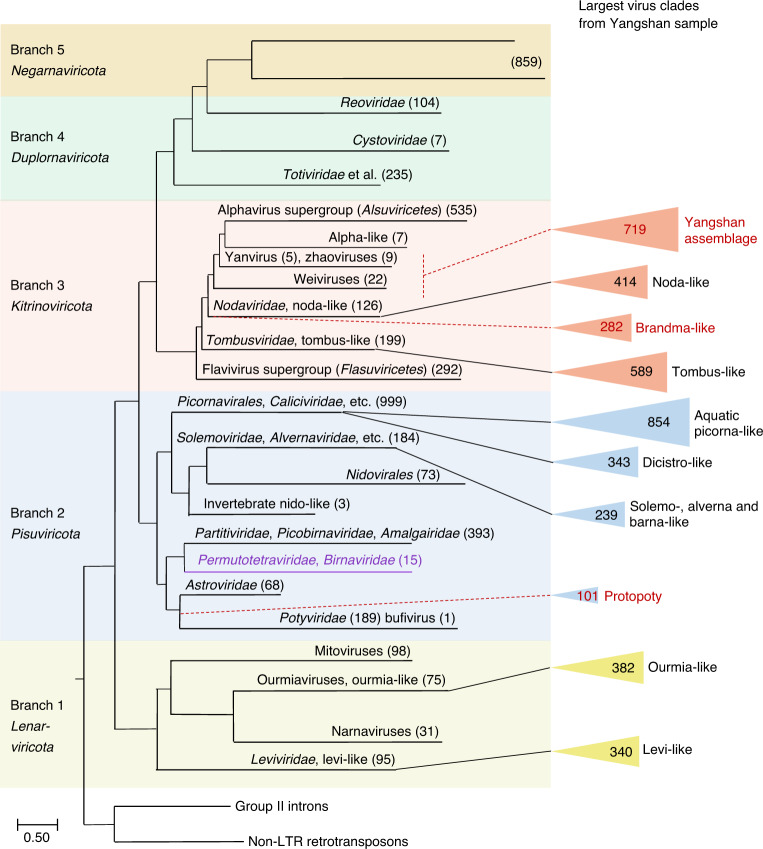
Schematic phylogenetic tree of the RNA virus RdRPs. The reverse transcriptases of group II introns and non-long-terminal-repeat (non-LTR) transposons were used as an outgroup to root the tree. The overall tree topology encompassing five major RdRP branches (highlighted by different background colours) has been described previously^42^. These branches correspond to RNA virus phyla, which are shown under the branch numbers. The positions of the largest clades of viruses identified in this study are indicated and represented by triangles, the areas of which are roughly proportional to the number of viruses in each clade (shown inside the triangle). The numbers in parentheses correspond to previously identified viruses included in the analysis in ref. ^42^. Provisional names of the previously undescribed virus clades are shown in red. Purple text denotes a virus lineage with permuted RdRPs (*Permutotertraviridae* and *Birnaviradae*) which was not included in the previous study^42^.

Phylogenetic analysis assigned 85% of the RdRPs from the Yangshan RNA virome to 9 clades and one complex assemblage, each comprising more than 100 RdRps from several clusters (Fig. 2 and Supplementary Dataset 1). Seven of these clades blended into those defined previously, whereas two previously unknown clades and the assemblage were dominated by viruses from the Yangshan virome (Fig. 2). All these clades represented +RNA viruses of the phyla *Lenarviricota*, *Pisuviricota* and *Kitrinoviricota*, whereas no members of *Negarnaviricota* were found. Only six dsRNA viruses (*Duplornaviricota*) were identified, but were not further analysed. No enveloped +RNA viruses of the families *Flaviviridae* and *Togaviridae* were detected. Common +RNA viruses of terrestrial vertebrates and plants (for example, members of *Picornaviridae, Caliciviridae, Virgaviridae* or *Potyviridae*) were also absent from the Yangshan virome.

The largest RdRP group in the Yangshan virome (854 members; Supplementary Datasets 1 and 2) belongs to the ‘Aquatic picorna-like’ clade (order *Picornavirales*)^30,42^ in the phylum *Pisuviricota* (Fig. 2). This clade contains the *Marnaviridae* and other protist-infecting viruses as well as viruses identified in holobionts of molluscs, annelids and other marine invertebrates whose diets include protists. The largest of the 323 broad RdRP clusters in the Yangshan virome—OV.1 (where OV indicates Ocean Viruses, an operational term for RdRP clusters), with 653 members (Supplementary Datasets 1 and 2)—fell entirely into the *Marnaviridae*, vastly expanding the diversity of this family and highlighting the need for a taxonomic upgrade^28^. Given that isolated *Marnaviridae* infect diatoms and other aquatic Stramenopile protists^21,22,26,39,45^, most OV.1 members are likely to infect related unicellular eukaryotes. The genome organizations of the previously recognized marnaviruses and those from the Yangshan virome are nearly uniform: they encode either one or two polyproteins encompassing the same set of protein domains (Supplementary Dataset 2).

*Pisuviricota* accommodated another large clade with 343 RdRPs related to those of *Dicistroviridae* (order *Picornavirales*; Fig. 2), which infect marine and terrestrial arthropods^30,46^. Although these and previously recognized dicistroviruses share the same genome organization, they form sister groups in the RdRP phylogeny (for example, OV.9 and OV.13 in Supplementary Datasets 1 and 2), suggestive of distinct host ranges. The third largest clade within *Pisuriviricota* (239 RdRPs, including OV.12 and OV.27) joined the lineage that includes plant *Solemoviridae*, fungal *Barnaviridae* and protist *Alvernaviridae*.

The fourth clade (101 members) of the Yangshan virome RdRPs within *Pisuviricota* (including OV.16 and OV.23; Supplementary Datasets 1 and 2) is a sister group to *Potyviridae*, the largest family of plant viruses^47,48^. Because the marine virome appears to be ancestral to the terrestrial plant virome^49^, these aquatic relatives of the potyviruses probably resemble the common ancestor, and were accordingly dubbed Protopotyviruses (Fig. 2). Protopotyviruses share with potyviruses the conserved tandem of a chymotrypsin-like protease and the RdRP, but lack the SF2 helicase and the papain-like protease characteristic of potyviruses (Supplementary Dataset 2). Given the evolutionary affinity between the potyvirus SF2 helicase and the homologous helicase of flavi-like viruses^50^, this is likely to be a late acquisition in potyviruses. Most of the protopotyvirus genomes encode a single-jelly-roll capsid protein (SJR-CP), likely inherited from the common ancestor of all eukaryotic RNA viruses^42^. In contrast, filamentous potyviruses encode a distinct capsid protein^51^, which is homologous to nucleocapsid proteins of (−)RNA viruses^52,53^. These findings are consistent with the ancestral status of protopotyviruses with respect to potyviruses.

More than 1,700 Yangshan virome RdRPs belong to *Kitrinoviricota*; this was unexpected, given that so far, *Kitrinoviricota* consisted largely of viruses of terrestrial plants and animals^30,42,54^. Two virus groups from the Yangshan virome fell within the Tombus-like (589 members) and Noda-like (414 members) clades of *Kitrinoviricota* (Fig. 2). *Nodaviridae* is not monophyletic with respect to the Yangshan nodavirus-like group: the nematode-infecting Orsay-like viruses^55^ as well as *Sclerophthora macrospora virus A*^56^ and *Plasmopara halstedii virus A*^57^, both of which infect oomycetes, are nested within the diversity of the Yangshan RdRPs (OV.3 in Supplementary Dataset 2). Oomycetes, particularly those that parasitize diatoms^58^, are the plausible hosts for the noda-like viruses in the Yangshan virome, although free-living marine nematodes could not be ruled out as hosts^59^. Unlike the known members of *Nodaviridae*, most of the noda-like viruses identified in the Yangshan virome have monopartite genomes, which appears compatible with an ancestral state. None of the major Yangshan virome clades among *Kitrinoviricota* joined the ‘Alphavirus supergroup’ (class *Alsuviricetes*) comprising viruses that infect mostly plants, as well as animals and fungi.

**Fig. 3:**
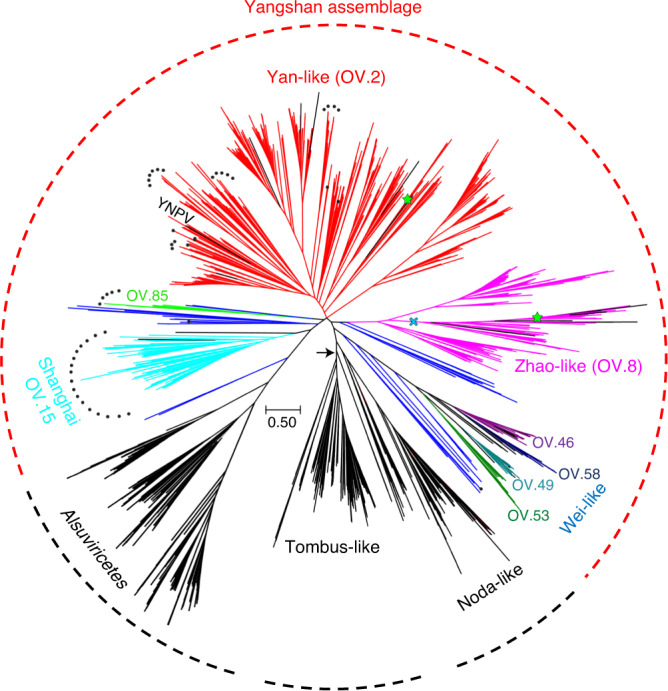
The Yangshan virus assemblage. The rootless tree represents the subtree of branch 3 (*Kitrinoviricota*) in which the Yangshan virus assemblage belongs. The positions of RdRPs of previously reported viruses are shown in black. Dark blue represents assorted small clusters of the Yangshan assemblage. Other colours represent clusters (as indicated) of which Yan-like (red), Zhao-like (magenta) and Shanghai (electric blue) correspond to eponymous major clades, whereas the Wei-like clade encompasses four clusters. The permuted RdRPs are marked by black dots and RdRP groups with non-standard genetic codes are marked with green stars. The blue cross marks a virus group within the Zhao-like clade that includes viruses using protist genetic codes and encoding a capping enzyme similar to that of nodaviruses. The arrow points to a root position as in the tree in Fig. 2. YNPV, Yellowstone National Park virus^60^.

A previously unknown, highly diverse assortment of RdRPs (719 members; hereafter, the Yangshan assemblage) consists of several clades positioned between the noda-like viruses and *Alsuviricetes* within *Kitrinoviricota* (Figs. 2 and 3). This assemblage includes three previously described small groups of viruses—namely, Weiviruses, Yanviruses and Zhaoviruses—and several unclassified viruses. The largest clade within the Yangshan assemblage (OV.2, hereafter the Yan-like clade) consists of 431 Yangshan RdRPs, all 5 previously described Yanviruses^30^ and several uncharacterized viruses, including the solitary RNA virus isolated from an acidic hot spring in Yellowstone National Park dominated by archaea^60^ (Fig. 3).

**Extended Data Fig. 1:**
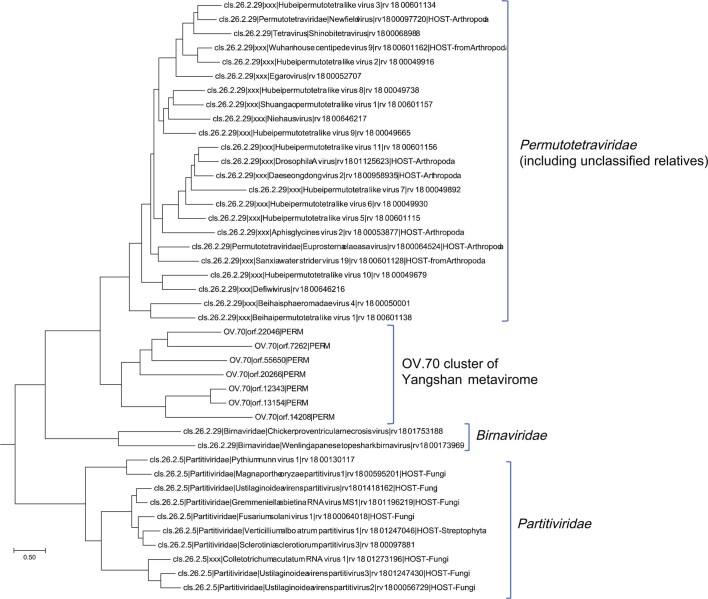
Phylogenetic tree of the permuted RdRps of *Permutotetraviridae, Birnaviridae* and a related OV.70 cluster of seven RdRps identified in the Yangshan virome. Note that this lineage of permuted RdRPs is confidently lodged as additional clade in Branch 2 (*Pisuviricota*) as a sister to *Partitiviridae* lineage.

The Yan clade is a hotspot of RdRP domain permutation that apparently occurred on ten independent occasions within this clade alone. Previously, such permutations had been detected in the *Permutotertraviridae* and *Birnaviridae*^61–63^, but were excluded from our previous analysis due to interference with multiple RdRP alignments caused by the permutation^42^. Here we developed a procedure for swapping domains in the permuted RdRPs to restore the original domain order and included these reconstructed RdRP sequences in the phylogenetic analysis (Fig. 2). In the resulting trees, permutotetraviruses and birnaviruses formed a well-supported clade within *Pisuviricota* that was far removed from the Yangshan assemblage (Extended Data Fig. 1), again pointing to convergent evolution of this trait in diverse viruses.

**Extended Data Fig. 2:**
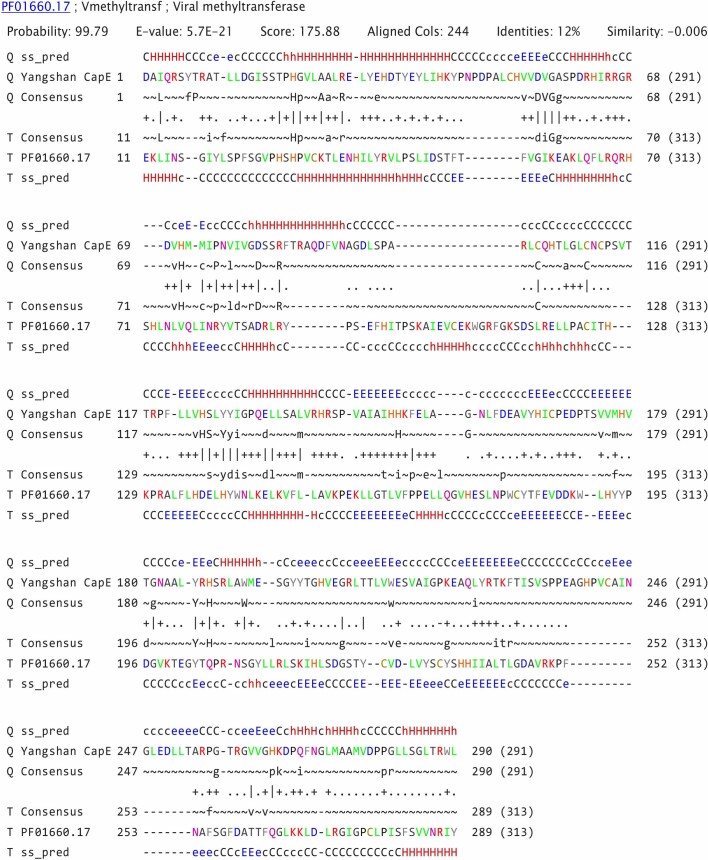
Results of the HHpred search seeded with the putative capping enzyme of Yan-like viruses. H(h), α-helix; E(e), β-strand; C(c), coil. The single-letter designations of the amino acid residues are coloured according to their physical properties.

**Extended Data Fig. 3:**
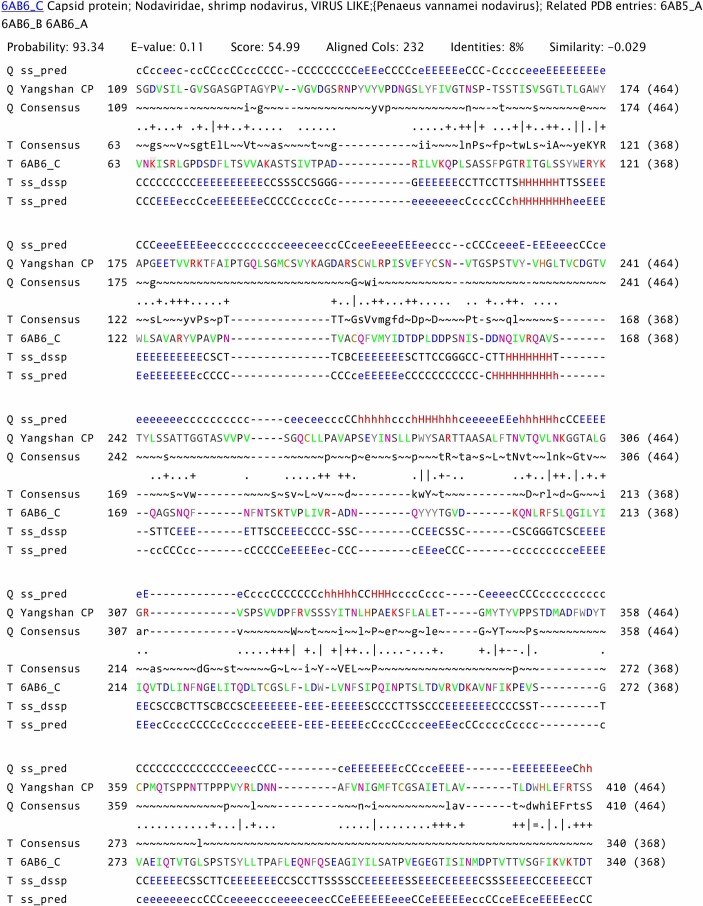
Results of the HHpred search seeded with the putative capsid protein of Yan-like viruses. H(h), α-helix; E(e), β-strand; C(c), coil. The single-letter designations of the amino acid residues are colored according to their physical properties.

Of the 387 long Yan-like contigs, 220 encode a distinct SJR-CP and 100 encode a capping enzyme. A HHpred comparison of the profile created from the sequence alignment of Yan-like virus capping enzymes against the profile database exclusively retrieved the capping enzymes of *Alsuviricetes* (PF01660.17; Vmethyltransf; *P* = 99.8; Extended Data Fig. 2), in support of the placement of the Yan-like clade near the base of *Alsuviricetes* (Fig. 3). The profile–profile comparisons also showed that the SJR-CP protein of Yan-like viruses has a two-domain organization, including the shell and projection domains, similar to the capsid proteins of certain nodaviruses and tombusviruses (Extended Data Fig. 3), solidifying the position of the Yan-like clade in the tree.

**Fig. 4:**
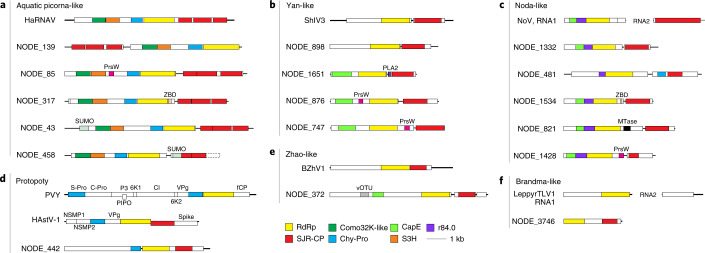
Diversity of domain organizations in the major clades of marine RNA viruses in the Yangshan virome. **a**, Aquatic picorna-like virus clade (as in Fig. 2). **b**, Yan-like virus clade (as in Fig. 3). **c**, Noda-like virus clade (as in Figs. 2 and 3). **d**, Protopotyvirus clade (as in Fig. 2). **e**, Zhao-like virus clade (as in Fig. 3). **f**, Brandma-like virus clade (as in Fig. 2). Each panel contains a genome map(s) of a phylogenetically close reference virus(es) at the top and viruses discovered in this study, identified as ‘NODE_NN’. NODE numbers correspond to contig gene IDs listed in Supplementary Datasets 3 and 5. Functional domains are colour coded and the colour key for the recurrent domains is shown at the bottom of the figure. HaRNAV, Heterosigma akashiwo RNA virus; PVY, potato virus Y; HAstV-1, human astrovirus 1; ShIV3, Shahe isopoda virus 3; BZhV1, Beihai zhaovirus-like virus 1; NoV, Nodamura virus; LeppyrTLV1, Leptomonas pyrrhocoris tombus-like virus 1; PrsW, PrsW-family protease; ZBD, zinc-binding domain; PLA2, phospholipase A2; vOTU, viral ovarian tumour protease; MTase, methyltransferase; Chy-Pro, chymotrypsin-like protease; S-Pro, serine protease; C-Pro, cysteine protease; VPg, viral genome-linked protein; CP, capsid protein; fCP, filamentous capsid protein; NSMP, non-structural mature protein; CapE, capping enzyme; S3H, superfamily 3 helicase; Como32K-like, comovirus 32K-like protease; r84.0, functionally uncharacterized domain conserved in RNA viruses; P3, protein 3; 6K1 and 6K2, 6 kDa proteins 1 and 2; PIPO, pretty interesting *Potyviridae* open reading frame protein; Spike, spike protein; CI, cylindrical inclusion protein.

Another major clade within the Yangshan assemblage (OV.8, hereafter the Zhao-like clade) consists of 113 members (Fig. 3; Supplementary Datasets 1 and 2) and includes a previously orphan cluster of 9 Zhaoviruses identified in marine invertebrates^30^ along with ‘ciliovirus’ and ‘brinovirus’ from a San Francisco wastewater virome^64^. The Zhaoviruses, ‘ciliovirus’ and ‘brinovirus’, together with 36 Yangshan virome viruses, form a separate group within the Zhao-like clade. This group is distinguished by using ciliate and other protist genetic codes (see below) and by encoding a capping enzyme similar to the distinct capping enzyme of nodaviruses (Fig. 4 and Supplementary Dataset 2).

The third major clade in the Yangshan assemblage, denoted ‘Shanghai’, harbours 74 Yangshan RdRPs and the unclassified ‘eunivirus’ (KF412900), which was identified in a wastewater virome (Fig. 3; OV.15). The signature of this clade is the domain permutation of the RdRP that apparently occurred at the base of this clade. Finally, the Wei-like clade with 57 Yangshan RdRPs (clusters OV.46, OV.49, OV.53, OV.58, OV.192, OV.233, OV.250 and OV.262) also includes 15 Weiviruses^30^. The phylogenomic diversity within the Yangshan assemblage seems to justify the establishment of a virus class, subdivided into multiple orders and families.

The last large clade within *Kitrinoviricota* (‘Brandma-like’ viruses) combines 282 RdRPs (cluster OV.4; Supplementary Datasets 1 and 2) with several previously reported orphan viruses from diverse sources (Fig. 2 and Supplementary Dataset 2). Most Brandma-like viruses have small, 4–5-kb genomes that encode only two recognizable domains, RdRp and SJR-CP (Supplementary Dataset 2). The Brandma-like viruses form a sister group to the Noda-like viruses (Fig. 2).

**Extended Data Fig. 4:**
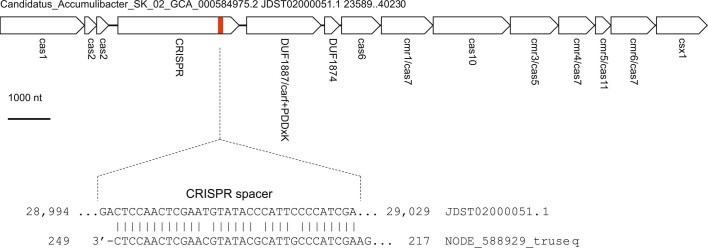
A nucleotide sequence match between a Yangshan RNA virome contig bearing a levi-like RdRP (bottom line) and the type III-B CRISPR spacer locus of the bacterium *Candidatus Accumulibacter sp. SK-02*.

Finally, two large clusters of the Yangshan RdRP belong to *Lenarviricota*, grouping with the +RNA bacteriophages of the *Leviviridae* and levi-like viruses (340 members), or with ourmia-like viruses (382 members), the eukaryote-infecting descendants of +RNA bacteriophages^2,30,37^ (Fig. 2 and Supplementary Datasets 1 and 2). Such strong representation of the levi-like phages and ourmia-like viruses in an aquatic RNA virome is expected, and so is the absence of the other clades of *Lenarviricota*, namely, mito- and narnaviruses, common capsid-less +RNA agents of fungi^65^. Our search for RNA virus sequences homologous to bacterial clustered regularly interspaced short palindromic repeats (CRISPR) spacers yielded a single match between one of the Yangshan RNA virome contigs bearing a levi-like RdRP and the reverse transcriptase-associated type III-B CRISPR locus of the bacterium *Candidatus* Accumulibacter sp. SK-02 (Extended Data Fig. 4). To our knowledge, CRISPR spacers matching RNA virus genomes have not been reported previously. Although caution is warranted in the interpretation of this solitary RNA virus protospacer, this finding suggests that CRISPR–CRISPR-associated protein (Cas) systems can target RNA viruses^66^.

Overall, each of the three phyla of +RNA viruses^42,44^ is well represented in the complex Yangshan virome (Fig. 2). Among the largest (more than 100 members) clusters of the discovered RdRPs, four (Yan-like, Zhao-like, Brandma-like and Protopoty) form distinct clades within *Pisuviricota* and *Kitrinoviricota* (Figs. 2 and 3), each assimilating a handful of previously identified viruses of uncertain evolutionary provenance that now find their ‘phylogenetic homes’.

In addition to the RdRPs that could be assigned to previously identified clades at different depths of the phylogenetic tree, we attempted to detect putative highly divergent RdRPs using complementary approaches (see Methods) and identified 13 singleton RdRP sequences. The further expansion of the global RNA virome is expected to allow more confident assignment of these divergent RdRPs to additional clades, as was the case with the Yan-like, Zhao-like, Brandma-like and Protopoty clades.

### Distinct domain architectures of virus proteins in the Yangshan virome

**Extended Data Fig. 5:**
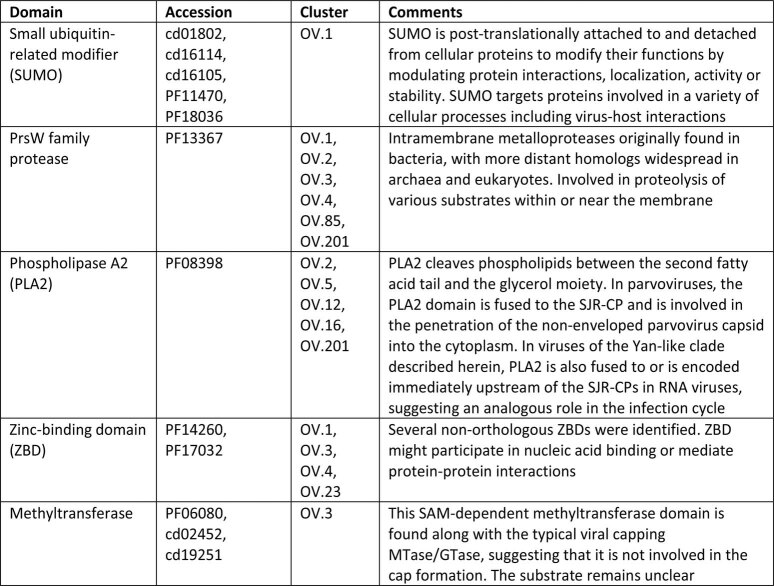
Protein domains identified in the Yangshan RNA virome that were not previously observed in known RNA viruses. In the virome contigs, the nucleotide sequences encoding these domains were linked to those encoding RdRP thus demonstrating that they belonged to RNA virus genomes.

Analysis of the domain content of the longer Yangshan contigs indicates that the genome organizations are typically similar within clusters enriched in Yangshan viruses and closely resemble the genome organizations of the previously known viruses from the same clades. Nevertheless, we identified several domains that have not been previously observed in any RNA viruses (Fig. 4 and Supplementary Dataset 2), including small ubiquitin-like modifier (SUMO), PrsW-like protease and phospholipase A2 (Extended Data Fig. 5). In addition, many Yangshan virome clusters included viruses that appear to have relatively recently acquired other domains, in particular, Zn^2+^-binding and methyltransferase domains, as well as conserved domains of unknown function. Collectively, these observations reveal dynamic acquisition of multiple functional domains that might be involved in distinct virus–host interactions.

### Alternative genetic codes in RNA viruses

The RdRPs in the Yangshan virome were identified in end-to-end six-frame translations of the contigs. Mapping the RdRP core domain profile to the best-matching frame established the RdRP core boundaries for each contig. In 98.7% of the contigs, the RdRP core translations obtained with the standard genetic code contained no stop codons. The remaining RdRP-coding regions, however, contained up to 26 stop codons (Supplementary Dataset 3), suggesting alternative genetic codes. These contigs were translated using all 26 known variants of the genetic code, and the code that yielded the longest protein including the RdRP core was selected for each contig. Viruses using alternative codes were identified in the Yan-like and Zhao-like clades within the Yangshan assemblage where the use of alternative codes is mostly confined to two distinct, smaller lineages (Fig. 3). Outside the Yangshan assemblage, alternative genetic codes were detected among the Ourmia-like viruses in *Lenarviricota*, Aquatic picorna-like and Dicistro-like viruses in *Pisuviricota* and Tombus-like and Noda-like viruses in *Kitrinoviricota* (Supplementary Dataset 3). The viruses with alternative codes probably infect protists and particularly, ciliates.

## Discussion

Analysis of metaviromic samples from the single, mixed marine and freshwater habitat described here roughly doubles the known diversity of RNA viruses—as defined by an RdRP-sequence similarity threshold that falls between the species and genus ranks^42^. This discovery reveals the richness of complex aquatic environments and calls for in-depth study of similar biomes and viromes.

Most of the previously unknown viruses join the major lineages of RNA viruses, now established as phyla of the kingdom *Orthornavirae*^42,44^. Nevertheless, several major taxa are expected to emerge from this analysis, probably in the ranks of class (Yangshan assembly), order (for example, Picorna-like aquatic and Protopoty clades or Yan-like, Zhao-like, Wei-like and Shanghai viruses in the Yangshan assembly) and family (such as the Zhao-like subclade highlighted in Fig. 3).

We show that diversity is the defining factor for obtaining a reliable phylogeny of RNA viruses; once virus groups fill up with multiple, diverse RdRP sequences, most sequences that originally appeared as orphans coalesce into distinct clades and move up the tree. This trend is exemplified by several clades in the Yangshan assemblage (Fig. 3).

Our findings expand the understanding of the structural, functional and evolutionary plasticity of the +RNA viruses. We identified multiple virus lineages with RdRP domain permutation that is far more common than previously appreciated and is a recurrent variation in RdRP evolution rather than an ancestral configuration as has been suggested^62^. Previously unknown cases of domain recruitment by +RNA viruses were detected, suggesting unsuspected facets of virus–host interactions.

The Yangshan RNA virome analysis clarifies some critical stages in the evolution of +RNA viruses. Thus, the viruses of the Yangshan assemblage are probably evolutionary intermediates between simple, tombus-like viruses at the base of *Kitrinoviricota* and the more complex viruses of the expansive class *Alsuviricetes*. Similarly, Protopotyviruses seem to be the missing evolutionary link between simple, ancestral *Pisuviricota* and the more complex potyviruses. Likewise, recently discovered ‘plastroviruses’ appear to be evolutionary intermediates between astro-like and poty-like viruses^67^. Further identification of such missing links is expected to yield detailed scenarios for the origin of major groups of RNA viruses.

**Extended Data Fig. 6:**
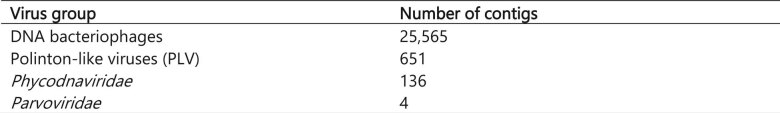
DNA virus sequences in the Yangshan virome.

Inference of virus host range is a weak link in metaviromics. In the case of the Yangshan virome, clues come from the assignment of the largest cluster of Yangshan viruses to the family *Marnaviridae*, which is so far thought to include only protist viruses, and from the alternative genetic codes in several virus groups in the Yangshan assemblage, which also points to protist hosts. Additionally, in an attempt to characterize the Yangshan virome more comprehensively, we searched the DNA fraction of the Yangshan virome for signature proteins of different groups of DNA viruses. The overwhelming majority of the identified contigs belonged to various DNA bacteriophages and protist viruses, providing further support of the host assignments of RNA viruses (Extended Data Fig. 6). Thus, multiple lines of indirect evidence indicate that a substantial fraction—probably the majority—of the viruses in the Yangshan extracellular aquatic RNA virome infect unicellular eukaryotes. In particular, it is possible that the virus genome obtained from a Yellowstone National Park hot spring, for which an archaeal host has been proposed^60^, actually belongs to a protist virus. Apart from protists, some viruses in the Yangshan virome, such as dicistro-like viruses, are likely to infect marine arthropods, whereas for levi-like viruses, bacterial hosts can be confidently inferred.

The Yangshan virome could also shed light on RNA virus ecology. Quantitative analysis of contig occurrence revealed several extremely abundant viruses that are likely to reflect virus blooms on the most abundant hosts (Extended Data Fig. 10; Supplementary Dataset 4). The ecological composition of the Yangshan biome could also be relevant to the dominance of non-enveloped +RNA viruses in the extracellular RNA virome, to the exclusion of (−)RNA viruses. According to RdRP phylogenetic tree, *Negarnaviricota* are nested within *Duplornaviricota*, which are themselves lodged within the +RNA virus radiation (Fig. 2), implying more recent origin of (−)RNA viruses. Given that the greatest diversity of *Negarnaviricota* is found in invertebrates^29^, it has been suggested that this virus phylum evolved during the explosive Cambrian diversification of invertebrates^2,49^. This scenario is supported by the near absence of (−)RNA viruses in protists. A similar logic applies to the absence of the enveloped viruses of the *Alsuviricetes* and *Flasuviricetes* in the Yangshan virome: none of these viruses are known to infect protists. However, we cannot rule out that some unidentified technical bias in the procedures employed in this work also contributed to the dominance of +RNA viruses in the Yangshan virome.

Thus, a virome from a single, complex aquatic habitat doubles the known diversity of RNA viruses, points to unexpected features of virus biology and evolution, and is bound to substantially expand the taxonomy of RNA viruses. Nevertheless, the recently developed megataxonomic structure of the global RNA virome that includes five phyla of the kingdom *Orthornavirae*^42,44^ withstood the challenge from this data and might be approaching stability.

## Methods

### Sampling site, water sample collection and preparation

**Extended Data Fig. 7:**
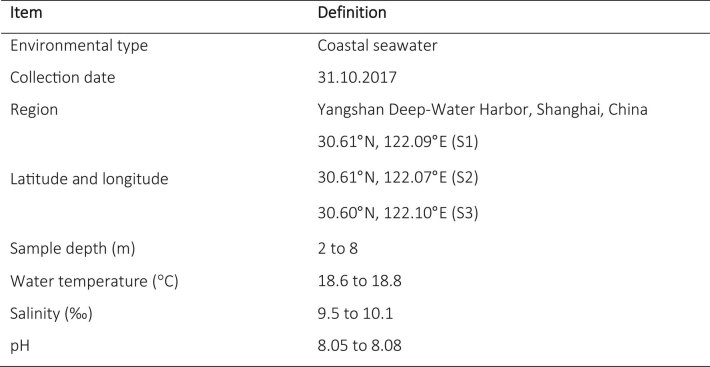
General characteristics of the oceanic RNA virome of Yangshan Deep Water Harbor.

**Extended Data Fig. 8:**
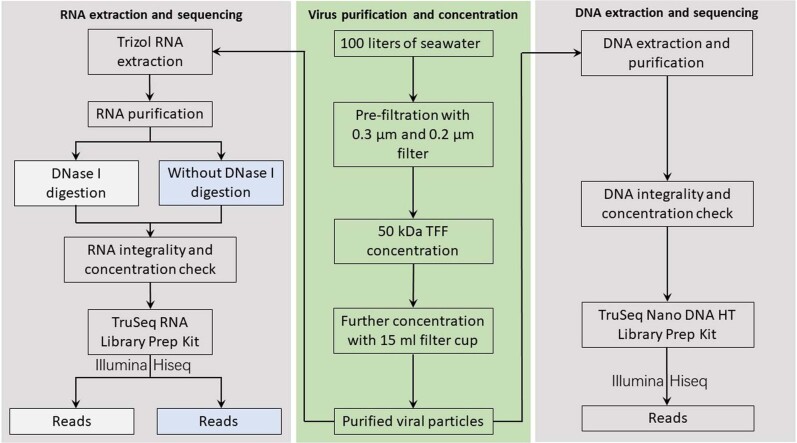
Schematic of purification and sequencing of the oceanic RNA and DNA viromes. TFF, tangential flow filtration.

One-hundred litres of seawater were collected from three distinct sites in Yangshan Deep-Water Harbour, Shanghai, China on October 31 2017 (Extended Data Fig. 7). The samples were collected at the depths of 2–8 m from 3 sites in the Yangshan Deep-Water Harbour (>40 m depth) located between the Yangtze River estuary and Hangzhou Bay of East China Sea (Fig. 1 and Extended Data Fig. 7). The salinity of the harbour water (approximately 10‰, varying depending on currents) was intermediate between that of Yangtze River (0.2‰) and East China Sea (approximately 30‰), potentially contributing to the complexity of this aquatic habitat, which probably harbours freshwater-, estuary- and seawater-specific organisms, with the potential presence of some benthic organisms. The water samples were initially settled at 4 °C for 12 h, and viruses were isolated using tangential-flow-filtration procedures as previously described^68^ (Extended Data Fig. 8). The concentrated viral particles were stored at −80 °C before use. The absence of bacterial or cellular contamination in the filtrate was confirmed by transmission electron microscopy.

### Virus nucleic acid extraction

One millilitre of concentrated virus (approximately 10^10^–10^11^ virus particles isolated from 10 l of seawater) was used for extraction of either DNA using Purification Resin and Mini Column (Promega)^69^, or RNA by using TRIzol LS Reagent (Invitrogen) and the Fast Total RNA Kit (Generay Biotech) (Extended Data Fig. 8). The integrity and concentration of nucleic acids were measured with NanoDrop 2000 (Thermo) and Qubit 3 analyser (Invitrogen). Virus RNA extracts (approximately 1.3 µg total) were subsequently divided into two parallel fractions. One was incubated with 1 μl DNase I (Thermo) at 37 °C for 10 min, and the other remained untreated.

### High-throughput DNA and RNA sequencing

**Extended Data Fig. 9:**
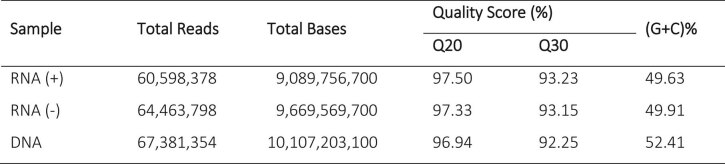
Sequencing data for each metaviromic cDNA library.

Two different RNA library-priming approaches (random-hexamer priming and template-switching reverse transcription) were used. Two 150 bp paired-end libraries (cDNA from total RNA) were generated using random-hexamer priming with the TruSeq RNA Library Prep Kit (Illumina) for the virus RNA extracts with or without DNase I digestion. Two single-end libraries were generated for the DNase I treated viral RNA extract using template-switching reverse transcription with the SMARTER stranded total RNA-seq kit (Clontech): one without fragmentation, and one with 4 min fragmentation at 94 °C, according to manufacturer’s instructions. The TruSeq Nano DNA HT Library Prep Kit (Illumina) was used to generate a 150-bp paired-end DNA library from the virus DNA extracts (Extended Data Fig. 8). High-throughput sequencing was performed on the Illumina MiSeq platform with v3 chemistry, and subsequently on the Illumina HiSeq 2500 platform. Both the library preparation and high-throughput sequencing were performed by Biozeron (Shanghai). Sequencing parameters are shown in Extended Data Fig. 9.

### Computational subtraction

Sequencing adapters were first removed, and nucleotides with quality scores lower than 20 were trimmed from the ends of reads using the cutadapt tool (https://cutadapt.readthedocs.io/en/stable/). To obtain a ‘clean’ RNA dataset, DNA-matching reads were computationally subtracted from the pool of RNA reads before virus genome assembly using a *k*-mer based approach. All unique 30-mers present in the DNA library were collected and RNA reads with an exact match to any 30-mer in the DNA library (on either read in the mate-pair for the paired-end datasets) were then excluded prior to contig assembly. Then, 20- and 25-mers were also tested to ensure that the subtraction was not sensitive to the *k*-mer length. As anticipated by a priori calculations, while subtraction using 20-mers resulted in gross overfiltering, 25- and 30-mers resulted in very similar numbers of removed reads. We also repeated the subtraction separately for the RNA libraries with or without DNase I treatment using 30-mers from the DNA dataset, and found no substantial difference in the numbers of removed reads (about 50% in each case), thereby underscoring the importance of in silico DNA subtraction.

### Contig assembly

Contigs from the paired-end random-priming library were assembled using SPADES v.3.11.1 in metagenomics mode, while contigs from the single-end template-switching library were assembled using SPADES v.3.7 in metagenomics mode (v.3.11.1 only supports assembly of paired-end reads in metagenomics mode). After assembly, the two sets of contigs were unified into a single set of non-redundant contigs by excluding any contig from the template-switching dataset that shared more than 90% of its 15-mer sub-sequences with any contig in the random-priming dataset.

### RdRP identification, clustering and phylogenetic analysis

RdRp sequences were identified using PSI-BLAST, which was run against the six-frame end-to-end translations of all contig sequences. Multiple alignments of virus RdRPs and reverse transcriptases from group II intron and non-long-terminal-repeat retrotransposons^42^ were used to generate query position-specific scoring matrices. Sequences that covered at least 75% of the query profile length were considered to contain full-length RdRP cores. This analysis identified almost 75,000 contigs (7.8% of all contigs; 150–11,000 nucleotides size range) encoding predicted proteins with significant amino acid sequence similarity to previously identified RdRP. Of these, 4,593 proteins were operationally considered ‘full-length’ RdRP. Initial clustering of the identified full-length RdRPs was performed using MMSEQ2^70^ with sequence similarity threshold of 0.5. When the same position-specific scoring matrices were employed to search the protein sequences from GenBank, 5,481 full-length, non-redundant (<90% identity) RdRP sequences were identified that formed 2,021 clusters. After the addition of 4,593 full-length sequences from the Yangshan dataset, the combined set of 10,074 sequences produced 4,213 clusters under the same clustering procedure, increasing the number of clusters by a factor of 2.08.

Multiple alignments of sequences within clusters were generated using MUSCLE^71^. Cluster-derived profiles were compared to existing profiles using the HHsearch program^72^ to broadly assign the Yangshan sequences to the five major branches of RdRPs^42^. Iterations of clustering using HHsearch and profile–profile alignments using HHalign were performed to refine the positions of the Yangshan sequences within the RdRP tree. The clusters were delineated such as to include sufficiently diverse sequences and to be significantly enriched with sequences from the metaviromic sample. This procedure yielded 323 clusters (OV.1 to OV.323 in Supplementary Dataset 1) containing from 1 to 653 sequences. Phylogenetic trees for the cluster alignments were generated using FastTree^73^ with the WAG evolutionary model and gamma-distributed site rates. Nearly monophyletic groups of Yangshan RdRPs (containing at least 90% of Yangshan metagenome sequences) or mixed, but shallow groups of Yangshan RdRPs (corresponding to the tree depth of less than 1.0 substitution per site) were considered to be distinct Yangshan clusters.

For further phylogenetic analysis, the full-length RdRPs of the Yangshan set were aligned with their previously identified homologues and subjected to additional clustering based on the resulting preliminary phylogenetic trees. The resulting clusters were then fitted into the previously constructed RdRP tree^47^ using a procedure that involved several iterations of aligning Yangshan RdRPs with those from GenBank, constructing preliminary trees, and extracting Yangshan RdRPs that grouped together. The overwhelming majority of the Yangshan sequences (4,348 of 4,593, or 95%) and all large clusters (31 clusters encompassing 22 or more sequences each) were affiliated with previously identified RdRP lineages (Fig. 2; Supplementary Dataset 1).

The RdRp permutations make permuted sequences unalignable with those of the canonical configuration. To incorporate them into the phylogenetic analysis, the following de-permutation procedure was performed: first, permuted sequence were identified, clustered using MMSEQ2 with sequence similarity threshold of 0.5 and aligned with each other. Profile–profile alignments between these clusters and their closest canonical configuration relatives were performed using the HHALIGN program; the boundaries of the permuted catalytic loop were determined by examining the alignment and the corresponding alignment fragment was transposed to the canonical location (typically the location of the gap against the canonically located loop). Then the de-permuted sequences were returned to the pool, replacing the permuted originals. This procedure was used to generate Extended Data Fig. 1.

In addition to the RdRPs that could be assigned to previously identified clades at different depths of the phylogenetic tree, we attempted to detect putative highly divergent RdRPs. First, all long RNA contigs (>1,200 nucleotides; 10,813 contigs altogether) from the virome were translated stop-to-stop in 6 frames, and any which encoded open reading frames for more than 400 amino acids were selected and clustered by sequence similarity. The 37 profiles constructed from the resulting cluster alignments of 10 or more sequences were used as queries to search sequence databases with HHPred search. No RdRPs were found among these clusters. Second, open reading frames derived from 33 of the longest contigs in our dataset were analysed one at a time using HHPred; this procedure resulted in the identification of 13 singleton RdRP sequences (this analysis is too time consuming to perform on all potential RdRP-bearing sequences).

### DNA viruses in the Yangshan virome

The nucleotide sequences of DNA viruses were identified by comparing position-specific scoring matrices for the respective capsid proteins to the 6-frame translated sequences of the DNA metagenomic contigs using PSI-BLAST. The set of scoring matrices consists of 200 profiles derived from multiple alignments of capsid and coat proteins of eukaryotic, bacterial and archaeal DNA viruses. Of these, 98 alignments were taken from National Center for Biotechnology Information Conserved Domains Database^74^ and 102 were developed in-house^75–77^. PSI-BLAST searches initiated by these profiles were competed against other, unrelated PFAM profiles in the Conserved Domains Database. Significant (*e*-value < 0.0001) hits were recorded; contigs containing these hits were tentatively assigned to the respective virus group. Sampled sequences were manually curated using HHPred to verify or correct assignments.

For many of the Polinton-like virus contigs, the best hits in the NR database are (erroneously) annotated as bacteria assembled from marine metagenomes (for example MAO23883.1/NZRF01000276.1, matching NODE_13251 contig). These ‘bacterial’ assemblies probably contain numerous fragments derived from the marine virome. All nucleo-cytoplasmic large DNA virus (NCDLV) contigs were found to be highly similar to *Phycodnaviridae* (for example YP_004062106.1/NC_014767.1 matching NODE_1923356 contig). Many of these also have close matches in ‘bacterial’ assemblies from marine metagenomes (MAB60321.1/NYUE01000104.1). All four parvovirus contigs showed only distant similarity (about 30% protein identity) to vertebrate parvoviruses (for example APQ44761.1/KY053092.1 matching NODE_10537 contig), suggesting that these are viruses of unidentified hosts rather than vertebrate virus contaminants.

### Identification and annotation of protein domains

To identify protein domains, we performed sensitive profile–profile comparisons using HHsearch^72^. The identification procedure was run iteratively. First, profiles for each in silico-translated protein sequence were generated by performing one iteration against uniclust30_2018_08 database^78^ with HHblits^79^. The generated profiles were then compared against the previous generated RNA virus profile database^42^. Protein regions longer than 100 residues that did not display significant hits were extracted and clustered with CLANS^80^. Groups containing at least five members were identified using convex clustering algorithm implicated in CLANS, aligned with MUSCLE^71^, annotated when possible and added to the RNA virus profile database. In addition, extracted protein regions were searched against the European Bioinformatics Institute metagenomics database^81^, supplemented with the RNA virus protein sequences from the current study by performing one iteration of Jackhmmer^82^. Profiles with statistically significant hits (probability >95%) were annotated and added to the RNA virus profile database. Finally, domain identification procedure was repeated using the updated RNA virus profile database.

### CRISPR-spacer search

CRISPR spacers (363,468 unique spacers) were matched against the set of oceanic virus contigs; 90% identity, 90% coverage criteria were used for matches, as previously described^83^.

### Virus abundance

The abundances of viruses present in each virus cluster were calculated by mapping DNA-subtracted RNA sequencing reads back to RdRP-bearing contigs using bowtie2^84^. A bowtie2 index was generated from the combined non-redundant contigs assembled from all RNA libraries, and bowtie2 was then used to map reads from each experiment back to these contigs. All RdRP-bearing contigs were more than 95% covered by mapped reads. The abundance of each contig was calculated as mapped reads per kilobase per million (RPKM) total reads in the library.

**Extended Data Fig. 10:**
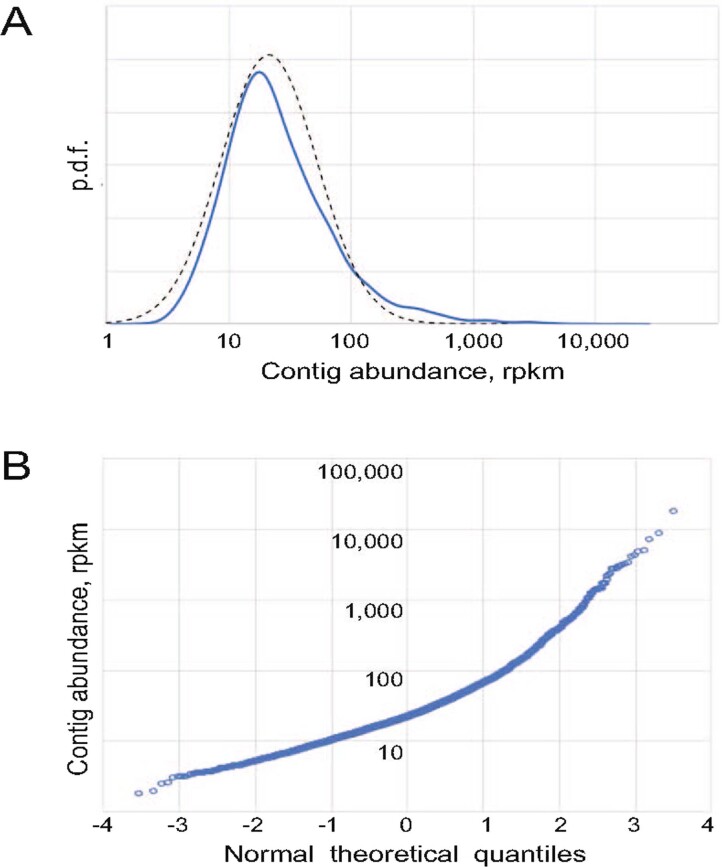
Distribution of contig abundances. (**a**) Probability density function (p.d.f.) for contig abundances (n = 4571 non-identical contigs. The dotted line plots the log-normal distribution with the same median and interquartile distance. (**b**) Quantile-quantile (Q-Q) plot of the distribution of contig abundances (n = 4571 non-identical contigs) versus the standard normal distribution. The figure shows that at the first approximation the distribution of contig abundances follows the log-normal distribution (typical in complex environments), but the deviations (a pronounced heavy tail of high values) hints on a dynamic environment producing superabundant viral blooms. Source data for panels (A) and (B) are presented in Source Data Figs. 1 and 2, respectively. Source data

The distribution of contig abundances covers several orders of magnitude, is unimodal with a peak at ~18 RPKM and a median of 21.3 RPKM, and resembles a log-normal distribution (Extended Data Fig. 10a). However, the distribution is skewed such that the highly abundant assemblies are more abundant than expected from the log-normal distribution (Extended Data Fig. 10b).

The top 20 contigs had at least 10× greater coverage than the median RPKM value (Supplementary Dataset 4). The most abundant virus, a member of OV.89 in the Tombus-like clade, was more than 800-fold over-represented compared to the median. The next three most abundant viruses were those from the Picorna-like aquatic/*Marnaviridae* and Zhao-like clades, all probably hosted by eukaryotic phytoplankton.

The contigs were then grouped by cluster or by clade to identify over-represented lineages (Supplementary Dataset 4). A pronounced correspondence between the diversity and abundance of the virus clusters was observed. The most abundant cluster was also the most diverse one (OV.1 of the Aquatic picorna-like/*Marnaviridae* clade), suggesting an overall prevalence of eukaryotic aquatic plankton. The Tombus-like clade was also well represented, largely, due to the most abundant virus mentioned above. The Yan-like and Zhao-like clades within the Yangshan assemblage contained several highly abundant viruses as well. Finally, several ourmia-like (OV.6) and levi-like viruses were prominent, particularly, the most abundant putative +RNA phage (OV.81).

### Reporting Summary

Further information on research design is available in the Nature Research Reporting Summary linked to this article.

## Supplementary information

Reporting Summary

Supplementary Data 1 Operational clusters of the RdRP sequences identified in Yangshan RNA virome (OV.1 to OV.323; from largest to smallest) and used for the further phylogenetic analysis.

Supplementary Data 2 Clade-specific phylogenies for Yangshan RNA viruses. Each tree contains representatives of the indicated clade (for example, Ov1 and Ov2) as well as phylogenetically close reference viruses. Genome maps for the sequences and some reference viruses are shown on the right. The functional domains are colour-coded and the key is provided at the bottom of each panel. MP, movement protein; RBP, RNA-binding protein; GTase, guanylyltransferase; rXXX.0, uncharacterized conserved domains.

Supplementary Data 3 The RdRP core domains in the Yangshan RNA virome encoded in alternative genetic codes.

Supplementary Data 4 The relative abundance of the virus contigs in the Yangshan RNA virome.

Supplementary Data 5 The list of full-length RdRPs in the Yangshan RNA virome. Correspondence between the amino acid sequence IDs (‘orf.nnn’), contig IDs (‘NODE_xxx’), coordinates of the RdRP core in the nucleotide sequence and GenBank contig IDs are shown.

## Data Availability

The sequence data analysed in this work are publicly available at the National Center for Biotechnology Information (NCBI) sequence databases under Bioproject PRJNA605028, accession JAAOEH000000000 (RNA virome) and Bioproject PRJNA610033, accession JAAOEI000000000 (DNA virome). Additional data (including alignments, trees and domain assignment) are available with no restrictions at ftp://ftp.ncbi.nih.gov/pub/wolf/_suppl/yangshan. Limited quantities of the remaining biological materials are available upon request. Source data are provided with this paper.

## Source data

Source Data Extended Data Fig. 10 Source data for Extended Data Fig. 10.

## Code availability

Custom software code is available at ftp://ftp.ncbi.nih.gov/pub/wolf/_suppl/yangshan with no restrictions.
