# Supplementary material for: Doubling of the known set of RNA viruses by metagenomic analysis of an aquatic virome
Source: Nat Microbiol. 2020 Jul 20;5(10):1262–70. doi: 10.1038/s41564-020-0755-4 (PMC7508674; doi:10.1038/s41564-020-0755-4)
Supplement: Supplementary file 1 — Reporting Summary [file 41564_2020_755_MOESM1_ESM.pdf]

nature research

Corresponding author(s): Valerian V. Dolja, Eugene V. Koonin

Last updated by author(s): May 1, 2020

Reporting Summary

Nature Research wishes to improve the reproducibility of the work that we publish. This form provides structure for consistency and transparency in reporting. For further information on Nature Research policies, see Authors & Referees and the Editorial Policy Checklist.

Statistics

For all statistical analyses, confirm that the following items are present in the figure legend, table legend, main text, or Methods section.

n/a	Confirmed	
☒	☐	The exact sample size (n) for each experimental group/condition, given as a discrete number and unit of measurement
☒	☐	A statement on whether measurements were taken from distinct samples or whether the same sample was measured repeatedly
☒	☐	The statistical test(s) used AND whether they are one- or two-sided<br>*Only common tests should be described solely by name; describe more complex techniques in the Methods section.*
☒	☐	A description of all covariates tested
☒	☐	A description of any assumptions or corrections, such as tests of normality and adjustment for multiple comparisons
☒	☐	A full description of the statistical parameters including central tendency (e.g. means) or other basic estimates (e.g. regression coefficient) AND variation (e.g. standard deviation) or associated estimates of uncertainty (e.g. confidence intervals)
☒	☐	For null hypothesis testing, the test statistic (e.g. F, t, r) with confidence intervals, effect sizes, degrees of freedom and P value noted<br>*Give P values as exact values whenever suitable.*
☒	☐	For Bayesian analysis, information on the choice of priors and Markov chain Monte Carlo settings
☒	☐	For hierarchical and complex designs, identification of the appropriate level for tests and full reporting of outcomes
☒	☐	Estimates of effect sizes (e.g. Cohen's d, Pearson's r), indicating how they were calculated

Our web collection on statistics for biologists contains articles on many of the points above.

Software and code

Policy information about availability of computer code

Data collection	Hiseq 2500: Illumina Real Time Analysis (RTA) v1.17.21.3
Data analysis	The following software was used for data analysis: blast 2.10.0; MUSCLE v3.7; uclust v1.2.22q; MAFFT v7.307; MMseqs2; FastTree 2.1.4; HHtools 1.5.1; SPADES v3.11.1; SPADES v3.7; bowtie2. Custom scripts were uploaded to ftp://ftp.ncbi.nih.gov/pub/wolf/_suppl/yangshan (software.tgz)

For manuscripts utilizing custom algorithms or software that are central to the research but not yet described in published literature, software must be made available to editors/reviewers. We strongly encourage code deposition in a community repository (e.g. GitHub). See the Nature Research guidelines for submitting code & software for further information.

Data

Policy information about availability of data

All manuscripts must include a data availability statement. This statement should provide the following information, where applicable:

- Accession codes, unique identifiers, or web links for publicly available datasets
- A list of figures that have associated raw data
- A description of any restrictions on data availability

The sequence data analyzed in this work have been submitted to Genbank under Accessions JAAOEH000000000 and JAAOEI000000000. Additional data (alignments, trees, etc.) is available at ftp://ftp.ncbi.nih.gov/pub/wolf/_suppl/yangshan FTP directory. Limited quantities of remaining biological materials are available upon request.

Field-specific reporting

Please select the one below that is the best fit for your research. If you are not sure, read the appropriate sections before making your selection.

☐ Life sciences ☐ Behavioural & social sciences ☒ Ecological, evolutionary & environmental sciences

For a reference copy of the document with all sections, see nature.com/documents/nr-reporting-summary-flat.pdf

Ecological, evolutionary & environmental sciences study design

All studies must disclose on these points even when the disclosure is negative.

Study description	This study is a survey of planktonic marine RNA viruses from a single large sample of seawater collected from three distinct sites in Yangshan Deep-Water Harbor in Shanghai, China.
Research sample	A single combined 100 liter sample of seawater from three distinct sites in Yangshan Deep-Water Harbor, Shanghai, China on October 31, 2017. Cellular organisms (bacteria, phytoplankton, zooplankton etc.) were excluded from the sample to the best of our ability in order to obtain a pure RNA dataset representing the viral fraction, uncontaminated by abundant cellular RNAs such as transfer or ribosomal RNAs. This also allows us to focus on the discovery of previously unknown putatively viral genetic elements, with less interference from unknown cellular genes.
Sampling strategy	The 100 liter water sample was initially settled at 4°C for 12 hours, and viruses were isolated following the TFF procedures. The concentrated viral particles were stored at -80°C before use. The absence of bacterial or cellular contamination in the filtrate was confirmed by transmission electron microscopy. The purified viral fraction was split into ~10 tubes, and one tube each was used for extraction of putative viral DNA, and viral RNA with or without subsequent treatment with DNase I. The size of the sample was not predetermined. The sample was chosen to be large enough to contain a diversity of planktonic RNA viruses based on prior experience and the literature. The sample sizes were sufficient as evidenced by the vast diversity of RNA viruses discovered in this study.
Data collection	Viral RNA and DNA were sequenced in parallel by Biozeron (Shanghai), and the RNA datasets were further purified in silico by study authors by subtracting sequences that were present in both the RNA and DNA datasets. All analyses reported in the manuscript were based on this purified RNA dataset.
Timing and spatial scale	All samples were collected on October 31, 2017. The precise sampling sites are noted in Figure 1.
Data exclusions	Only the sequencing reads present both in the RNA and DNA datasets were removed. This was done to obtain a "pure" RNA dataset (eliminating contaminating cellular transcripts and sequences of co-purifying DNA viruses). The subtraction criteria were pre-established, and various k-mer lengths of putative matches were tested to validate the pre-established k-mer length cutoff based on theoretical calculations and total number of reads in the raw datasets. As anticipated by a priori calculations, while subtraction using 20-mers resulted in gross over- filtering, 25- and 30-mers resulted in very similar numbers of reads removed, indicating that the subtraction procedure was not sensitive to the k-mer length chosen.
Reproducibility	A small fraction of the sample was sequenced using a different sequencing method. While the bulk of the RNA sequencing data was obtained using the TruSeq RNA library prep kit, a smaller dataset was also generated using the Clontech SMARTer Stranded Total RNAseq kit, since it uses a very different method of priming the reverse-transcription reaction (template-switching reverse transcription as opposed to random hexamer priming). The datasets were found to be substantially similar and were combined for all downstream analyses.
Randomization	Only one sample was obtained and hence no randomization is possible.
Blinding	This study reports the discovery of thousands of previously unknown RNA viruses; no blinding is relevant.

Did the study involve field work? ☒ Yes ☐ No

Field work, collection and transport

Field conditions	Temperature: 18.6-18.8° C, salinity: 9.5-10.1‰, total dissolved solid (TDS): 14.5 g/L, pH: 8.1.
Location	Surface seawater was collected from Yangshan Harbor, Shanghai, China (latitude: 30°35.729′-30°36.182′, longitude: 122° 05.371′-122°05.897′). Sampling depth: 1-8 m and water depth: 15 m (in average).
Access and import/export	There is no specific forbiddance in local and national laws for Chinese researchers who access Yangshan Harbor and collect seawater samples only for science and study. Currently, official permits from authority are not needed.
Disturbance	No disturbance caused by this study.

Reporting for specific materials, systems and methods

We require information from authors about some types of materials, experimental systems and methods used in many studies. Here, indicate whether each material, system or method listed is relevant to your study. If you are not sure if a list item applies to your research, read the appropriate section before selecting a response.

Materials & experimental systems

n/a	Involved in the study
☒	☐ Antibodies
☒	☐ Eukaryotic cell lines
☒	☐ Palaeontology
☒	☐ Animals and other organisms
☒	☐ Human research participants
☒	☐ Clinical data

Methods

n/a	Involved in the study
☒	☐ ChIP-seq
☒	☐ Flow cytometry
☒	☐ MRI-based neuroimaging
