# Supplementary material for: Doubling of the known set of RNA viruses by metagenomic analysis of an aquatic virome
Source: Nat Microbiol. 2020 Jul 20;5(10):1262–70. doi: 10.1038/s41564-020-0755-4 (PMC7508674; doi:10.1038/s41564-020-0755-4)
Supplement: Supplementary file 3 — Clade-specific phylogenies for Yangshan RNA viruses. Each tree contains representatives of the indicated clade (for example, Ov1 and Ov2) as well as phylogenetically close reference viruses. Genome maps for the sequences and some reference viruses are shown on the right. The functional domains are colour-coded and the key is provided at the bottom of each panel. MP, movement protein; RBP, RNA-binding protein; GTase, guanylyltransferase; rXXX.0, uncharacterized conserved domains. [file 41564_2020_755_MOESM3_ESM.pdf]

Ov1

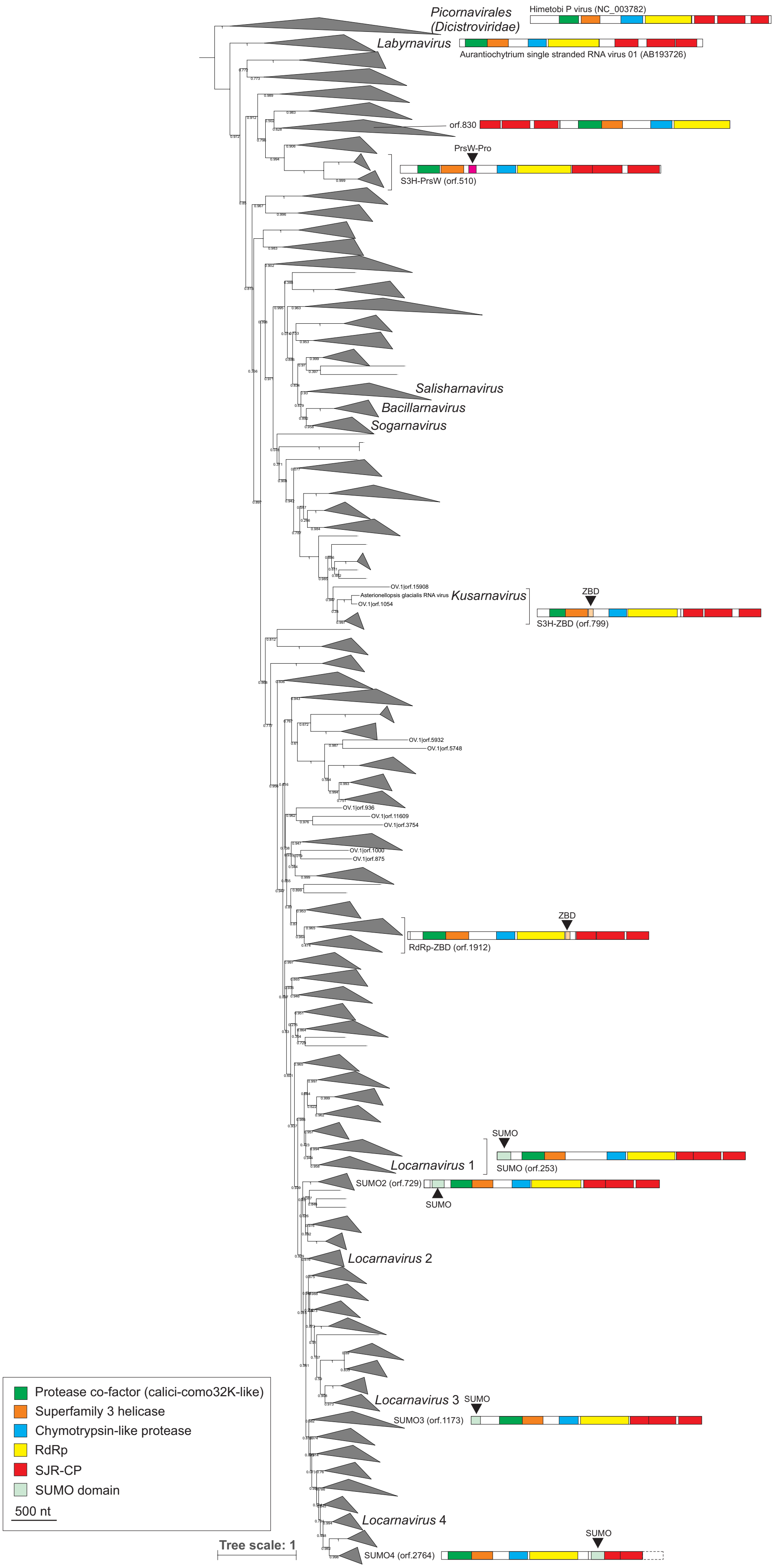

Picornavirales
(Dicistroviridae)
Himetobi P virus (NC_003782)
Labyrnavirus
Aurantiochytrium single stranded RNA virus 01 (AB193726)
orf.830
PrsW-Pro
S3H-PrsW (orf.510)
Salisharnavirus
Bacillarnavirus
Sogarnavirus
OV.1|orf.15908
Asterionellopsis glacialis RNA virus
OV.1|orf.1054
Kusarnavirus
ZBD
S3H-ZBD (orf.799)
OV.1|orf.5932
OV.1|orf.5748
OV.1|orf.936
OV.1|orf.11609
OV.1|orf.3754
OV.1|orf.1000
OV.1|orf.875
ZBD
RdRp-ZBD (orf.1912)
SUMO
Locarnavirus 1
SUMO (orf.253)
SUMO2 (orf.729)
SUMO
Locarnavirus 2
Locarnavirus 3
SUMO
SUMO3 (orf.1173)
Locarnavirus 4
SUMO
SUMO4 (orf.2764)
Protease co-factor (calici-como32K-like)
Superfamily 3 helicase
Chymotrypsin-like protease
RdRp
SJR-CP
SUMO domain
500 nt
Tree scale: 1

Ov2

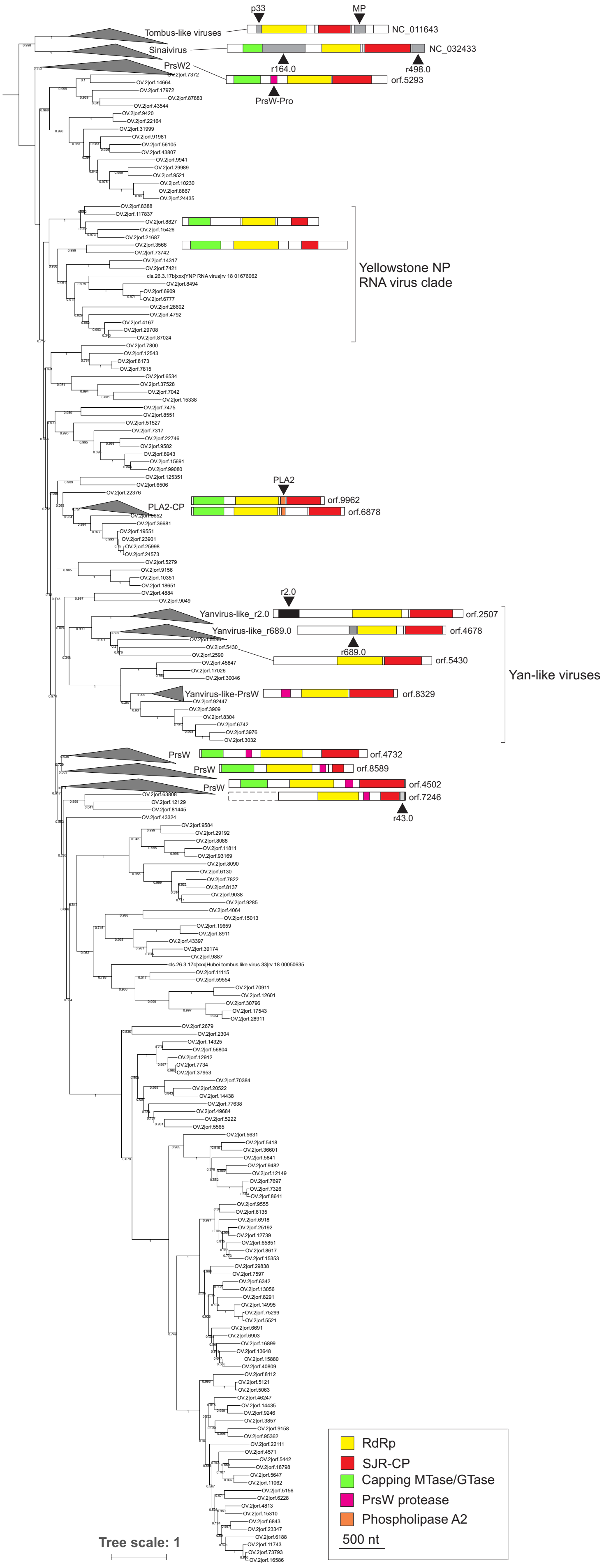
p33
MP
Tombus-like viruses
NC_011643
Sinaivirus
NC_032433
r164.0
r498.0
PrsW2
orf.5293
PrsW-Pro
Yellowstone NP
RNA virus clade
PLA2
orf.9962
PLA2-CP
orf.6878
r2.0
Yanvirus-like_r2.0
orf.2507
Yanvirus-like_r689.0
orf.4678
r689.0
orf.5430
Yan-like viruses
Yanvirus-like-PrsW
orf.8329
PrsW
orf.4732
PrsW
orf.8589
PrsW
orf.4502
orf.7246
r43.0
Tree scale: 1
RdRp
SJR-CP
Capping MTase/GTase
PrsW protease
Phospholipase A2
500 nt

Ov3

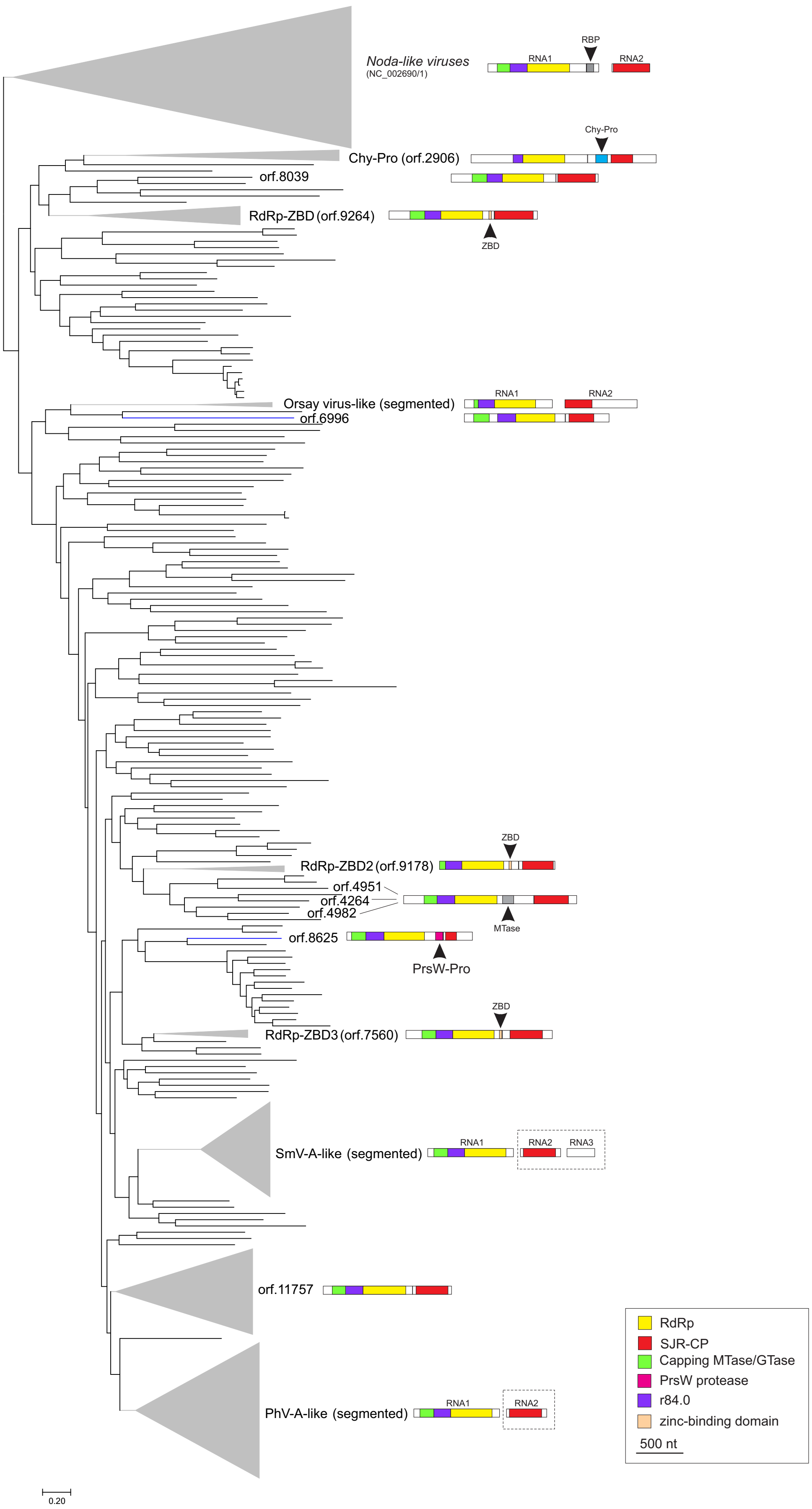
Noda-like viruses
(NC_002690/1)
RNA1
RBP
RNA2
Chy-Pro
Chy-Pro (orf.2906)
orf.8039
RdRp-ZBD (orf.9264)
ZBD
Orsay virus-like (segmented)
orf.6996
RNA1
RNA2
ZBD
RdRp-ZBD2 (orf.9178)
orf.4951
orf.4264
orf.4982
MTase
orf.8625
PrsW-Pro
ZBD
RdRp-ZBD3 (orf.7560)
SmV-A-like (segmented)
RNA1
RNA2
RNA3
orf.11757
PhV-A-like (segmented)
RNA1
RNA2
RdRp
SJR-CP
Capping MTase/GTase
PrsW protease
r84.0
zinc-binding domain
500 nt
0.20

Ov4

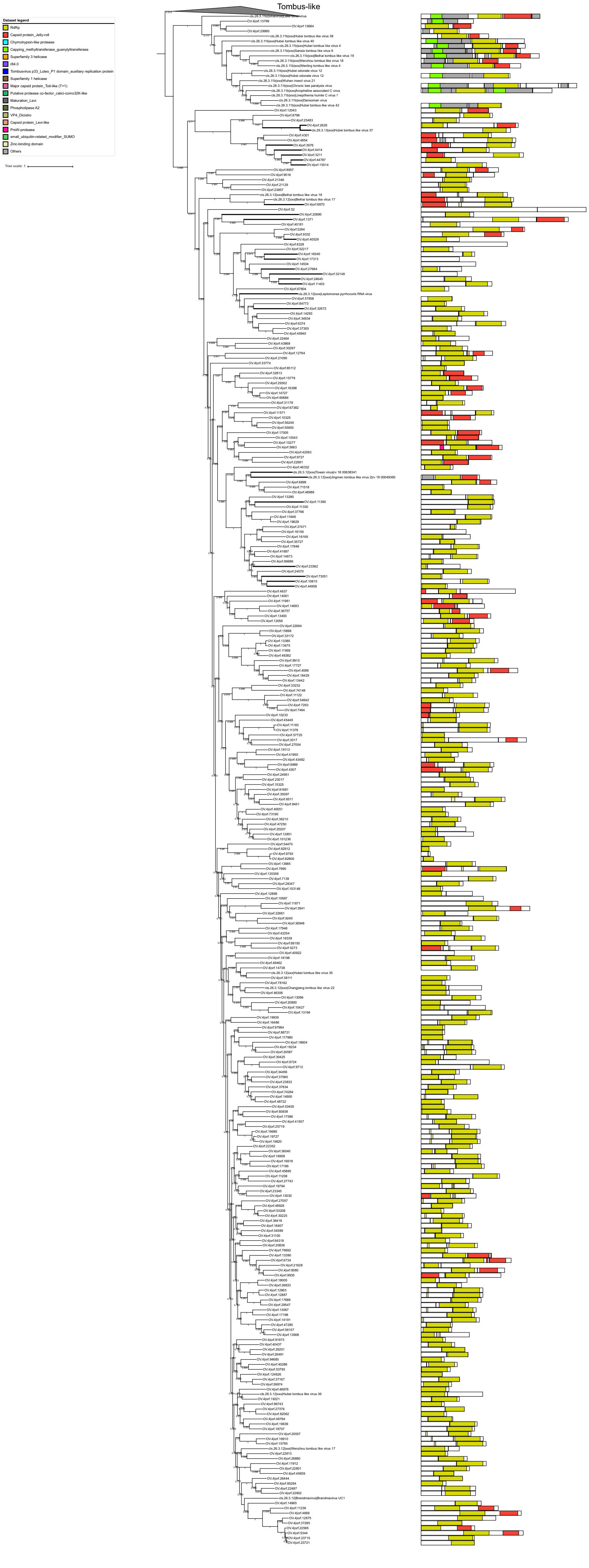
Dataset legend
RdRp
Capsid protein_Jelly-roll
Chymotrypsin-like protease
Capping_methyltransferase_guanylyltransferase
Superfamily 3 helicase
r84.0
Tombusvirus p33_Luteo_P1 domain_auxiliary replication protein
Superfamily 1 helicase
Major capsid protein_Toti-like (T=1)
Putative protease co-factor_calici-como32K-like
Maturation_Levi
Phospholipase A2
VP4_Dicistro
Capsid protein_Levi-like
PrsW-protease
small_ubiquitin-related_modifier_SUMO
Zinc-binding domain
Others
Tree scale: 1
Tombus-like

Ov5

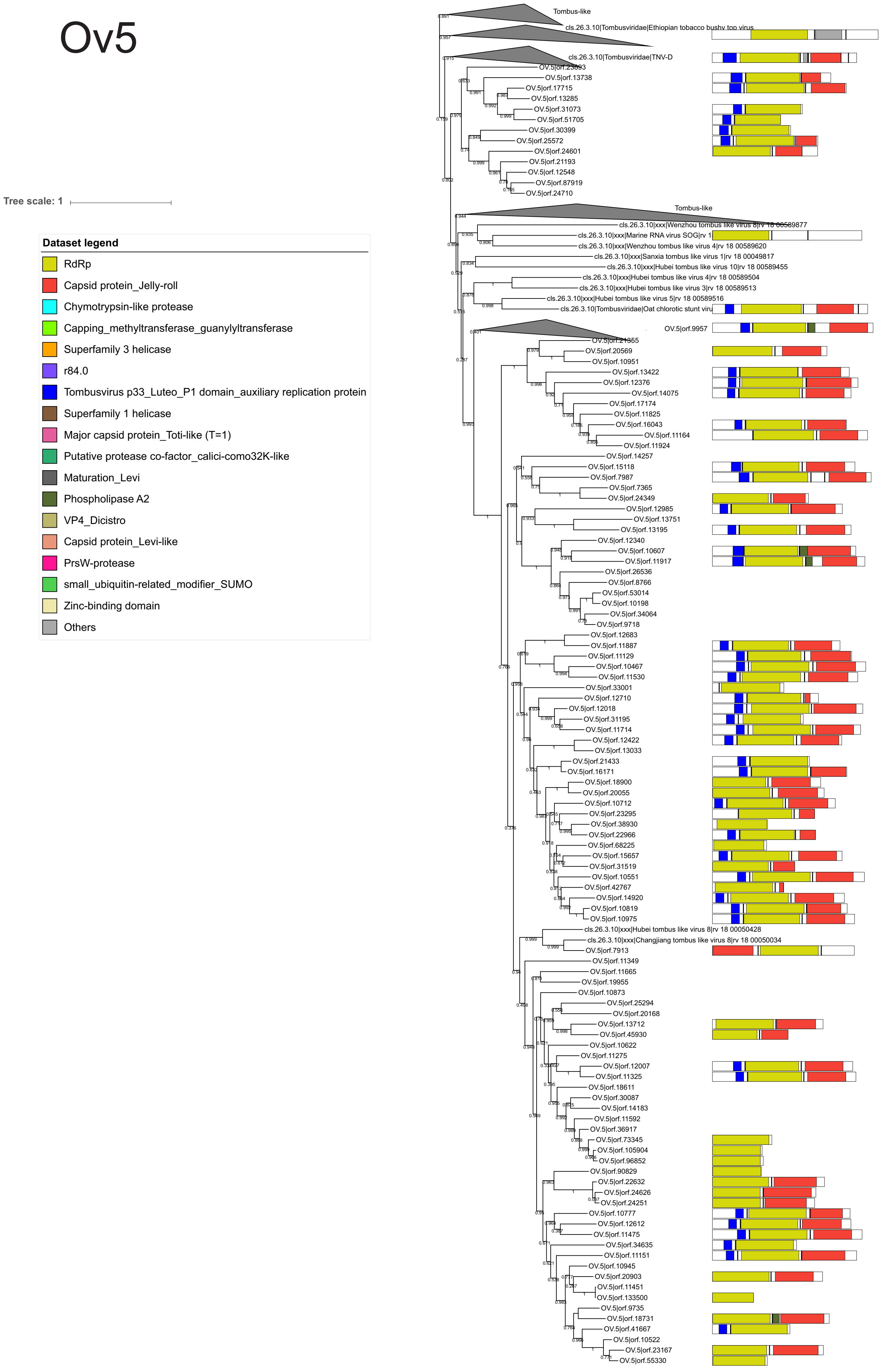

Tree scale: 1
Dataset legend
RdRp
Capsid protein_Jelly-roll
Chymotrypsin-like protease
Capping_methyltransferase_guanylyltransferase
Superfamily 3 helicase
r84.0
Tombusvirus p33_Luteo_P1 domain_auxiliary replication protein
Superfamily 1 helicase
Major capsid protein_Toti-like (T=1)
Putative protease co-factor_calici-como32K-like
Maturation_Levi
Phospholipase A2
VP4_Dicistro
Capsid protein_Levi-like
PrsW-protease
small_ubiquitin-related_modifier_SUMO
Zinc-binding domain
Others
Tombus-like
cls.26.3.10|Tombusviridae|Ethiopian tobacco bushy top virus
cls.26.3.10|Tombusviridae|TNV-D
OV.5|orf.23693
OV.5|orf.13738
OV.5|orf.17715
OV.5|orf.13285
OV.5|orf.31073
OV.5|orf.51705
OV.5|orf.30399
OV.5|orf.25572
OV.5|orf.24601
OV.5|orf.21193
OV.5|orf.12548
OV.5|orf.87919
OV.5|orf.24710
Tombus-like
cls.26.3.10|xxx|Wenzhou tombus like virus 8|rv 18 00589877
cls.26.3.10|xxx|Marine RNA virus SOG|rv 1
cls.26.3.10|xxx|Wenzhou tombus like virus 4|rv 18 00589620
cls.26.3.10|xxx|Sanxia tombus like virus 1|rv 18 00049817
cls.26.3.10|xxx|Hubei tombus like virus 10|rv 18 00589455
cls.26.3.10|xxx|Hubei tombus like virus 4|rv 18 00589504
cls.26.3.10|xxx|Hubei tombus like virus 3|rv 18 00589513
cls.26.3.10|xxx|Hubei tombus like virus 5|rv 18 00589516
cls.26.3.10|Tombusviridae|Oat chlorotic stunt viru
OV.5|orf.9957
OV.5|orf.21365
OV.5|orf.20569
OV.5|orf.10951
OV.5|orf.13422
OV.5|orf.12376
OV.5|orf.14075
OV.5|orf.17174
OV.5|orf.11825
OV.5|orf.16043
OV.5|orf.11164
OV.5|orf.11924
OV.5|orf.14257
OV.5|orf.15118
OV.5|orf.7987
OV.5|orf.7365
OV.5|orf.24349
OV.5|orf.12985
OV.5|orf.13751
OV.5|orf.13195
OV.5|orf.12340
OV.5|orf.10607
OV.5|orf.11917
OV.5|orf.26536
OV.5|orf.8766
OV.5|orf.53014
OV.5|orf.10198
OV.5|orf.34064
OV.5|orf.9718
OV.5|orf.12683
OV.5|orf.11887
OV.5|orf.11129
OV.5|orf.10467
OV.5|orf.11530
OV.5|orf.33001
OV.5|orf.12710
OV.5|orf.12018
OV.5|orf.31195
OV.5|orf.11714
OV.5|orf.12422
OV.5|orf.13033
OV.5|orf.21433
OV.5|orf.16171
OV.5|orf.18900
OV.5|orf.20055
OV.5|orf.10712
OV.5|orf.23295
OV.5|orf.38930
OV.5|orf.22966
OV.5|orf.68225
OV.5|orf.15657
OV.5|orf.31519
OV.5|orf.10551
OV.5|orf.42767
OV.5|orf.14920
OV.5|orf.10819
OV.5|orf.10975
cls.26.3.10|xxx|Hubei tombus like virus 8|rv 18 00050428
cls.26.3.10|xxx|Changjiang tombus like virus 8|rv 18 00050034
OV.5|orf.7913
OV.5|orf.11349
OV.5|orf.11665
OV.5|orf.19955
OV.5|orf.10873
OV.5|orf.25294
OV.5|orf.20168
OV.5|orf.13712
OV.5|orf.45930
OV.5|orf.10622
OV.5|orf.11275
OV.5|orf.12007
OV.5|orf.11325
OV.5|orf.18611
OV.5|orf.30087
OV.5|orf.14183
OV.5|orf.11592
OV.5|orf.36917
OV.5|orf.73345
OV.5|orf.105904
OV.5|orf.96852
OV.5|orf.90829
OV.5|orf.22632
OV.5|orf.24626
OV.5|orf.24251
OV.5|orf.10777
OV.5|orf.12612
OV.5|orf.11475
OV.5|orf.34635
OV.5|orf.11151
OV.5|orf.10945
OV.5|orf.20903
OV.5|orf.11451
OV.5|orf.133500
OV.5|orf.9735
OV.5|orf.18731
OV.5|orf.41667
OV.5|orf.10522
OV.5|orf.23167
OV.5|orf.55330

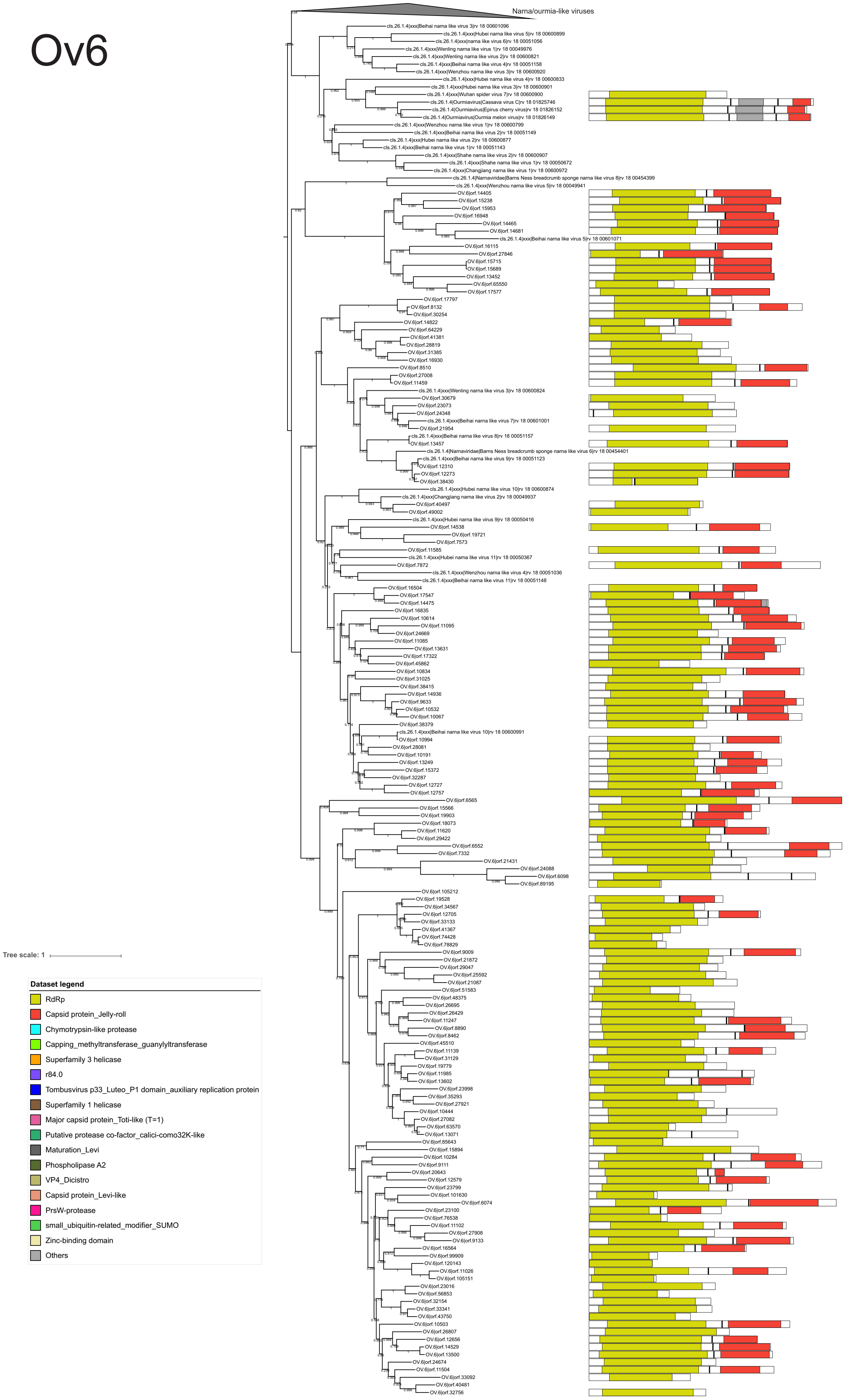
Ov6
Narna/ourmia-like viruses
Tree scale: 1
Dataset legend
RdRp
Capsid protein_Jelly-roll
Chymotrypsin-like protease
Capping_methyltransferase_guanylyltransferase
Superfamily 3 helicase
r84.0
Tombusvirus p33_Luteo_P1 domain_auxiliary replication protein
Superfamily 1 helicase
Major capsid protein_Toti-like (T=1)
Putative protease co-factor_calici-como32K-like
Maturation_Levi
Phospholipase A2
VP4_Dicistro
Capsid protein_Levi-like
PrsW-protease
small_ubiquitin-related_modifier_SUMO
Zinc-binding domain
Others

Ov7

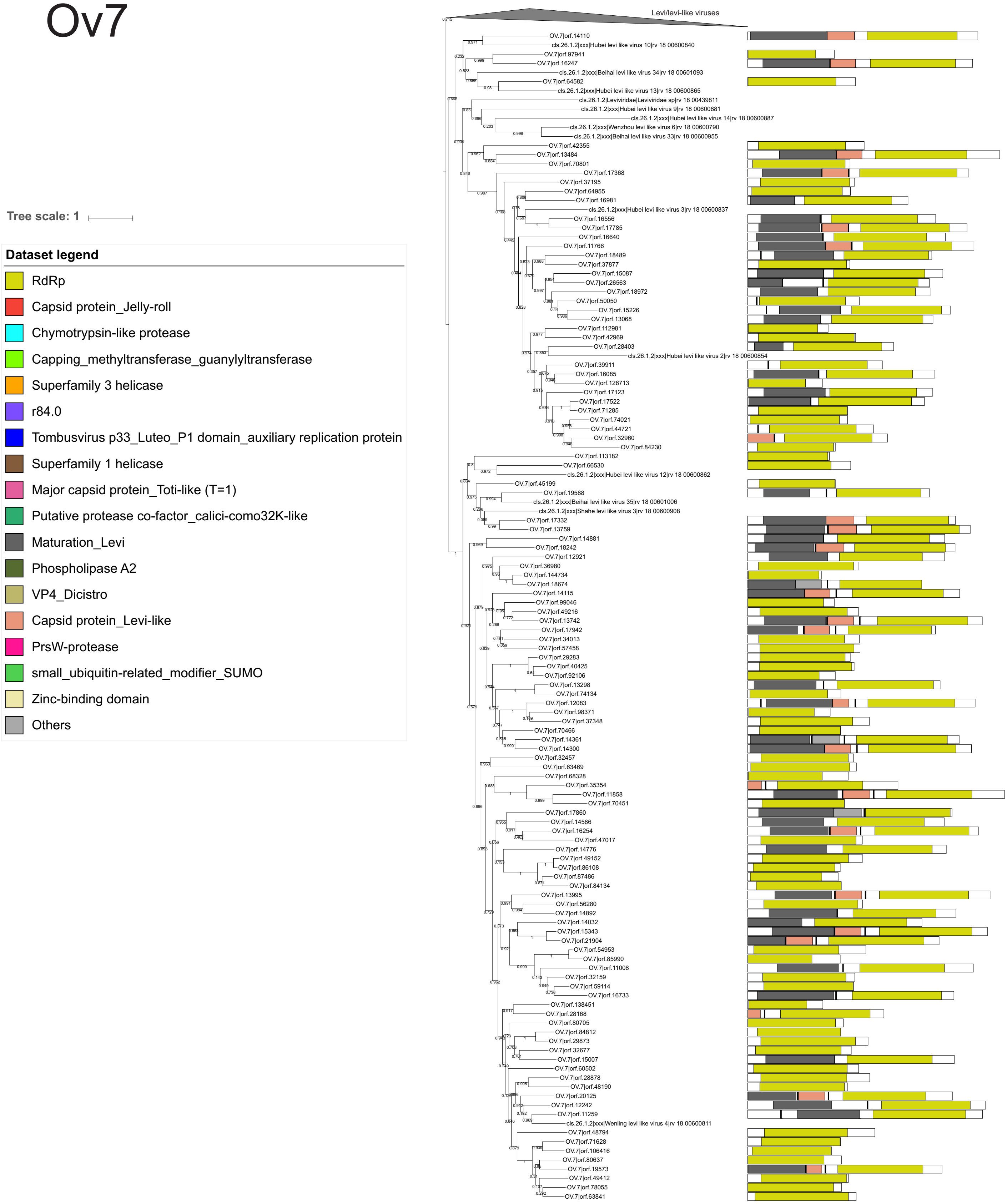

Levi/levi-like viruses
Tree scale: 1
Dataset legend
RdRp
Capsid protein_Jelly-roll
Chymotrypsin-like protease
Capping_methyltransferase_guanylyltransferase
Superfamily 3 helicase
r84.0
Tombusvirus p33_Luteo_P1 domain_auxiliary replication protein
Superfamily 1 helicase
Major capsid protein_Toti-like (T=1)
Putative protease co-factor_calici-como32K-like
Maturation_Levi
Phospholipase A2
VP4_Dicistro
Capsid protein_Levi-like
PrsW-protease
small_ubiquitin-related_modifier_SUMO
Zinc-binding domain
Others

Ov8

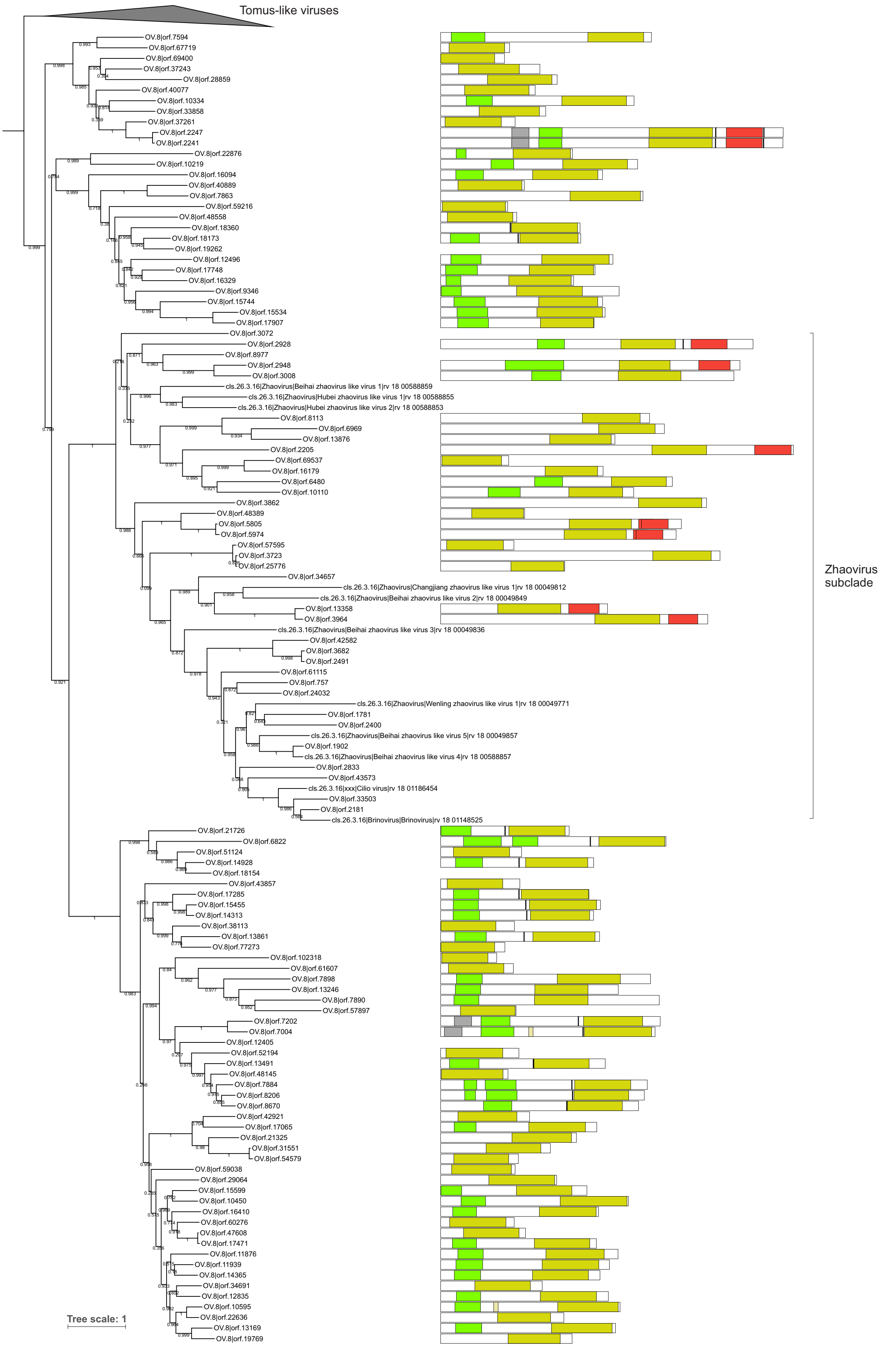

Tomus-like viruses
OV.8|orf.7594
OV.8|orf.67719
OV.8|orf.69400
OV.8|orf.37243
OV.8|orf.28859
OV.8|orf.40077
OV.8|orf.10334
OV.8|orf.33858
OV.8|orf.37261
OV.8|orf.2247
OV.8|orf.2241
OV.8|orf.22876
OV.8|orf.10219
OV.8|orf.16094
OV.8|orf.40889
OV.8|orf.7863
OV.8|orf.59216
OV.8|orf.48558
OV.8|orf.18360
OV.8|orf.18173
OV.8|orf.19262
OV.8|orf.12496
OV.8|orf.17748
OV.8|orf.16329
OV.8|orf.9346
OV.8|orf.15744
OV.8|orf.15534
OV.8|orf.17907
OV.8|orf.3072
OV.8|orf.2928
OV.8|orf.8977
OV.8|orf.2948
OV.8|orf.3008
cls.26.3.16|Zhaovirus|Beihai zhaovirus like virus 1|rv 18 00588859
cls.26.3.16|Zhaovirus|Hubei zhaovirus like virus 1|rv 18 00588855
cls.26.3.16|Zhaovirus|Hubei zhaovirus like virus 2|rv 18 00588853
OV.8|orf.8113
OV.8|orf.6969
OV.8|orf.13876
OV.8|orf.2205
OV.8|orf.69537
OV.8|orf.16179
OV.8|orf.6480
OV.8|orf.10110
OV.8|orf.3862
OV.8|orf.48389
OV.8|orf.5805
OV.8|orf.5974
OV.8|orf.57595
OV.8|orf.3723
OV.8|orf.25776
OV.8|orf.34657
cls.26.3.16|Zhaovirus|Changjiang zhaovirus like virus 1|rv 18 00049812
cls.26.3.16|Zhaovirus|Beihai zhaovirus like virus 2|rv 18 00049849
OV.8|orf.13358
OV.8|orf.3964
cls.26.3.16|Zhaovirus|Beihai zhaovirus like virus 3|rv 18 00049836
OV.8|orf.42582
OV.8|orf.3682
OV.8|orf.2491
OV.8|orf.61115
OV.8|orf.757
OV.8|orf.24032
cls.26.3.16|Zhaovirus|Wenling zhaovirus like virus 1|rv 18 00049771
OV.8|orf.1781
OV.8|orf.2400
cls.26.3.16|Zhaovirus|Beihai zhaovirus like virus 5|rv 18 00049857
OV.8|orf.1902
cls.26.3.16|Zhaovirus|Beihai zhaovirus like virus 4|rv 18 00588857
OV.8|orf.2833
OV.8|orf.43573
cls.26.3.16|xxx|Cilio virus|rv 18 01186454
OV.8|orf.33503
OV.8|orf.2181
cls.26.3.16|Brinovirus|Brinovirus|rv 18 01148525
Zhaovirus subclade
OV.8|orf.21726
OV.8|orf.6822
OV.8|orf.51124
OV.8|orf.14928
OV.8|orf.18154
OV.8|orf.43857
OV.8|orf.17285
OV.8|orf.15455
OV.8|orf.14313
OV.8|orf.38113
OV.8|orf.13861
OV.8|orf.77273
OV.8|orf.102318
OV.8|orf.61607
OV.8|orf.7898
OV.8|orf.13246
OV.8|orf.7890
OV.8|orf.57897
OV.8|orf.7202
OV.8|orf.7004
OV.8|orf.12405
OV.8|orf.52194
OV.8|orf.13491
OV.8|orf.48145
OV.8|orf.7884
OV.8|orf.8206
OV.8|orf.8670
OV.8|orf.42921
OV.8|orf.17065
OV.8|orf.21325
OV.8|orf.31551
OV.8|orf.54579
OV.8|orf.59038
OV.8|orf.29064
OV.8|orf.15599
OV.8|orf.10450
OV.8|orf.16410
OV.8|orf.60276
OV.8|orf.47608
OV.8|orf.17471
OV.8|orf.11876
OV.8|orf.11939
OV.8|orf.14365
OV.8|orf.34691
OV.8|orf.12835
OV.8|orf.10595
OV.8|orf.22636
OV.8|orf.13169
OV.8|orf.19769
Tree scale: 1

Ov9

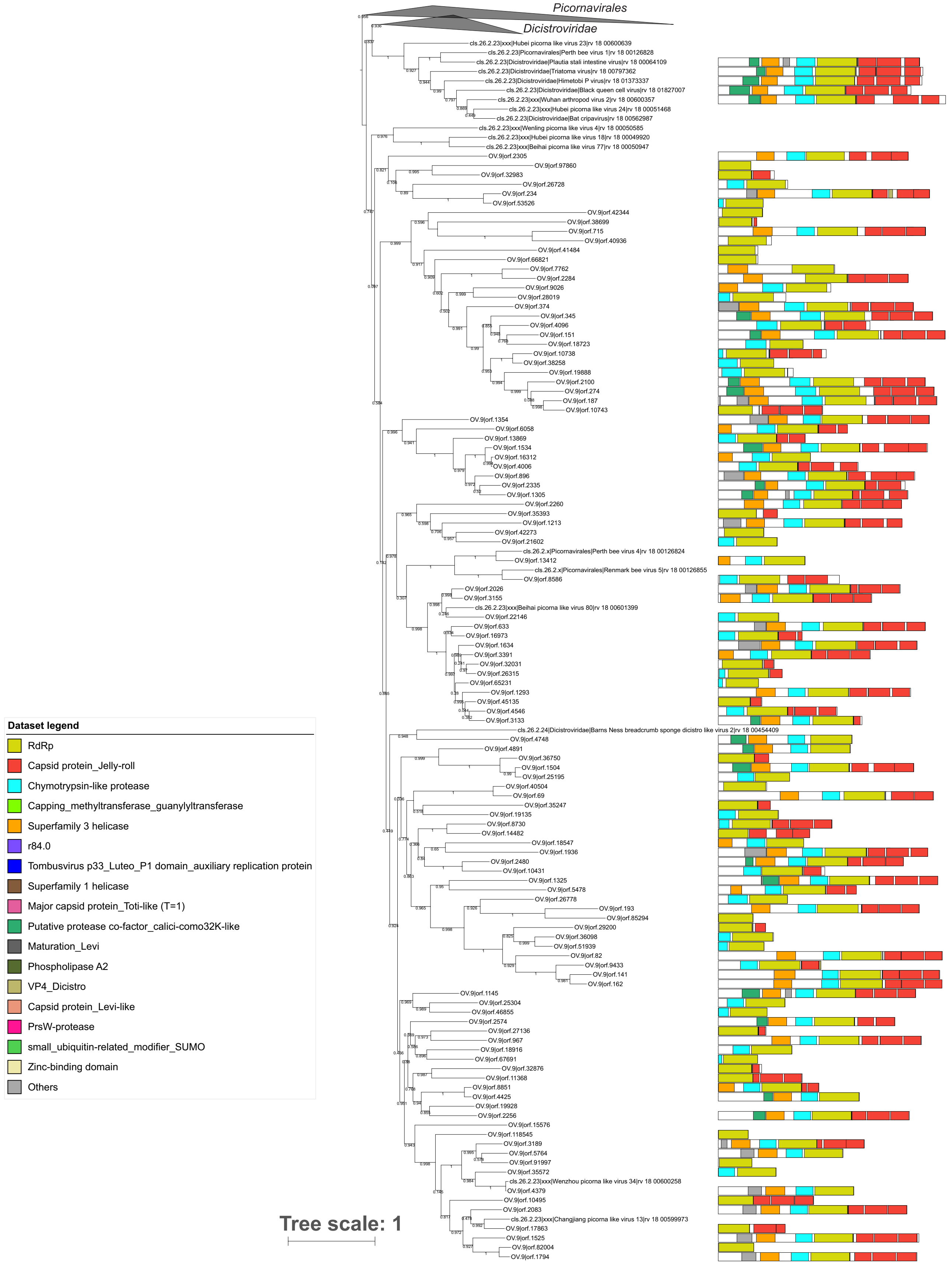

Picornavirales
Dicistroviridae
cls.26.2.23|xxx|Hubei picorna like virus 23|rv 18 00600639
cls.26.2.23|Picornavirales|Perth bee virus 1|rv 18 00126828
cls.26.2.23|Dicistroviridae|Plautia stali intestine virus|rv 18 00064109
cls.26.2.23|Dicistroviridae|Triatoma virus|rv 18 00797362
cls.26.2.23|Dicistroviridae|Himetobi P virus|rv 18 01373337
cls.26.2.23|Dicistroviridae|Black queen cell virus|rv 18 01827007
cls.26.2.23|xxx|Wuhan arthropod virus 2|rv 18 00600357
cls.26.2.23|xxx|Hubei picorna like virus 24|rv 18 00051468
cls.26.2.23|Dicistroviridae|Bat cripavirus|rv 18 00562987
cls.26.2.23|xxx|Wenling picorna like virus 4|rv 18 00050585
cls.26.2.23|xxx|Hubei picorna like virus 18|rv 18 00049920
cls.26.2.23|xxx|Beihai picorna like virus 77|rv 18 00050947
OV.9|orf.2305
OV.9|orf.97860
OV.9|orf.32983
OV.9|orf.26728
OV.9|orf.234
OV.9|orf.53526
OV.9|orf.42344
OV.9|orf.38699
OV.9|orf.715
OV.9|orf.40936
OV.9|orf.41484
OV.9|orf.66821
OV.9|orf.7762
OV.9|orf.2284
OV.9|orf.9026
OV.9|orf.28019
OV.9|orf.374
OV.9|orf.345
OV.9|orf.4096
OV.9|orf.151
OV.9|orf.18723
OV.9|orf.10738
OV.9|orf.38258
OV.9|orf.19888
OV.9|orf.2100
OV.9|orf.274
OV.9|orf.187
OV.9|orf.10743
OV.9|orf.1354
OV.9|orf.6058
OV.9|orf.13869
OV.9|orf.1534
OV.9|orf.16312
OV.9|orf.4006
OV.9|orf.896
OV.9|orf.2335
OV.9|orf.1305
OV.9|orf.2260
OV.9|orf.35393
OV.9|orf.1213
OV.9|orf.42273
OV.9|orf.21602
cls.26.2.x|Picornavirales|Perth bee virus 4|rv 18 00126824
OV.9|orf.13412
cls.26.2.x|Picornavirales|Renmark bee virus 5|rv 18 00126855
OV.9|orf.8586
OV.9|orf.2026
OV.9|orf.3155
cls.26.2.23|xxx|Beihai picorna like virus 80|rv 18 00601399
OV.9|orf.22146
OV.9|orf.633
OV.9|orf.16973
OV.9|orf.1634
OV.9|orf.3391
OV.9|orf.32031
OV.9|orf.26315
OV.9|orf.65231
OV.9|orf.1293
OV.9|orf.45135
OV.9|orf.4546
OV.9|orf.3133
cls.26.2.24|Dicistroviridae|Barns Ness breadcrumb sponge dicistro like virus 2|rv 18 00454409
OV.9|orf.4748
OV.9|orf.4891
OV.9|orf.36750
OV.9|orf.1504
OV.9|orf.25195
OV.9|orf.40504
OV.9|orf.69
OV.9|orf.35247
OV.9|orf.19135
OV.9|orf.8730
OV.9|orf.14482
OV.9|orf.18547
OV.9|orf.1936
OV.9|orf.2480
OV.9|orf.10431
OV.9|orf.1325
OV.9|orf.5478
OV.9|orf.26778
OV.9|orf.193
OV.9|orf.85294
OV.9|orf.29200
OV.9|orf.36098
OV.9|orf.51939
OV.9|orf.82
OV.9|orf.9433
OV.9|orf.141
OV.9|orf.162
OV.9|orf.1145
OV.9|orf.25304
OV.9|orf.46855
OV.9|orf.2574
OV.9|orf.27136
OV.9|orf.967
OV.9|orf.18916
OV.9|orf.67691
OV.9|orf.32876
OV.9|orf.11368
OV.9|orf.8851
OV.9|orf.4425
OV.9|orf.19928
OV.9|orf.2256
OV.9|orf.15576
OV.9|orf.118545
OV.9|orf.3189
OV.9|orf.5764
OV.9|orf.91997
OV.9|orf.35572
cls.26.2.23|xxx|Wenzhou picorna like virus 34|rv 18 00600258
OV.9|orf.4379
OV.9|orf.10495
OV.9|orf.2083
cls.26.2.23|xxx|Changjiang picorna like virus 13|rv 18 00599973
OV.9|orf.17863
OV.9|orf.1525
OV.9|orf.82004
OV.9|orf.1794
Tree scale: 1
Dataset legend
RdRp
Capsid protein_Jelly-roll
Chymotrypsin-like protease
Capping_methyltransferase_guanylyltransferase
Superfamily 3 helicase
r84.0
Tombusvirus p33_Luteo_P1 domain_auxiliary replication protein
Superfamily 1 helicase
Major capsid protein_Toti-like (T=1)
Putative protease co-factor_calici-como32K-like
Maturation_Levi
Phospholipase A2
VP4_Dicistro
Capsid protein_Levi-like
PrsW-protease
small_ubiquitin-related_modifier_SUMO
Zinc-binding domain
Others

Ov10

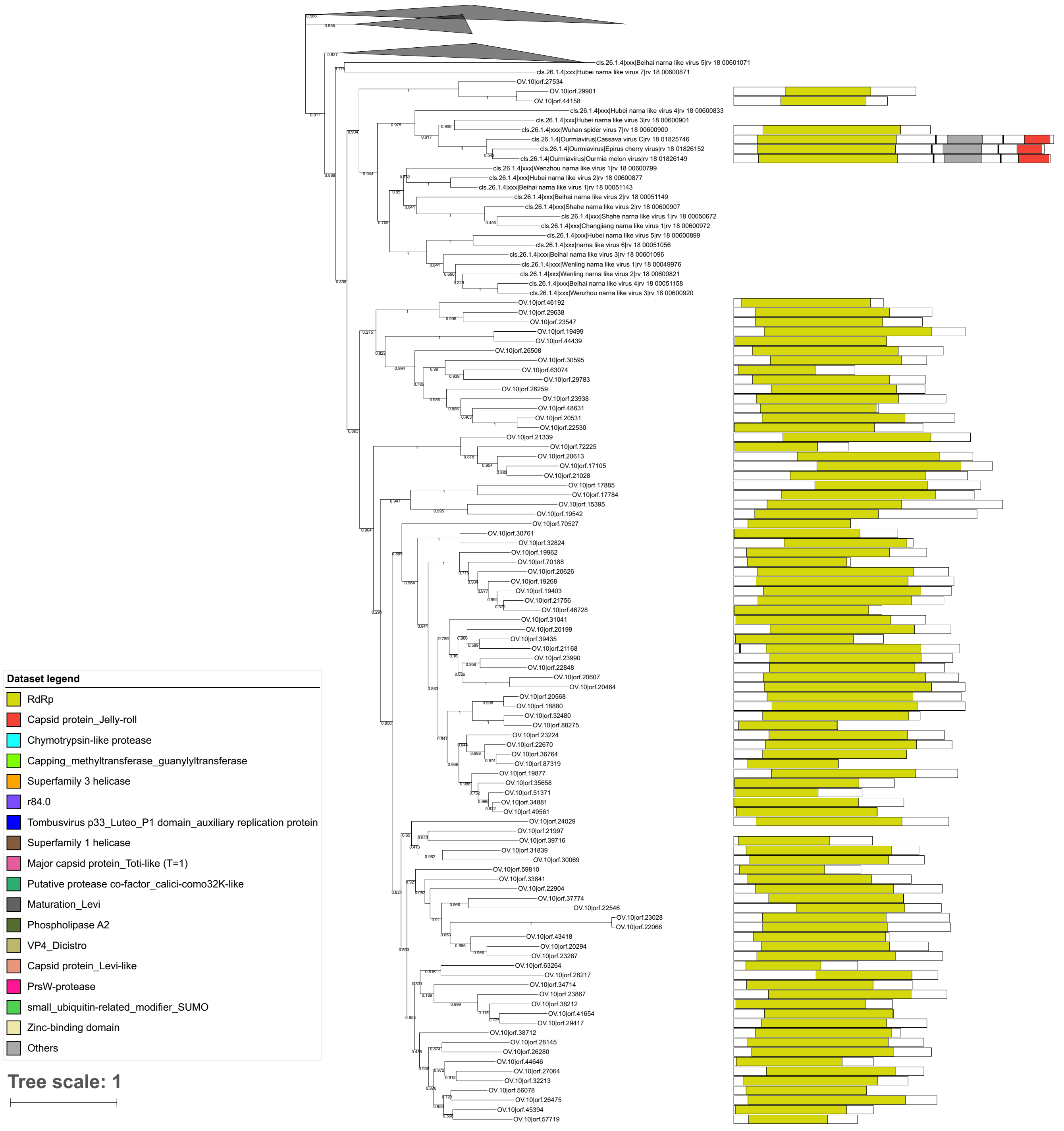

cls.26.1.4|xxx|Beihai narna like virus 5|rv 18 00601071
cls.26.1.4|xxx|Hubei narna like virus 7|rv 18 00600871
OV.10|orf.27534
OV.10|orf.29901
OV.10|orf.44158
cls.26.1.4|xxx|Hubei narna like virus 4|rv 18 00600833
cls.26.1.4|xxx|Hubei narna like virus 3|rv 18 00600901
cls.26.1.4|xxx|Wuhan spider virus 7|rv 18 00600900
cls.26.1.4|Ourmiavirus|Cassava virus C|rv 18 01825746
cls.26.1.4|Ourmiavirus|Epirus cherry virus|rv 18 01826152
cls.26.1.4|Ourmiavirus|Ourmia melon virus|rv 18 01826149
cls.26.1.4|xxx|Wenzhou narna like virus 1|rv 18 00600799
cls.26.1.4|xxx|Hubei narna like virus 2|rv 18 00600877
cls.26.1.4|xxx|Beihai narna like virus 1|rv 18 00051143
cls.26.1.4|xxx|Beihai narna like virus 2|rv 18 00051149
cls.26.1.4|xxx|Shahe narna like virus 2|rv 18 00600907
cls.26.1.4|xxx|Shahe narna like virus 1|rv 18 00050672
cls.26.1.4|xxx|Changjiang narna like virus 1|rv 18 00600972
cls.26.1.4|xxx|Hubei narna like virus 5|rv 18 00600899
cls.26.1.4|xxx|narna like virus 6|rv 18 00051056
cls.26.1.4|xxx|Beihai narna like virus 3|rv 18 00601096
cls.26.1.4|xxx|Wenling narna like virus 1|rv 18 00049976
cls.26.1.4|xxx|Wenling narna like virus 2|rv 18 00600821
cls.26.1.4|xxx|Beihai narna like virus 4|rv 18 00051158
cls.26.1.4|xxx|Wenzhou narna like virus 3|rv 18 00600920
OV.10|orf.46192
OV.10|orf.29638
OV.10|orf.23547
OV.10|orf.19499
OV.10|orf.44439
OV.10|orf.26508
OV.10|orf.30595
OV.10|orf.63074
OV.10|orf.29783
OV.10|orf.26259
OV.10|orf.23938
OV.10|orf.48631
OV.10|orf.20531
OV.10|orf.22530
OV.10|orf.21339
OV.10|orf.72225
OV.10|orf.20613
OV.10|orf.17105
OV.10|orf.21028
OV.10|orf.17885
OV.10|orf.17784
OV.10|orf.15395
OV.10|orf.19542
OV.10|orf.70527
OV.10|orf.30761
OV.10|orf.32824
OV.10|orf.19962
OV.10|orf.70188
OV.10|orf.20626
OV.10|orf.19268
OV.10|orf.19403
OV.10|orf.21756
OV.10|orf.46728
OV.10|orf.31041
OV.10|orf.20199
OV.10|orf.39435
OV.10|orf.21168
OV.10|orf.23990
OV.10|orf.22848
OV.10|orf.20607
OV.10|orf.20464
OV.10|orf.20568
OV.10|orf.18880
OV.10|orf.32480
OV.10|orf.88275
OV.10|orf.23224
OV.10|orf.22670
OV.10|orf.36764
OV.10|orf.87319
OV.10|orf.19877
OV.10|orf.35658
OV.10|orf.51371
OV.10|orf.34881
OV.10|orf.49561
OV.10|orf.24029
OV.10|orf.21997
OV.10|orf.39716
OV.10|orf.31839
OV.10|orf.30069
OV.10|orf.59810
OV.10|orf.33841
OV.10|orf.22904
OV.10|orf.37774
OV.10|orf.22546
OV.10|orf.23028
OV.10|orf.22068
OV.10|orf.43418
OV.10|orf.20294
OV.10|orf.23267
OV.10|orf.63264
OV.10|orf.28217
OV.10|orf.34714
OV.10|orf.23867
OV.10|orf.38212
OV.10|orf.41654
OV.10|orf.29417
OV.10|orf.38712
OV.10|orf.28145
OV.10|orf.26280
OV.10|orf.44646
OV.10|orf.27064
OV.10|orf.32213
OV.10|orf.56078
OV.10|orf.26475
OV.10|orf.45394
OV.10|orf.57719
Dataset legend
RdRp
Capsid protein_Jelly-roll
Chymotrypsin-like protease
Capping_methyltransferase_guanylyltransferase
Superfamily 3 helicase
r84.0
Tombusvirus p33_Luteo_P1 domain_auxiliary replication protein
Superfamily 1 helicase
Major capsid protein_Toti-like (T=1)
Putative protease co-factor_calici-como32K-like
Maturation_Levi
Phospholipase A2
VP4_Dicistro
Capsid protein_Levi-like
PrsW-protease
small_ubiquitin-related_modifier_SUMO
Zinc-binding domain
Others
Tree scale: 1

Ov11

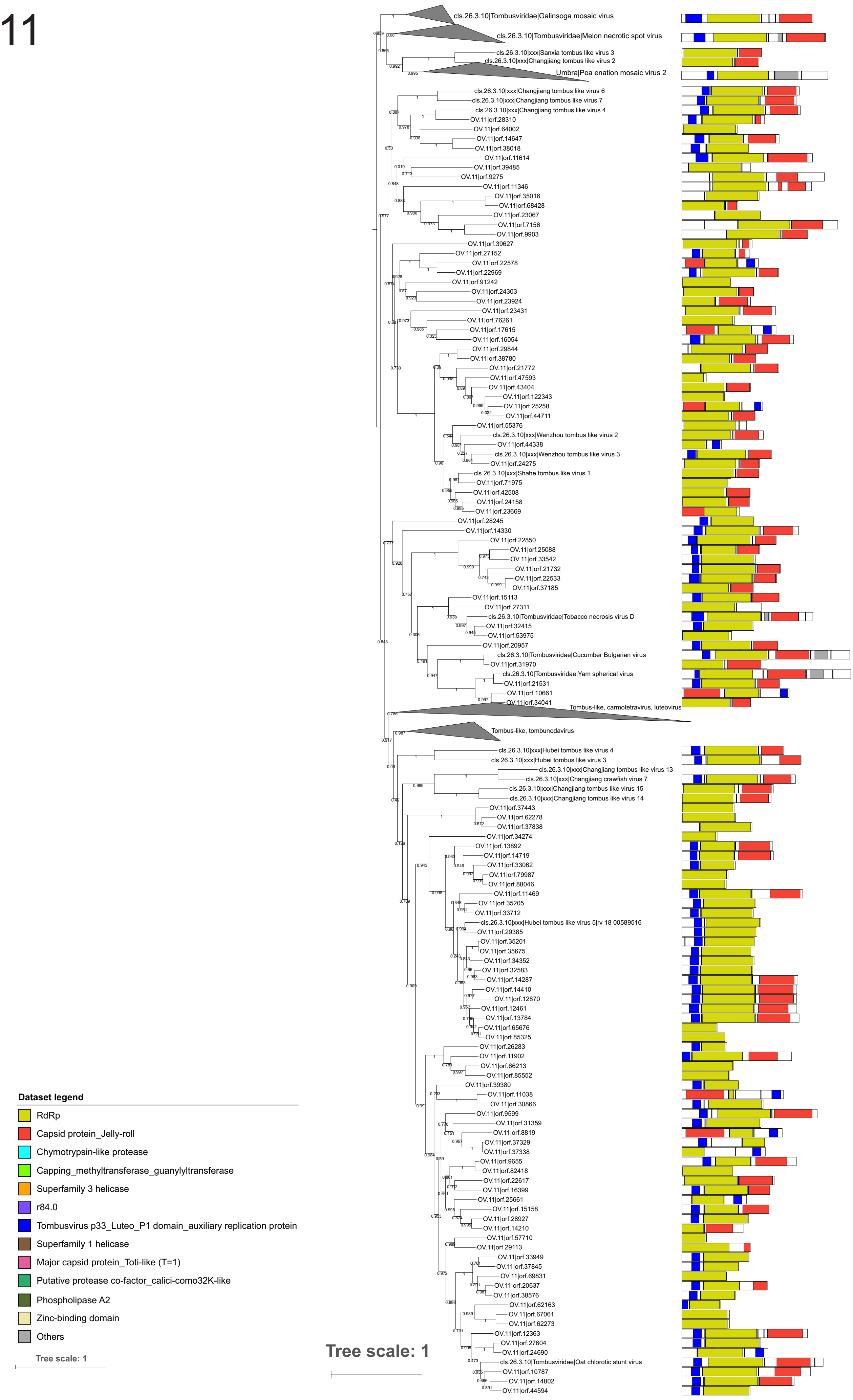
cls.26.3.10|Tombusviridae|Galinsoga mosaic virus
cls.26.3.10|Tombusviridae|Melon necrotic spot virus
cls.26.3.10|xxx|Sanxia tombus like virus 3
cls.26.3.10|xxx|Changjiang tombus like virus 2
Umbra|Pea enation mosaic virus 2
cls.26.3.10|xxx|Changjiang tombus like virus 6
cls.26.3.10|xxx|Changjiang tombus like virus 7
cls.26.3.10|xxx|Changjiang tombus like virus 4
OV.11|orf.28310
OV.11|orf.64002
OV.11|orf.14647
OV.11|orf.38018
OV.11|orf.11614
OV.11|orf.39485
OV.11|orf.9275
OV.11|orf.11346
OV.11|orf.35016
OV.11|orf.68428
OV.11|orf.23067
OV.11|orf.7156
OV.11|orf.9903
OV.11|orf.39627
OV.11|orf.27152
OV.11|orf.22578
OV.11|orf.22969
OV.11|orf.91242
OV.11|orf.24303
OV.11|orf.23924
OV.11|orf.23431
OV.11|orf.76261
OV.11|orf.17615
OV.11|orf.16054
OV.11|orf.29844
OV.11|orf.38780
OV.11|orf.21772
OV.11|orf.47593
OV.11|orf.43404
OV.11|orf.122343
OV.11|orf.25258
OV.11|orf.44711
OV.11|orf.55376
cls.26.3.10|xxx|Wenzhou tombus like virus 2
OV.11|orf.44338
cls.26.3.10|xxx|Wenzhou tombus like virus 3
OV.11|orf.24275
cls.26.3.10|xxx|Shahe tombus like virus 1
OV.11|orf.71975
OV.11|orf.42508
OV.11|orf.24158
OV.11|orf.23669
OV.11|orf.28245
OV.11|orf.14330
OV.11|orf.22850
OV.11|orf.25088
OV.11|orf.33542
OV.11|orf.21732
OV.11|orf.22533
OV.11|orf.37185
OV.11|orf.15113
OV.11|orf.27311
cls.26.3.10|Tombusviridae|Tobacco necrosis virus D
OV.11|orf.32415
OV.11|orf.53975
OV.11|orf.20957
cls.26.3.10|Tombusviridae|Cucumber Bulgarian virus
OV.11|orf.31970
cls.26.3.10|Tombusviridae|Yam spherical virus
OV.11|orf.21531
OV.11|orf.10661
OV.11|orf.34041
Tombus-like, carmotetravirus, luteovirus
Tombus-like, tombunodavirus
cls.26.3.10|xxx|Hubei tombus like virus 4
cls.26.3.10|xxx|Hubei tombus like virus 3
cls.26.3.10|xxx|Changjiang tombus like virus 13
cls.26.3.10|xxx|Changjiang crawfish virus 7
cls.26.3.10|xxx|Changjiang tombus like virus 15
cls.26.3.10|xxx|Changjiang tombus like virus 14
OV.11|orf.37443
OV.11|orf.62278
OV.11|orf.37838
OV.11|orf.34274
OV.11|orf.13892
OV.11|orf.14719
OV.11|orf.33062
OV.11|orf.79987
OV.11|orf.88046
OV.11|orf.11469
OV.11|orf.35205
OV.11|orf.33712
cls.26.3.10|xxx|Hubei tombus like virus 5|rv 18 00589516
OV.11|orf.29385
OV.11|orf.35201
OV.11|orf.35675
OV.11|orf.34352
OV.11|orf.32583
OV.11|orf.14287
OV.11|orf.14410
OV.11|orf.12870
OV.11|orf.12461
OV.11|orf.13784
OV.11|orf.65676
OV.11|orf.85325
OV.11|orf.26283
OV.11|orf.11902
OV.11|orf.66213
OV.11|orf.85552
OV.11|orf.39380
OV.11|orf.11038
OV.11|orf.30866
OV.11|orf.9599
OV.11|orf.31359
OV.11|orf.8819
OV.11|orf.37329
OV.11|orf.37338
OV.11|orf.9655
OV.11|orf.82418
OV.11|orf.22617
OV.11|orf.16399
OV.11|orf.25661
OV.11|orf.15158
OV.11|orf.28927
OV.11|orf.14210
OV.11|orf.57710
OV.11|orf.29113
OV.11|orf.33949
OV.11|orf.37845
OV.11|orf.69831
OV.11|orf.20637
OV.11|orf.38576
OV.11|orf.62163
OV.11|orf.67061
OV.11|orf.62273
OV.11|orf.12363
OV.11|orf.27604
OV.11|orf.24690
cls.26.3.10|Tombusviridae|Oat chlorotic stunt virus
OV.11|orf.10787
OV.11|orf.14802
OV.11|orf.44594
Dataset legend
RdRp
Capsid protein_Jelly-roll
Chymotrypsin-like protease
Capping_methyltransferase_guanylyltransferase
Superfamily 3 helicase
r84.0
Tombusvirus p33_Luteo_P1 domain_auxiliary replication protein
Superfamily 1 helicase
Major capsid protein_Toti-like (T=1)
Putative protease co-factor_calici-como32K-like
Phospholipase A2
Zinc-binding domain
Others
Tree scale: 1
Tree scale: 1

Ov12

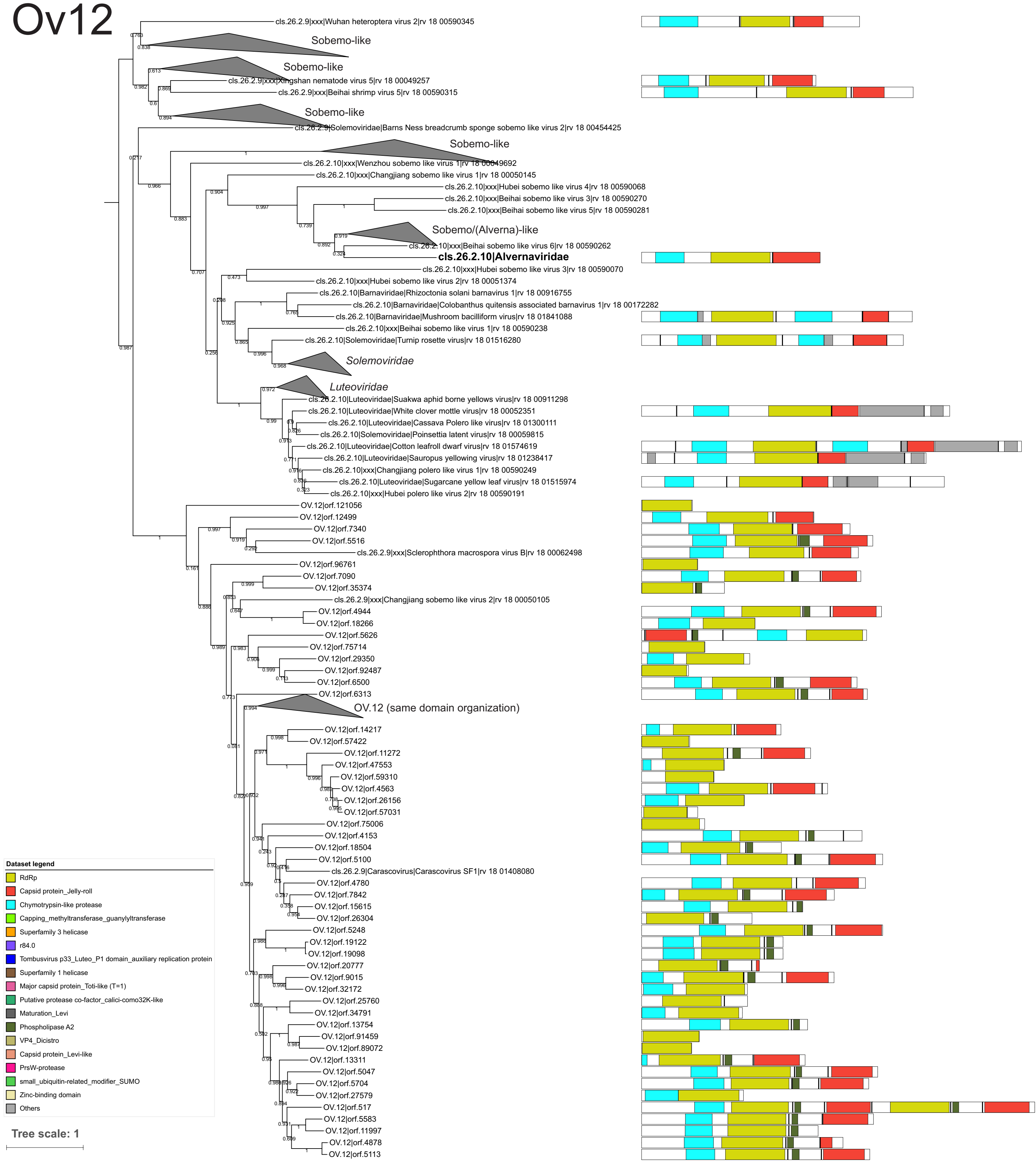

cls.26.2.9|xxx|Wuhan heteroptera virus 2|rv 18 00590345
Sobemo-like
Sobemo-like
cls.26.2.9|xxx|Xingshan nematode virus 5|rv 18 00049257
cls.26.2.9|xxx|Beihai shrimp virus 5|rv 18 00590315
Sobemo-like
cls.26.2.9|Solemoviridae|Barns Ness breadcrumb sponge sobemo like virus 2|rv 18 00454425
Sobemo-like
cls.26.2.10|xxx|Wenzhou sobemo like virus 1|rv 18 00049692
cls.26.2.10|xxx|Changjiang sobemo like virus 1|rv 18 00050145
cls.26.2.10|xxx|Hubei sobemo like virus 4|rv 18 00590068
cls.26.2.10|xxx|Beihai sobemo like virus 3|rv 18 00590270
cls.26.2.10|xxx|Beihai sobemo like virus 5|rv 18 00590281
Sobemo/(Alverna)-like
cls.26.2.10|xxx|Beihai sobemo like virus 6|rv 18 00590262
cls.26.2.10|Alvernaviridae
cls.26.2.10|xxx|Hubei sobemo like virus 3|rv 18 00590070
cls.26.2.10|xxx|Hubei sobemo like virus 2|rv 18 00051374
cls.26.2.10|Barnaviridae|Rhizoctonia solani barnavirus 1|rv 18 00916755
cls.26.2.10|Barnaviridae|Colobanthus quitensis associated barnavirus 1|rv 18 00172282
cls.26.2.10|Barnaviridae|Mushroom bacilliform virus|rv 18 01841088
cls.26.2.10|xxx|Beihai sobemo like virus 1|rv 18 00590238
cls.26.2.10|Solemoviridae|Turnip rosette virus|rv 18 01516280
Solemoviridae
Luteoviridae
cls.26.2.10|Luteoviridae|Suakwa aphid borne yellows virus|rv 18 00911298
cls.26.2.10|Luteoviridae|White clover mottle virus|rv 18 00052351
cls.26.2.10|Luteoviridae|Cassava Polero like virus|rv 18 01300111
cls.26.2.10|Solemoviridae|Poinsettia latent virus|rv 18 00059815
cls.26.2.10|Luteoviridae|Cotton leafroll dwarf virus|rv 18 01574619
cls.26.2.10|Luteoviridae|Sauropus yellowing virus|rv 18 01238417
cls.26.2.10|xxx|Changjiang polero like virus 1|rv 18 00590249
cls.26.2.10|Luteoviridae|Sugarcane yellow leaf virus|rv 18 01515974
cls.26.2.10|xxx|Hubei polero like virus 2|rv 18 00590191
OV.12|orf.121056
OV.12|orf.12499
OV.12|orf.7340
OV.12|orf.5516
cls.26.2.9|xxx|Sclerophthora macrospora virus B|rv 18 00062498
OV.12|orf.96761
OV.12|orf.7090
OV.12|orf.35374
cls.26.2.9|xxx|Changjiang sobemo like virus 2|rv 18 00050105
OV.12|orf.4944
OV.12|orf.18266
OV.12|orf.5626
OV.12|orf.75714
OV.12|orf.29350
OV.12|orf.92487
OV.12|orf.6500
OV.12|orf.6313
OV.12 (same domain organization)
OV.12|orf.14217
OV.12|orf.57422
OV.12|orf.11272
OV.12|orf.47553
OV.12|orf.59310
OV.12|orf.4563
OV.12|orf.26156
OV.12|orf.57031
OV.12|orf.75006
OV.12|orf.4153
OV.12|orf.18504
OV.12|orf.5100
cls.26.2.9|Carascovirus|Carascovirus SF1|rv 18 01408080
OV.12|orf.4780
OV.12|orf.7842
OV.12|orf.15615
OV.12|orf.26304
OV.12|orf.5248
OV.12|orf.19122
OV.12|orf.19098
OV.12|orf.20777
OV.12|orf.9015
OV.12|orf.32172
OV.12|orf.25760
OV.12|orf.34791
OV.12|orf.13754
OV.12|orf.91459
OV.12|orf.89072
OV.12|orf.13311
OV.12|orf.5047
OV.12|orf.5704
OV.12|orf.27579
OV.12|orf.517
OV.12|orf.5583
OV.12|orf.11997
OV.12|orf.4878
OV.12|orf.5113
Dataset legend
RdRp
Capsid protein_Jelly-roll
Chymotrypsin-like protease
Capping_methyltransferase_guanylyltransferase
Superfamily 3 helicase
r84.0
Tombusvirus p33_Luteo_P1 domain_auxiliary replication protein
Superfamily 1 helicase
Major capsid protein_Toti-like (T=1)
Putative protease co-factor_calici-como32K-like
Maturation_Levi
Phospholipase A2
VP4_Dicistro
Capsid protein_Levi-like
PrsW-protease
small_ubiquitin-related_modifier_SUMO
Zinc-binding domain
Others
Tree scale: 1

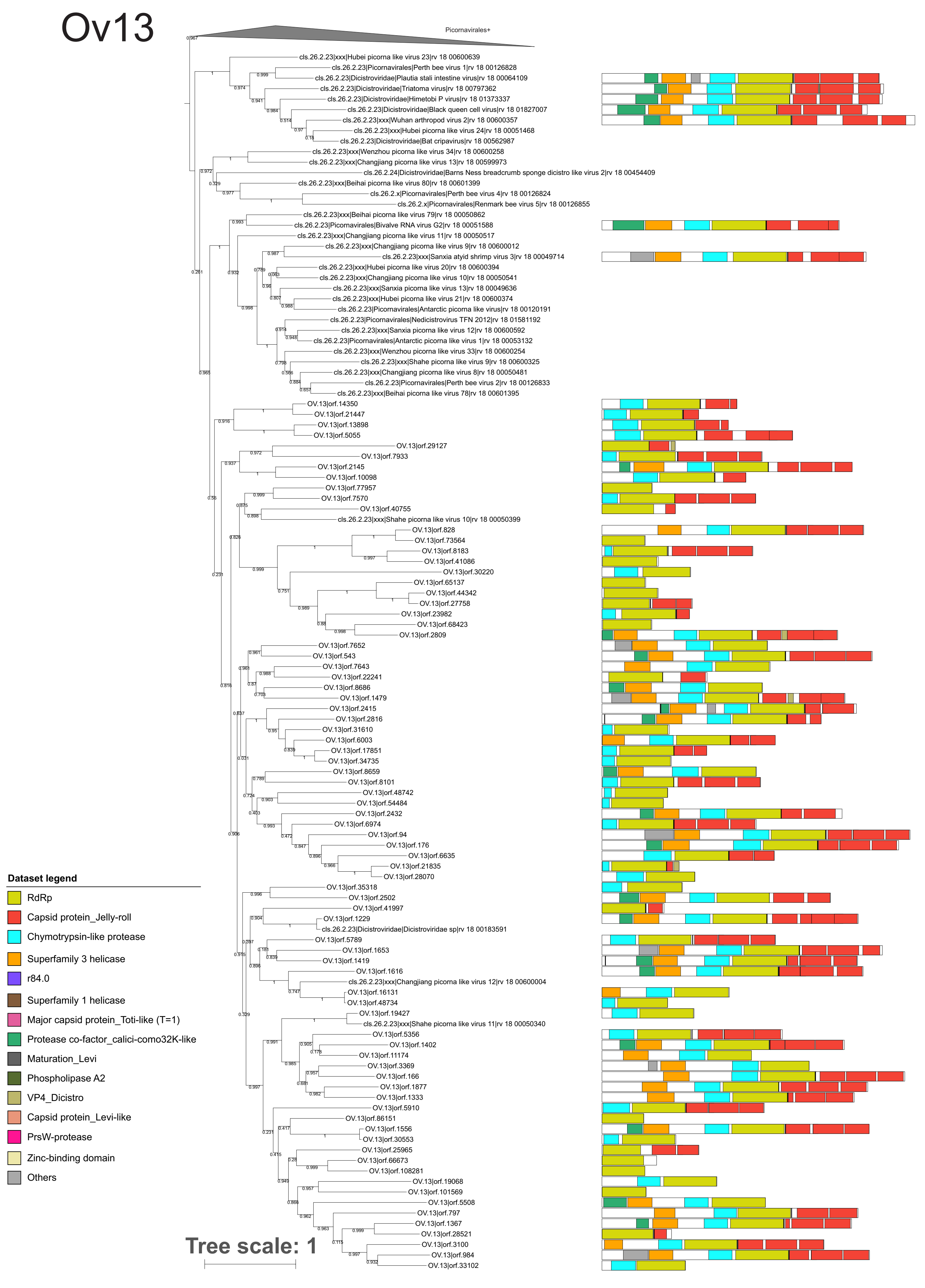
Ov13
Picornavirales+
cls.26.2.23|xxx|Hubei picorna like virus 23|rv 18 00600639
cls.26.2.23|Picornavirales|Perth bee virus 1|rv 18 00126828
cls.26.2.23|Dicistroviridae|Plautia stali intestine virus|rv 18 00064109
cls.26.2.23|Dicistroviridae|Triatoma virus|rv 18 00797362
cls.26.2.23|Dicistroviridae|Himetobi P virus|rv 18 01373337
cls.26.2.23|Dicistroviridae|Black queen cell virus|rv 18 01827007
cls.26.2.23|xxx|Wuhan arthropod virus 2|rv 18 00600357
cls.26.2.23|xxx|Hubei picorna like virus 24|rv 18 00051468
cls.26.2.23|Dicistroviridae|Bat cripavirus|rv 18 00562987
cls.26.2.23|xxx|Wenzhou picorna like virus 34|rv 18 00600258
cls.26.2.23|xxx|Changjiang picorna like virus 13|rv 18 00599973
cls.26.2.24|Dicistroviridae|Barns Ness breadcrumb sponge dicistro like virus 2|rv 18 00454409
cls.26.2.23|xxx|Beihai picorna like virus 80|rv 18 00601399
cls.26.2.x|Picornavirales|Perth bee virus 4|rv 18 00126824
cls.26.2.x|Picornavirales|Renmark bee virus 5|rv 18 00126855
cls.26.2.23|xxx|Beihai picorna like virus 79|rv 18 00050862
cls.26.2.23|Picornavirales|Bivalve RNA virus G2|rv 18 00051588
cls.26.2.23|xxx|Changjiang picorna like virus 11|rv 18 00050517
cls.26.2.23|xxx|Changjiang picorna like virus 9|rv 18 00600012
cls.26.2.23|xxx|Sanxia atyid shrimp virus 3|rv 18 00049714
cls.26.2.23|xxx|Hubei picorna like virus 20|rv 18 00600394
cls.26.2.23|xxx|Changjiang picorna like virus 10|rv 18 00050541
cls.26.2.23|xxx|Sanxia picorna like virus 13|rv 18 00049636
cls.26.2.23|xxx|Hubei picorna like virus 21|rv 18 00600374
cls.26.2.23|Picornavirales|Antarctic picorna like virus|rv 18 00120191
cls.26.2.23|Picornavirales|Nedicistrovirus TFN 2012|rv 18 01581192
cls.26.2.23|xxx|Sanxia picorna like virus 12|rv 18 00600592
cls.26.2.23|Picornavirales|Antarctic picorna like virus 1|rv 18 00053132
cls.26.2.23|xxx|Wenzhou picorna like virus 33|rv 18 00600254
cls.26.2.23|xxx|Shahe picorna like virus 9|rv 18 00600325
cls.26.2.23|xxx|Changjiang picorna like virus 8|rv 18 00050481
cls.26.2.23|Picornavirales|Perth bee virus 2|rv 18 00126833
cls.26.2.23|xxx|Beihai picorna like virus 78|rv 18 00601395
OV.13|orf.14350
OV.13|orf.21447
OV.13|orf.13898
OV.13|orf.5055
OV.13|orf.29127
OV.13|orf.7933
OV.13|orf.2145
OV.13|orf.10098
OV.13|orf.77957
OV.13|orf.7570
OV.13|orf.40755
cls.26.2.23|xxx|Shahe picorna like virus 10|rv 18 00050399
OV.13|orf.828
OV.13|orf.73564
OV.13|orf.8183
OV.13|orf.41086
OV.13|orf.30220
OV.13|orf.65137
OV.13|orf.44342
OV.13|orf.27758
OV.13|orf.23982
OV.13|orf.68423
OV.13|orf.2809
OV.13|orf.7652
OV.13|orf.543
OV.13|orf.7643
OV.13|orf.22241
OV.13|orf.8686
OV.13|orf.1479
OV.13|orf.2415
OV.13|orf.2816
OV.13|orf.31610
OV.13|orf.6003
OV.13|orf.17851
OV.13|orf.34735
OV.13|orf.8659
OV.13|orf.8101
OV.13|orf.48742
OV.13|orf.54484
OV.13|orf.2432
OV.13|orf.6974
OV.13|orf.94
OV.13|orf.176
OV.13|orf.6635
OV.13|orf.21835
OV.13|orf.28070
OV.13|orf.35318
OV.13|orf.2502
OV.13|orf.41997
OV.13|orf.1229
cls.26.2.23|Dicistroviridae|Dicistroviridae sp|rv 18 00183591
OV.13|orf.5789
OV.13|orf.1653
OV.13|orf.1419
OV.13|orf.1616
cls.26.2.23|xxx|Changjiang picorna like virus 12|rv 18 00600004
OV.13|orf.16131
OV.13|orf.48734
OV.13|orf.19427
cls.26.2.23|xxx|Shahe picorna like virus 11|rv 18 00050340
OV.13|orf.5356
OV.13|orf.1402
OV.13|orf.11174
OV.13|orf.3369
OV.13|orf.166
OV.13|orf.1877
OV.13|orf.1333
OV.13|orf.5910
OV.13|orf.86151
OV.13|orf.1556
OV.13|orf.30553
OV.13|orf.25965
OV.13|orf.66673
OV.13|orf.108281
OV.13|orf.19068
OV.13|orf.101569
OV.13|orf.5508
OV.13|orf.797
OV.13|orf.1367
OV.13|orf.28521
OV.13|orf.3100
OV.13|orf.984
OV.13|orf.33102
Dataset legend
RdRp
Capsid protein_Jelly-roll
Chymotrypsin-like protease
Superfamily 3 helicase
r84.0
Superfamily 1 helicase
Major capsid protein_Toti-like (T=1)
Protease co-factor_calici-como32K-like
Maturation_Levi
Phospholipase A2
VP4_Dicistro
Capsid protein_Levi-like
PrsW-protease
Zinc-binding domain
Others
Tree scale: 1

Ov16

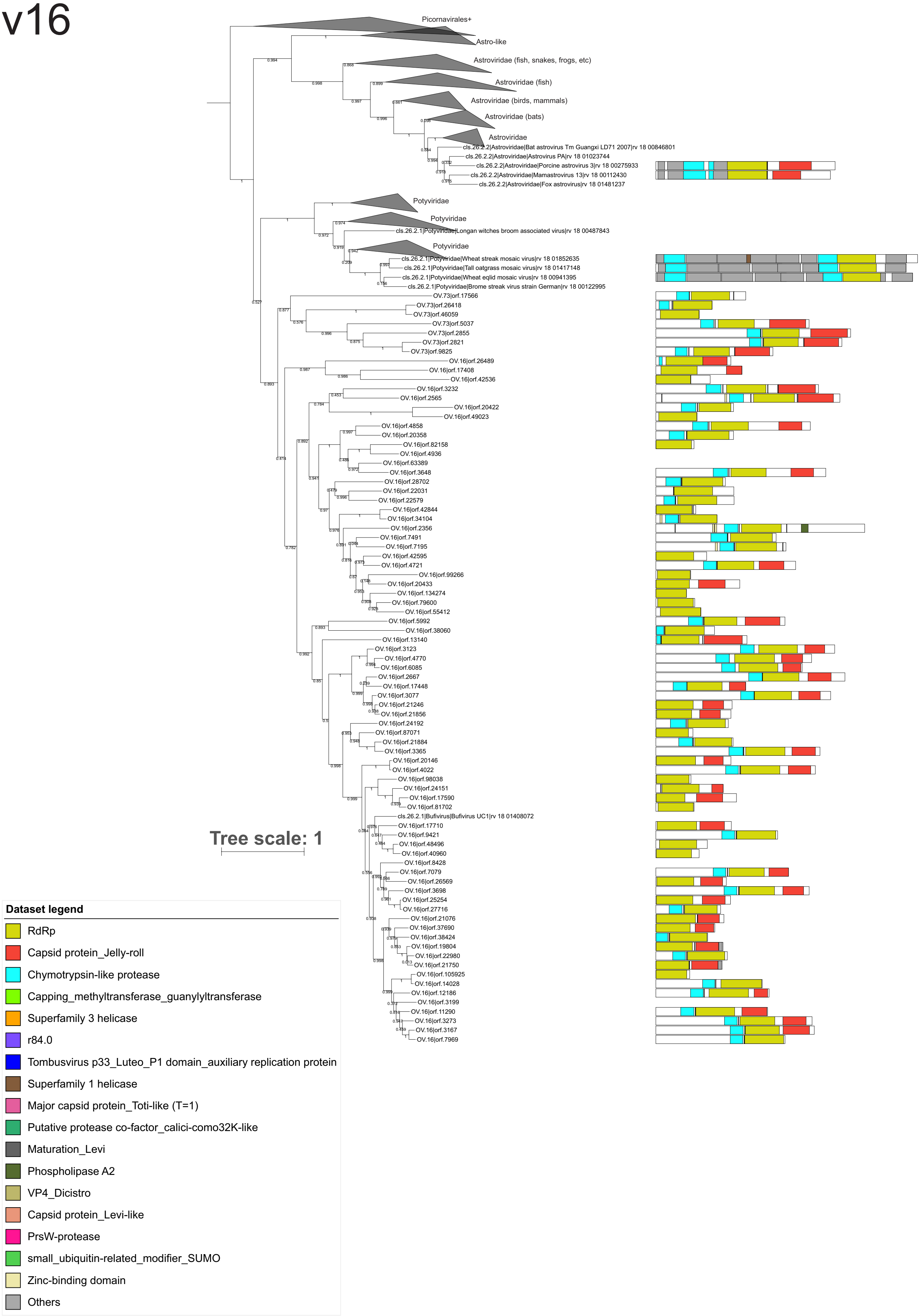

Picornavirales+
Astro-like
Astroviridae (fish, snakes, frogs, etc)
Astroviridae (fish)
Astroviridae (birds, mammals)
Astroviridae (bats)
Astroviridae
cls.26.2.2|Astroviridae|Bat astrovirus Tm Guangxi LD71 2007|rv 18 00846801
cls.26.2.2|Astroviridae|Astrovirus PA|rv 18 01023744
cls.26.2.2|Astroviridae|Porcine astrovirus 3|rv 18 00275933
cls.26.2.2|Astroviridae|Mamastrovirus 13|rv 18 00112430
cls.26.2.2|Astroviridae|Fox astrovirus|rv 18 01481237
Potyviridae
Potyviridae
cls.26.2.1|Potyviridae|Longan witches broom associated virus|rv 18 00487843
Potyviridae
cls.26.2.1|Potyviridae|Wheat streak mosaic virus|rv 18 01852635
cls.26.2.1|Potyviridae|Tall oatgrass mosaic virus|rv 18 01417148
cls.26.2.1|Potyviridae|Wheat eqlid mosaic virus|rv 18 00941395
cls.26.2.1|Potyviridae|Brome streak virus strain German|rv 18 00122995
OV.73|orf.17566
OV.73|orf.26418
OV.73|orf.46059
OV.73|orf.5037
OV.73|orf.2855
OV.73|orf.2821
OV.73|orf.9825
OV.16|orf.26489
OV.16|orf.17408
OV.16|orf.42536
OV.16|orf.3232
OV.16|orf.2565
OV.16|orf.20422
OV.16|orf.49023
OV.16|orf.4858
OV.16|orf.20358
OV.16|orf.82158
OV.16|orf.4936
OV.16|orf.63389
OV.16|orf.3648
OV.16|orf.28702
OV.16|orf.22031
OV.16|orf.22579
OV.16|orf.42844
OV.16|orf.34104
OV.16|orf.2356
OV.16|orf.7491
OV.16|orf.7195
OV.16|orf.42595
OV.16|orf.4721
OV.16|orf.99266
OV.16|orf.20433
OV.16|orf.134274
OV.16|orf.79600
OV.16|orf.55412
OV.16|orf.5992
OV.16|orf.38060
OV.16|orf.13140
OV.16|orf.3123
OV.16|orf.4770
OV.16|orf.6085
OV.16|orf.2667
OV.16|orf.17448
OV.16|orf.3077
OV.16|orf.21246
OV.16|orf.21856
OV.16|orf.24192
OV.16|orf.87071
OV.16|orf.21884
OV.16|orf.3365
OV.16|orf.20146
OV.16|orf.4022
OV.16|orf.98038
OV.16|orf.24151
OV.16|orf.17590
OV.16|orf.81702
cls.26.2.1|Bufivirus|Bufivirus UC1|rv 18 01408072
OV.16|orf.17710
OV.16|orf.9421
OV.16|orf.48496
OV.16|orf.40960
OV.16|orf.8428
OV.16|orf.7079
OV.16|orf.26569
OV.16|orf.3698
OV.16|orf.25254
OV.16|orf.27716
OV.16|orf.21076
OV.16|orf.37690
OV.16|orf.38424
OV.16|orf.19804
OV.16|orf.22980
OV.16|orf.21750
OV.16|orf.105925
OV.16|orf.14028
OV.16|orf.12186
OV.16|orf.3199
OV.16|orf.11290
OV.16|orf.3273
OV.16|orf.3167
OV.16|orf.7969
Tree scale: 1
Dataset legend
RdRp
Capsid protein_Jelly-roll
Chymotrypsin-like protease
Capping_methyltransferase_guanylyltransferase
Superfamily 3 helicase
r84.0
Tombusvirus p33_Luteo_P1 domain_auxiliary replication protein
Superfamily 1 helicase
Major capsid protein_Toti-like (T=1)
Putative protease co-factor_calici-como32K-like
Maturation_Levi
Phospholipase A2
VP4_Dicistro
Capsid protein_Levi-like
PrsW-protease
small_ubiquitin-related_modifier_SUMO
Zinc-binding domain
Others

Ov23

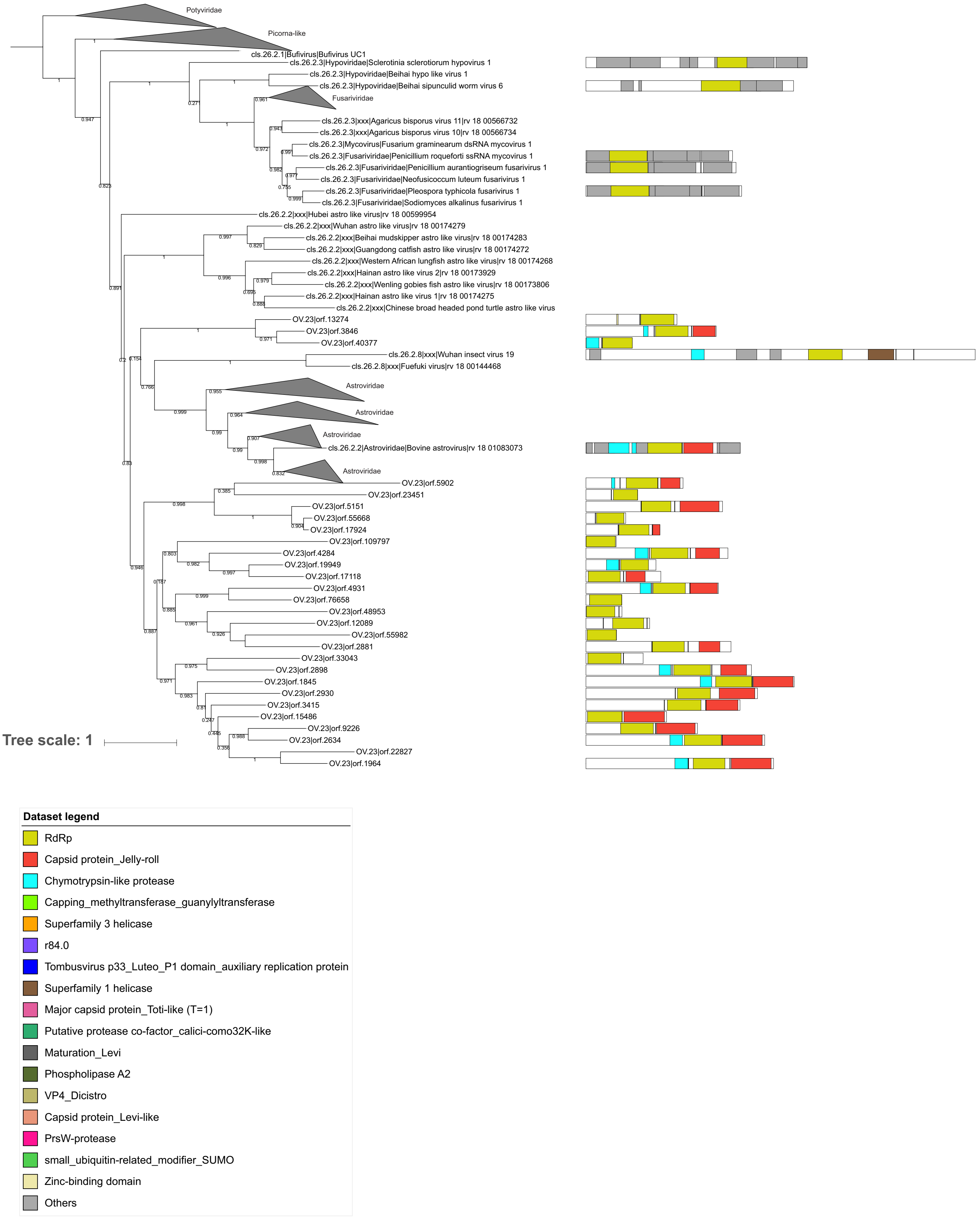
Potyviridae
Picorna-like
cls.26.2.1|Bufivirus|Bufivirus UC1
cls.26.2.3|Hypoviridae|Sclerotinia sclerotiorum hypovirus 1
cls.26.2.3|Hypoviridae|Beihai hypo like virus 1
cls.26.2.3|Hypoviridae|Beihai sipunculid worm virus 6
Fusariviridae
cls.26.2.3|xxx|Agaricus bisporus virus 11|rv 18 00566732
cls.26.2.3|xxx|Agaricus bisporus virus 10|rv 18 00566734
cls.26.2.3|Mycovirus|Fusarium graminearum dsRNA mycovirus 1
cls.26.2.3|Fusariviridae|Penicillium roqueforti ssRNA mycovirus 1
cls.26.2.3|Fusariviridae|Penicillium aurantiogriseum fusarivirus 1
cls.26.2.3|Fusariviridae|Neofusicoccum luteum fusarivirus 1
cls.26.2.3|Fusariviridae|Pleospora typhicola fusarivirus 1
cls.26.2.3|Fusariviridae|Sodiomyces alkalinus fusarivirus 1
cls.26.2.2|xxx|Hubei astro like virus|rv 18 00599954
cls.26.2.2|xxx|Wuhan astro like virus|rv 18 00174279
cls.26.2.2|xxx|Beihai mudskipper astro like virus|rv 18 00174283
cls.26.2.2|xxx|Guangdong catfish astro like virus|rv 18 00174272
cls.26.2.2|xxx|Western African lungfish astro like virus|rv 18 00174268
cls.26.2.2|xxx|Hainan astro like virus 2|rv 18 00173929
cls.26.2.2|xxx|Wenling gobies fish astro like virus|rv 18 00173806
cls.26.2.2|xxx|Hainan astro like virus 1|rv 18 00174275
cls.26.2.2|xxx|Chinese broad headed pond turtle astro like virus
OV.23|orf.13274
OV.23|orf.3846
OV.23|orf.40377
cls.26.2.8|xxx|Wuhan insect virus 19
cls.26.2.8|xxx|Fuefuki virus|rv 18 00144468
Astroviridae
Astroviridae
Astroviridae
cls.26.2.2|Astroviridae|Bovine astrovirus|rv 18 01083073
Astroviridae
OV.23|orf.5902
OV.23|orf.23451
OV.23|orf.5151
OV.23|orf.55668
OV.23|orf.17924
OV.23|orf.109797
OV.23|orf.4284
OV.23|orf.19949
OV.23|orf.17118
OV.23|orf.4931
OV.23|orf.76658
OV.23|orf.48953
OV.23|orf.12089
OV.23|orf.55982
OV.23|orf.2881
OV.23|orf.33043
OV.23|orf.2898
OV.23|orf.1845
OV.23|orf.2930
OV.23|orf.3415
OV.23|orf.15486
OV.23|orf.9226
OV.23|orf.2634
OV.23|orf.22827
OV.23|orf.1964
Tree scale: 1
Dataset legend
RdRp
Capsid protein_Jelly-roll
Chymotrypsin-like protease
Capping_methyltransferase_guanylyltransferase
Superfamily 3 helicase
r84.0
Tombusvirus p33_Luteo_P1 domain_auxiliary replication protein
Superfamily 1 helicase
Major capsid protein_Toti-like (T=1)
Putative protease co-factor_calici-como32K-like
Maturation_Levi
Phospholipase A2
VP4_Dicistro
Capsid protein_Levi-like
PrsW-protease
small_ubiquitin-related_modifier_SUMO
Zinc-binding domain
Others

Ov29

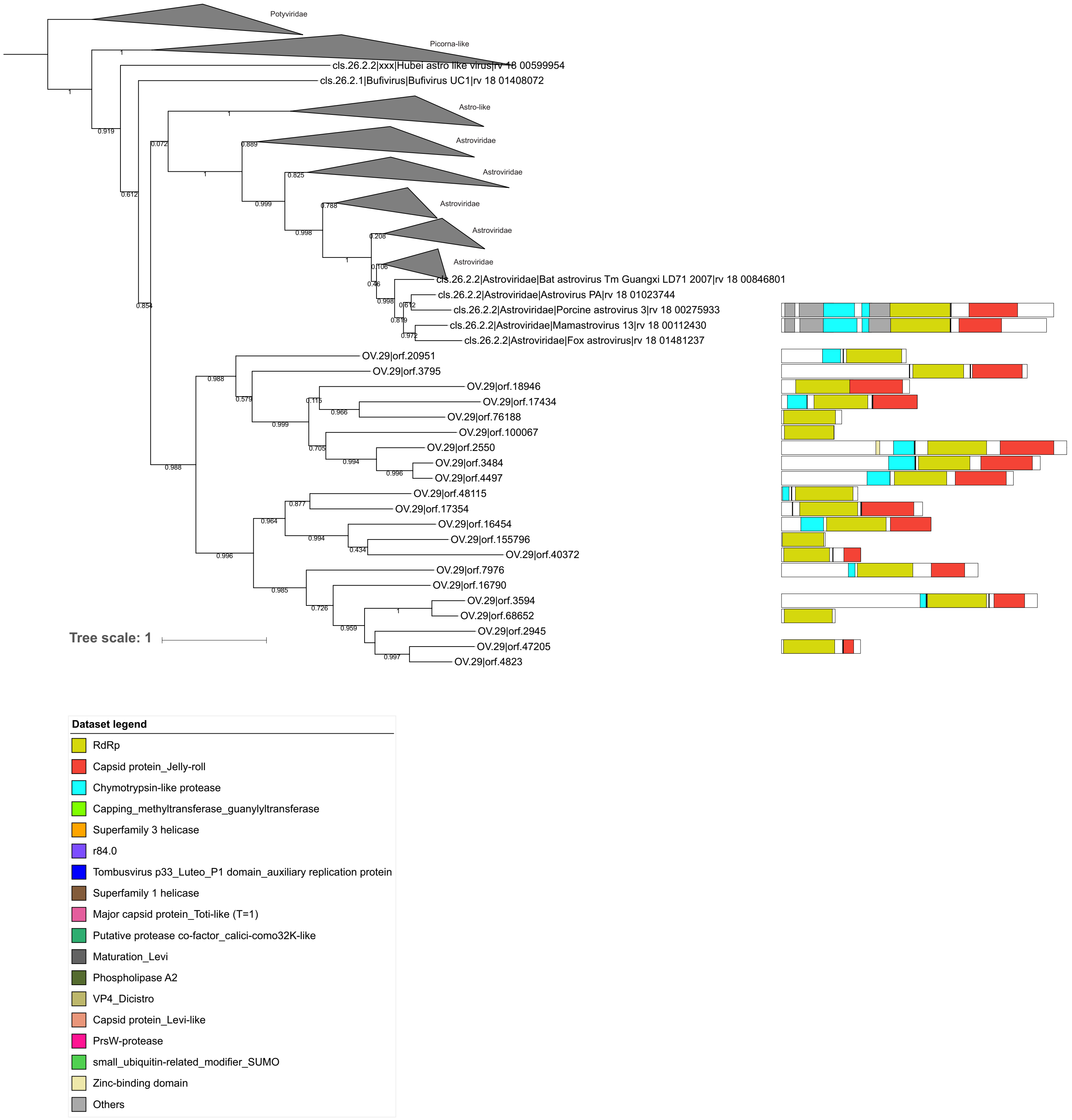

Potyviridae
Picorna-like
cls.26.2.2|xxx|Hubei astro like virus|rv 18 00599954
cls.26.2.1|Bufivirus|Bufivirus UC1|rv 18 01408072
Astro-like
Astroviridae
Astroviridae
Astroviridae
Astroviridae
Astroviridae
cls.26.2.2|Astroviridae|Bat astrovirus Tm Guangxi LD71 2007|rv 18 00846801
cls.26.2.2|Astroviridae|Astrovirus PA|rv 18 01023744
cls.26.2.2|Astroviridae|Porcine astrovirus 3|rv 18 00275933
cls.26.2.2|Astroviridae|Mamastrovirus 13|rv 18 00112430
cls.26.2.2|Astroviridae|Fox astrovirus|rv 18 01481237
OV.29|orf.20951
OV.29|orf.3795
OV.29|orf.18946
OV.29|orf.17434
OV.29|orf.76188
OV.29|orf.100067
OV.29|orf.2550
OV.29|orf.3484
OV.29|orf.4497
OV.29|orf.48115
OV.29|orf.17354
OV.29|orf.16454
OV.29|orf.155796
OV.29|orf.40372
OV.29|orf.7976
OV.29|orf.16790
OV.29|orf.3594
OV.29|orf.68652
OV.29|orf.2945
OV.29|orf.47205
OV.29|orf.4823
1
1
0.919
0.612
0.072
0.854
1
0.889
1
0.999
0.825
0.998
0.788
1
0.208
0.106
0.46
0.998
0.612
0.819
0.972
0.988
0.988
0.579
0.999
0.115
0.966
0.705
0.994
0.996
0.877
0.964
0.994
0.434
0.996
0.985
0.726
1
0.959
0.997
Tree scale: 1
Dataset legend
RdRp
Capsid protein_Jelly-roll
Chymotrypsin-like protease
Capping_methyltransferase_guanylyltransferase
Superfamily 3 helicase
r84.0
Tombusvirus p33_Luteo_P1 domain_auxiliary replication protein
Superfamily 1 helicase
Major capsid protein_Toti-like (T=1)
Putative protease co-factor_calici-como32K-like
Maturation_Levi
Phospholipase A2
VP4_Dicistro
Capsid protein_Levi-like
PrsW-protease
small_ubiquitin-related_modifier_SUMO
Zinc-binding domain
Others

Ov34

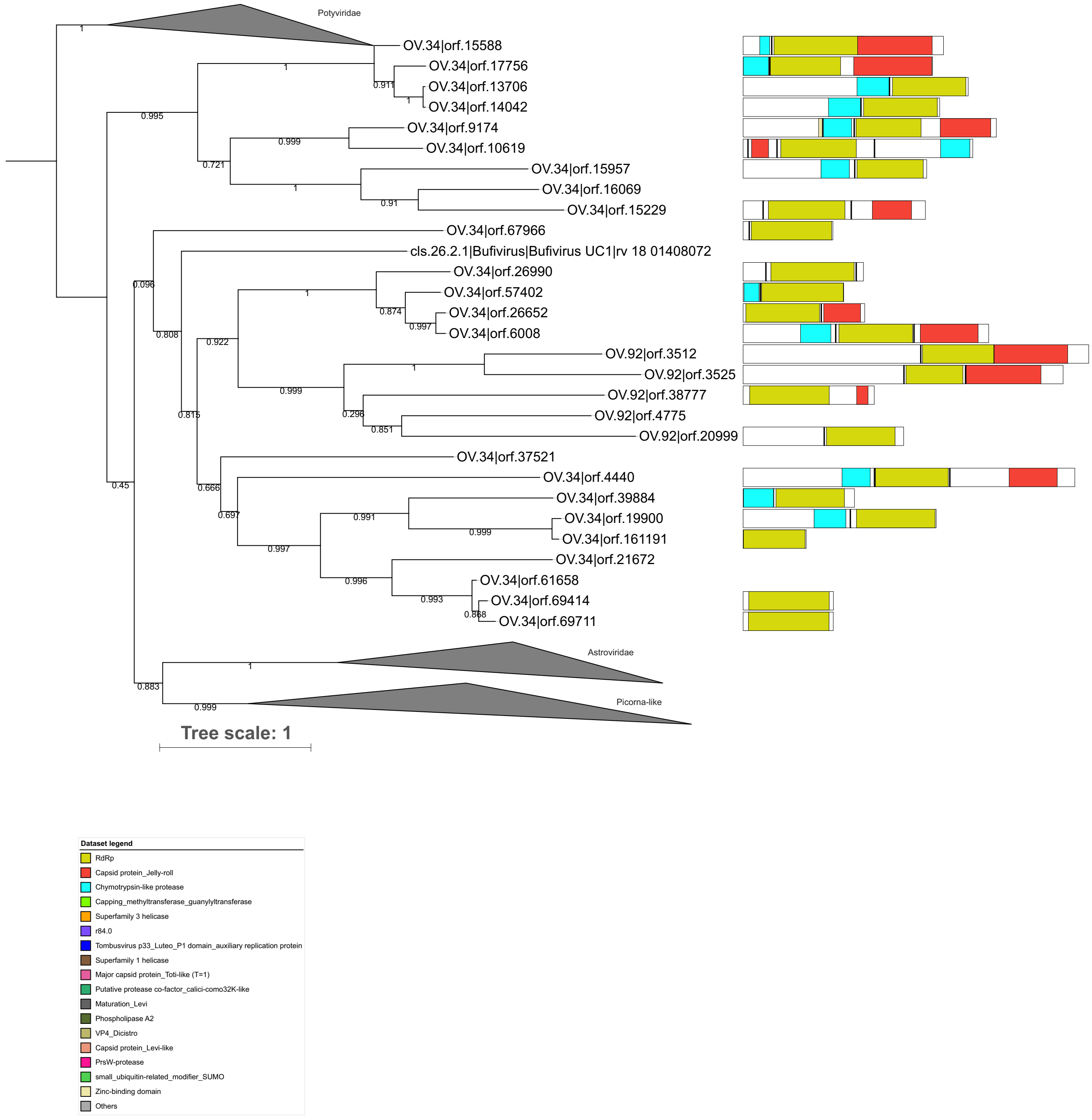

Potyviridae
OV.34|orf.15588
OV.34|orf.17756
OV.34|orf.13706
OV.34|orf.14042
OV.34|orf.9174
OV.34|orf.10619
OV.34|orf.15957
OV.34|orf.16069
OV.34|orf.15229
OV.34|orf.67966
cls.26.2.1|Bufivirus|Bufivirus UC1|rv 18 01408072
OV.34|orf.26990
OV.34|orf.57402
OV.34|orf.26652
OV.34|orf.6008
OV.92|orf.3512
OV.92|orf.3525
OV.92|orf.38777
OV.92|orf.4775
OV.92|orf.20999
OV.34|orf.37521
OV.34|orf.4440
OV.34|orf.39884
OV.34|orf.19900
OV.34|orf.161191
OV.34|orf.21672
OV.34|orf.61658
OV.34|orf.69414
OV.34|orf.69711
Astroviridae
Picorna-like
Tree scale: 1
Dataset legend
RdRp
Capsid protein_Jelly-roll
Chymotrypsin-like protease
Capping_methyltransferase_guanylyltransferase
Superfamily 3 helicase
r84.0
Tombusvirus p33_Luteo_P1 domain_auxiliary replication protein
Superfamily 1 helicase
Major capsid protein_Toti-like (T=1)
Putative protease co-factor_calici-como32K-like
Maturation_Levi
Phospholipase A2
VP4_Dicistro
Capsid protein_Levi-like
PrsW-protease
small_ubiquitin-related_modifier_SUMO
Zinc-binding domain
Others

Ov38

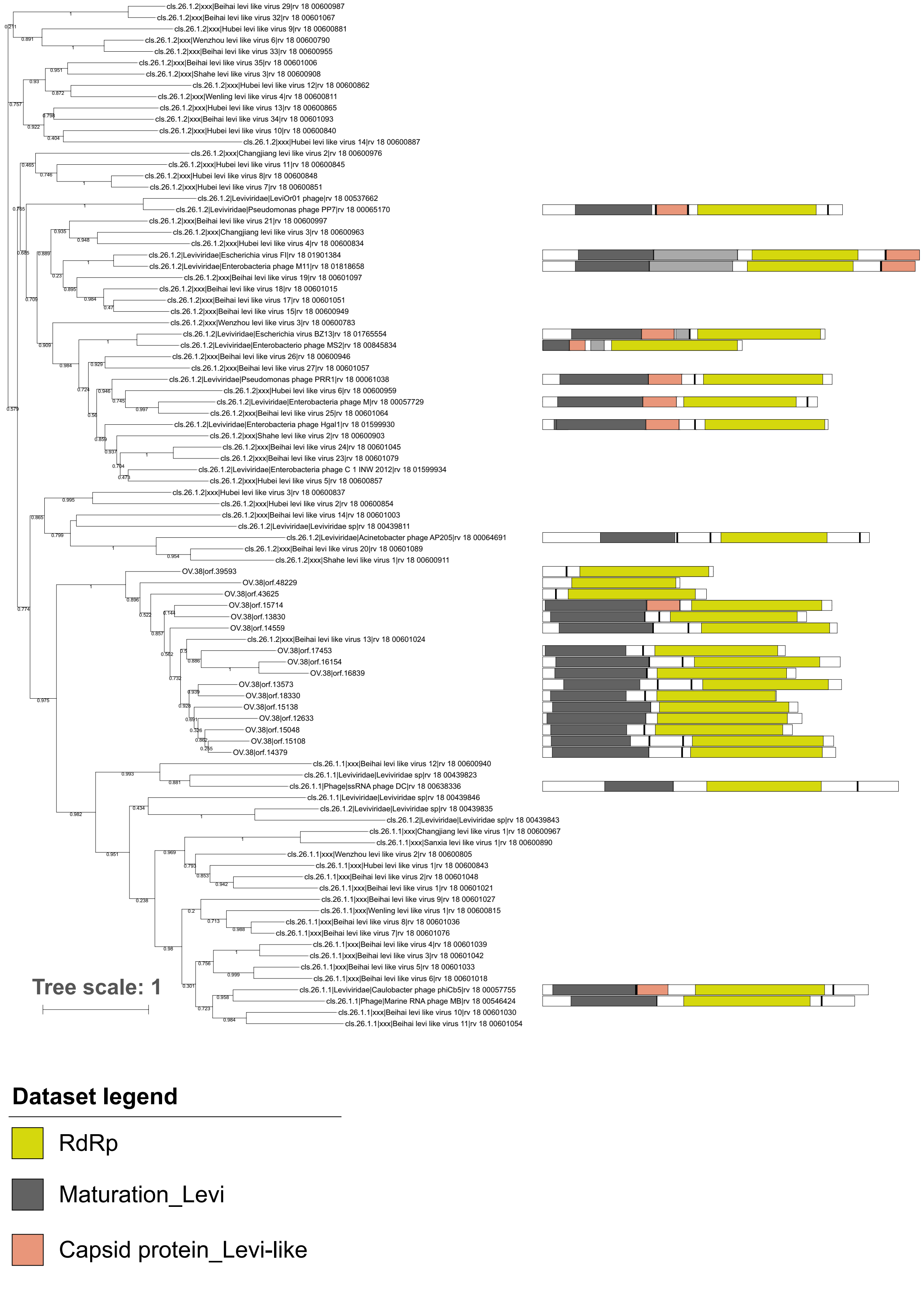

Tree scale: 1
Dataset legend
RdRp
Maturation_Levi
Capsid protein_Levi-like

Ov50

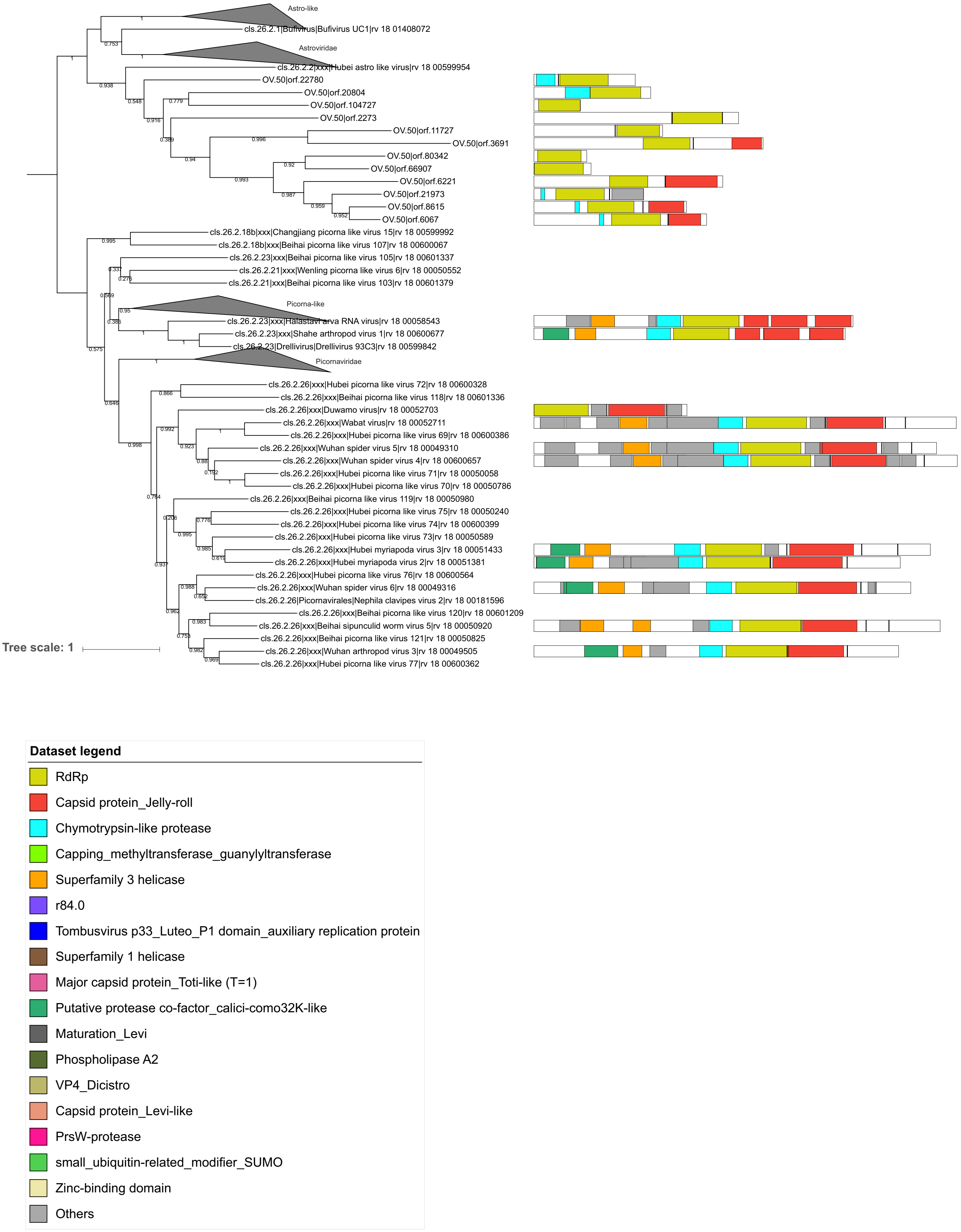
Astro-like
cls.26.2.1|Bufivirus|Bufivirus UC1|rv 18 01408072
Astroviridae
cls.26.2.2|xxx|Hubei astro like virus|rv 18 00599954
OV.50|orf.22780
OV.50|orf.20804
OV.50|orf.104727
OV.50|orf.2273
OV.50|orf.11727
OV.50|orf.3691
OV.50|orf.80342
OV.50|orf.66907
OV.50|orf.6221
OV.50|orf.21973
OV.50|orf.8615
OV.50|orf.6067
cls.26.2.18b|xxx|Changjiang picorna like virus 15|rv 18 00599992
cls.26.2.18b|xxx|Beihai picorna like virus 107|rv 18 00600067
cls.26.2.23|xxx|Beihai picorna like virus 105|rv 18 00601337
cls.26.2.21|xxx|Wenling picorna like virus 6|rv 18 00050552
cls.26.2.21|xxx|Beihai picorna like virus 103|rv 18 00601379
Picorna-like
cls.26.2.23|xxx|Halastavi arva RNA virus|rv 18 00058543
cls.26.2.23|xxx|Shahe arthropod virus 1|rv 18 00600677
cls.26.2.23|Drellivirus|Drellivirus 93C3|rv 18 00599842
Picornaviridae
cls.26.2.26|xxx|Hubei picorna like virus 72|rv 18 00600328
cls.26.2.26|xxx|Beihai picorna like virus 118|rv 18 00601336
cls.26.2.26|xxx|Duwamo virus|rv 18 00052703
cls.26.2.26|xxx|Wabat virus|rv 18 00052711
cls.26.2.26|xxx|Hubei picorna like virus 69|rv 18 00600386
cls.26.2.26|xxx|Wuhan spider virus 5|rv 18 00049310
cls.26.2.26|xxx|Wuhan spider virus 4|rv 18 00600657
cls.26.2.26|xxx|Hubei picorna like virus 71|rv 18 00050058
cls.26.2.26|xxx|Hubei picorna like virus 70|rv 18 00050786
cls.26.2.26|xxx|Beihai picorna like virus 119|rv 18 00050980
cls.26.2.26|xxx|Hubei picorna like virus 75|rv 18 00050240
cls.26.2.26|xxx|Hubei picorna like virus 74|rv 18 00600399
cls.26.2.26|xxx|Hubei picorna like virus 73|rv 18 00050589
cls.26.2.26|xxx|Hubei myriapoda virus 3|rv 18 00051433
cls.26.2.26|xxx|Hubei myriapoda virus 2|rv 18 00051381
cls.26.2.26|xxx|Hubei picorna like virus 76|rv 18 00600564
cls.26.2.26|xxx|Wuhan spider virus 6|rv 18 00049316
cls.26.2.26|Picornavirales|Nephila clavipes virus 2|rv 18 00181596
cls.26.2.26|xxx|Beihai picorna like virus 120|rv 18 00601209
cls.26.2.26|xxx|Beihai sipunculid worm virus 5|rv 18 00050920
cls.26.2.26|xxx|Beihai picorna like virus 121|rv 18 00050825
cls.26.2.26|xxx|Wuhan arthropod virus 3|rv 18 00049505
cls.26.2.26|xxx|Hubei picorna like virus 77|rv 18 00600362
Tree scale: 1
Dataset legend
RdRp
Capsid protein_Jelly-roll
Chymotrypsin-like protease
Capping_methyltransferase_guanylyltransferase
Superfamily 3 helicase
r84.0
Tombusvirus p33_Luteo_P1 domain_auxiliary replication protein
Superfamily 1 helicase
Major capsid protein_Toti-like (T=1)
Putative protease co-factor_calici-como32K-like
Maturation_Levi
Phospholipase A2
VP4_Dicistro
Capsid protein_Levi-like
PrsW-protease
small_ubiquitin-related_modifier_SUMO
Zinc-binding domain
Others

Ov56

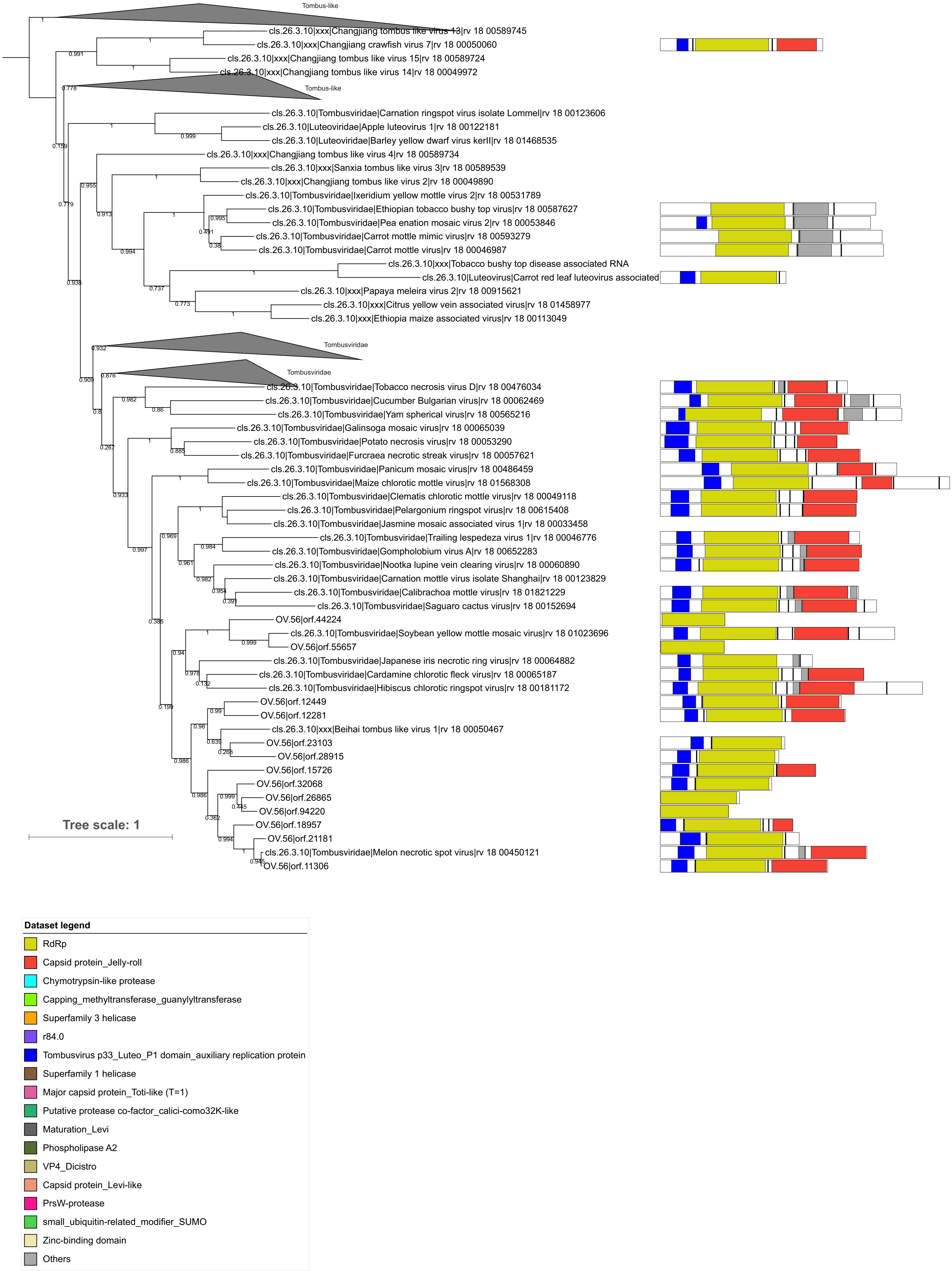

Tombus-like
cls.26.3.10|xxx|Changjiang tombus like virus 13|rv 18 00589745
cls.26.3.10|xxx|Changjiang crawfish virus 7|rv 18 00050060
cls.26.3.10|xxx|Changjiang tombus like virus 15|rv 18 00589724
cls.26.3.10|xxx|Changjiang tombus like virus 14|rv 18 00049972
Tombus-like
cls.26.3.10|Tombusviridae|Carnation ringspot virus isolate Lommel|rv 18 00123606
cls.26.3.10|Luteoviridae|Apple luteovirus 1|rv 18 00122181
cls.26.3.10|Luteoviridae|Barley yellow dwarf virus kerII|rv 18 01468535
cls.26.3.10|xxx|Changjiang tombus like virus 4|rv 18 00589734
cls.26.3.10|xxx|Sanxia tombus like virus 3|rv 18 00589539
cls.26.3.10|xxx|Changjiang tombus like virus 2|rv 18 00049890
cls.26.3.10|Tombusviridae|Ixeridium yellow mottle virus 2|rv 18 00531789
cls.26.3.10|Tombusviridae|Ethiopian tobacco bushy top virus|rv 18 00587627
cls.26.3.10|Tombusviridae|Pea enation mosaic virus 2|rv 18 00053846
cls.26.3.10|Tombusviridae|Carrot mottle mimic virus|rv 18 00593279
cls.26.3.10|Tombusviridae|Carrot mottle virus|rv 18 00046987
cls.26.3.10|xxx|Tobacco bushy top disease associated RNA
cls.26.3.10|Luteovirus|Carrot red leaf luteovirus associated
cls.26.3.10|xxx|Papaya meleira virus 2|rv 18 00915621
cls.26.3.10|xxx|Citrus yellow vein associated virus|rv 18 01458977
cls.26.3.10|xxx|Ethiopia maize associated virus|rv 18 00113049
Tombusviridae
Tombusviridae
cls.26.3.10|Tombusviridae|Tobacco necrosis virus D|rv 18 00476034
cls.26.3.10|Tombusviridae|Cucumber Bulgarian virus|rv 18 00062469
cls.26.3.10|Tombusviridae|Yam spherical virus|rv 18 00565216
cls.26.3.10|Tombusviridae|Galinsoga mosaic virus|rv 18 00065039
cls.26.3.10|Tombusviridae|Potato necrosis virus|rv 18 00053290
cls.26.3.10|Tombusviridae|Furcraea necrotic streak virus|rv 18 00057621
cls.26.3.10|Tombusviridae|Panicum mosaic virus|rv 18 00486459
cls.26.3.10|Tombusviridae|Maize chlorotic mottle virus|rv 18 01568308
cls.26.3.10|Tombusviridae|Clematis chlorotic mottle virus|rv 18 00049118
cls.26.3.10|Tombusviridae|Pelargonium ringspot virus|rv 18 00615408
cls.26.3.10|Tombusviridae|Jasmine mosaic associated virus 1|rv 18 00033458
cls.26.3.10|Tombusviridae|Trailing lespedeza virus 1|rv 18 00046776
cls.26.3.10|Tombusviridae|Gompholobium virus A|rv 18 00652283
cls.26.3.10|Tombusviridae|Nootka lupine vein clearing virus|rv 18 00060890
cls.26.3.10|Tombusviridae|Carnation mottle virus isolate Shanghai|rv 18 00123829
cls.26.3.10|Tombusviridae|Calibrachoa mottle virus|rv 18 01821229
cls.26.3.10|Tombusviridae|Saguaro cactus virus|rv 18 00152694
OV.56|orf.44224
cls.26.3.10|Tombusviridae|Soybean yellow mottle mosaic virus|rv 18 01023696
OV.56|orf.55657
cls.26.3.10|Tombusviridae|Japanese iris necrotic ring virus|rv 18 00064882
cls.26.3.10|Tombusviridae|Cardamine chlorotic fleck virus|rv 18 00065187
cls.26.3.10|Tombusviridae|Hibiscus chlorotic ringspot virus|rv 18 00181172
OV.56|orf.12449
OV.56|orf.12281
cls.26.3.10|xxx|Beihai tombus like virus 1|rv 18 00050467
OV.56|orf.23103
OV.56|orf.28915
OV.56|orf.15726
OV.56|orf.32068
OV.56|orf.26865
OV.56|orf.94220
OV.56|orf.18957
OV.56|orf.21181
cls.26.3.10|Tombusviridae|Melon necrotic spot virus|rv 18 00450121
OV.56|orf.11306
Tree scale: 1
Dataset legend
RdRp
Capsid protein_Jelly-roll
Chymotrypsin-like protease
Capping_methyltransferase_guanylyltransferase
Superfamily 3 helicase
r84.0
Tombusvirus p33_Luteo_P1 domain_auxiliary replication protein
Superfamily 1 helicase
Major capsid protein_Toti-like (T=1)
Putative protease co-factor_calici-como32K-like
Maturation_Levi
Phospholipase A2
VP4_Dicistro
Capsid protein_Levi-like
PrsW-protease
small_ubiquitin-related_modifier_SUMO
Zinc-binding domain
Others

Ov57

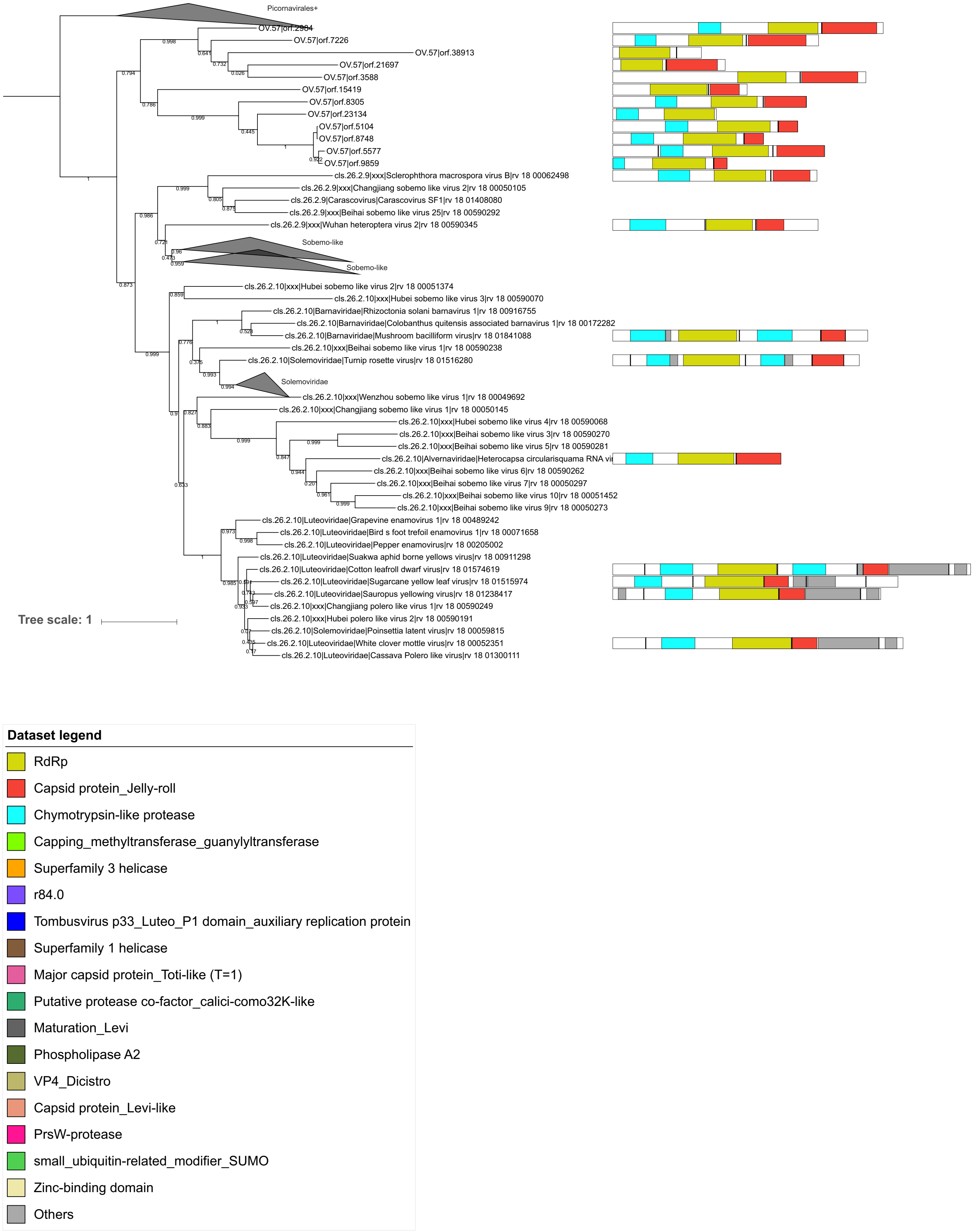
Picornavirales+
OV.57|orf.2984
OV.57|orf.7226
OV.57|orf.38913
OV.57|orf.21697
OV.57|orf.3588
OV.57|orf.15419
OV.57|orf.8305
OV.57|orf.23134
OV.57|orf.5104
OV.57|orf.8748
OV.57|orf.5577
OV.57|orf.9859
cls.26.2.9|xxx|Sclerophthora macrospora virus B|rv 18 00062498
cls.26.2.9|xxx|Changjiang sobemo like virus 2|rv 18 00050105
cls.26.2.9|Carascovirus|Carascovirus SF1|rv 18 01408080
cls.26.2.9|xxx|Beihai sobemo like virus 25|rv 18 00590292
cls.26.2.9|xxx|Wuhan heteroptera virus 2|rv 18 00590345
Sobemo-like
Sobemo-like
cls.26.2.10|xxx|Hubei sobemo like virus 2|rv 18 00051374
cls.26.2.10|xxx|Hubei sobemo like virus 3|rv 18 00590070
cls.26.2.10|Barnaviridae|Rhizoctonia solani barnavirus 1|rv 18 00916755
cls.26.2.10|Barnaviridae|Colobanthus quitensis associated barnavirus 1|rv 18 00172282
cls.26.2.10|Barnaviridae|Mushroom bacilliform virus|rv 18 01841088
cls.26.2.10|xxx|Beihai sobemo like virus 1|rv 18 00590238
cls.26.2.10|Solemoviridae|Turnip rosette virus|rv 18 01516280
Solemoviridae
cls.26.2.10|xxx|Wenzhou sobemo like virus 1|rv 18 00049692
cls.26.2.10|xxx|Changjiang sobemo like virus 1|rv 18 00050145
cls.26.2.10|xxx|Hubei sobemo like virus 4|rv 18 00590068
cls.26.2.10|xxx|Beihai sobemo like virus 3|rv 18 00590270
cls.26.2.10|xxx|Beihai sobemo like virus 5|rv 18 00590281
cls.26.2.10|Alvernaviridae|Heterocapsa circularisquama RNA vi
cls.26.2.10|xxx|Beihai sobemo like virus 6|rv 18 00590262
cls.26.2.10|xxx|Beihai sobemo like virus 7|rv 18 00050297
cls.26.2.10|xxx|Beihai sobemo like virus 10|rv 18 00051452
cls.26.2.10|xxx|Beihai sobemo like virus 9|rv 18 00050273
cls.26.2.10|Luteoviridae|Grapevine enamovirus 1|rv 18 00489242
cls.26.2.10|Luteoviridae|Bird s foot trefoil enamovirus 1|rv 18 00071658
cls.26.2.10|Luteoviridae|Pepper enamovirus|rv 18 00205002
cls.26.2.10|Luteoviridae|Suakwa aphid borne yellows virus|rv 18 00911298
cls.26.2.10|Luteoviridae|Cotton leafroll dwarf virus|rv 18 01574619
cls.26.2.10|Luteoviridae|Sugarcane yellow leaf virus|rv 18 01515974
cls.26.2.10|Luteoviridae|Sauropus yellowing virus|rv 18 01238417
cls.26.2.10|xxx|Changjiang polero like virus 1|rv 18 00590249
cls.26.2.10|xxx|Hubei polero like virus 2|rv 18 00590191
cls.26.2.10|Solemoviridae|Poinsettia latent virus|rv 18 00059815
cls.26.2.10|Luteoviridae|White clover mottle virus|rv 18 00052351
cls.26.2.10|Luteoviridae|Cassava Polero like virus|rv 18 01300111
Tree scale: 1
Dataset legend
RdRp
Capsid protein_Jelly-roll
Chymotrypsin-like protease
Capping_methyltransferase_guanylyltransferase
Superfamily 3 helicase
r84.0
Tombusvirus p33_Luteo_P1 domain_auxiliary replication protein
Superfamily 1 helicase
Major capsid protein_Toti-like (T=1)
Putative protease co-factor_calici-como32K-like
Maturation_Levi
Phospholipase A2
VP4_Dicistro
Capsid protein_Levi-like
PrsW-protease
small_ubiquitin-related_modifier_SUMO
Zinc-binding domain
Others

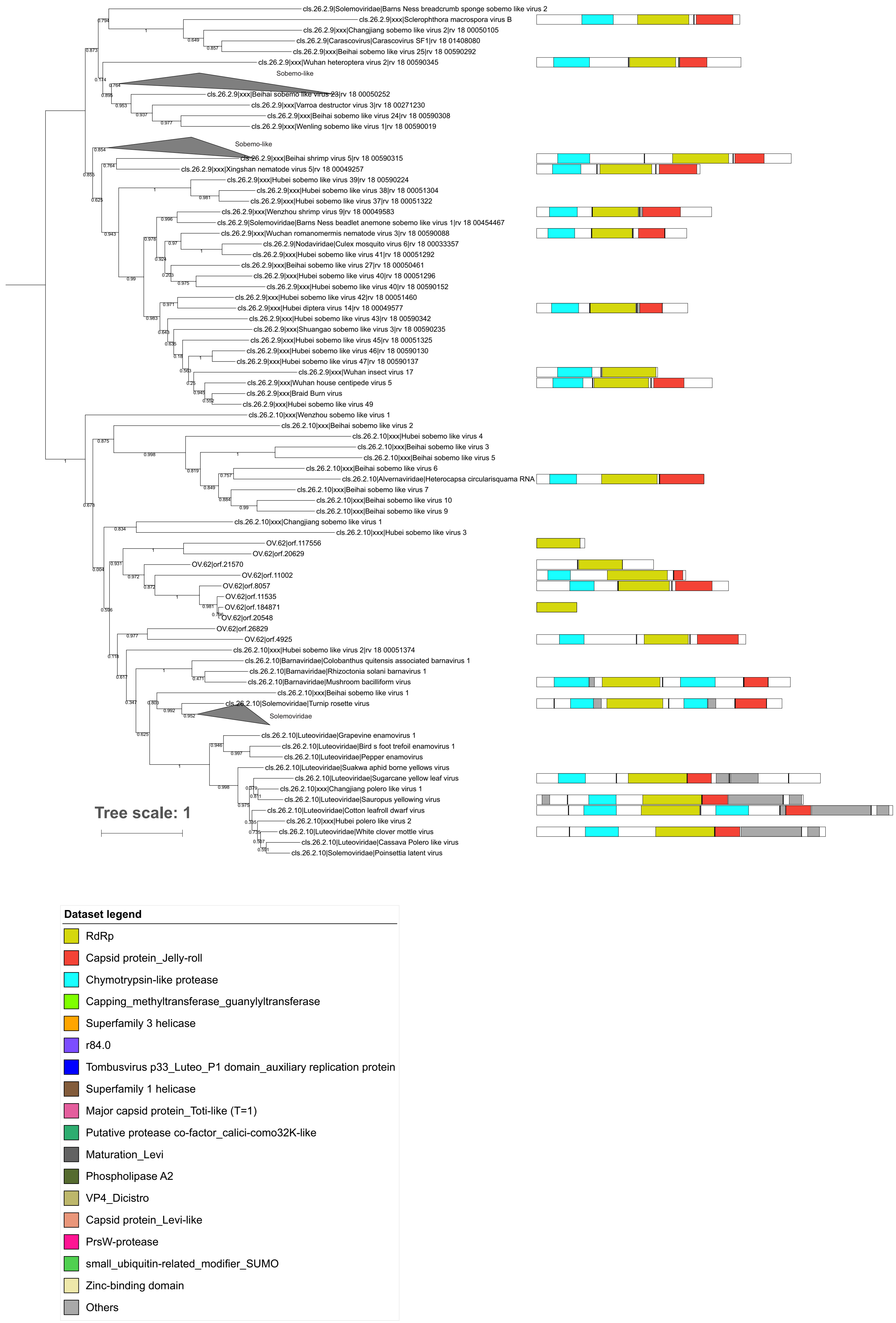

cls.26.2.9|Solemoviridae|Barns Ness breadcrumb sponge sobemo like virus 2
cls.26.2.9|xxx|Sclerophthora macrospora virus B
cls.26.2.9|xxx|Changjiang sobemo like virus 2|rv 18 00050105
cls.26.2.9|Carascovirus|Carascovirus SF1|rv 18 01408080
cls.26.2.9|xxx|Beihai sobemo like virus 25|rv 18 00590292
cls.26.2.9|xxx|Wuhan heteroptera virus 2|rv 18 00590345
Sobemo-like
cls.26.2.9|xxx|Beihai sobemo like virus 23|rv 18 00050252
cls.26.2.9|xxx|Varroa destructor virus 3|rv 18 00271230
cls.26.2.9|xxx|Beihai sobemo like virus 24|rv 18 00590308
cls.26.2.9|xxx|Wenling sobemo like virus 1|rv 18 00590019
Sobemo-like
cls.26.2.9|xxx|Beihai shrimp virus 5|rv 18 00590315
cls.26.2.9|xxx|Xingshan nematode virus 5|rv 18 00049257
cls.26.2.9|xxx|Hubei sobemo like virus 39|rv 18 00590224
cls.26.2.9|xxx|Hubei sobemo like virus 38|rv 18 00051304
cls.26.2.9|xxx|Hubei sobemo like virus 37|rv 18 00051322
cls.26.2.9|xxx|Wenzhou shrimp virus 9|rv 18 00049583
cls.26.2.9|Solemoviridae|Barns Ness beadlet anemone sobemo like virus 1|rv 18 00454467
cls.26.2.9|xxx|Wuchan romanomermis nematode virus 3|rv 18 00590088
cls.26.2.9|Nodaviridae|Culex mosquito virus 6|rv 18 00033357
cls.26.2.9|xxx|Hubei sobemo like virus 41|rv 18 00051292
cls.26.2.9|xxx|Beihai sobemo like virus 27|rv 18 00050461
cls.26.2.9|xxx|Hubei sobemo like virus 40|rv 18 00051296
cls.26.2.9|xxx|Hubei sobemo like virus 40|rv 18 00590152
cls.26.2.9|xxx|Hubei sobemo like virus 42|rv 18 00051460
cls.26.2.9|xxx|Hubei diptera virus 14|rv 18 00049577
cls.26.2.9|xxx|Hubei sobemo like virus 43|rv 18 00590342
cls.26.2.9|xxx|Shuangao sobemo like virus 3|rv 18 00590235
cls.26.2.9|xxx|Hubei sobemo like virus 45|rv 18 00051325
cls.26.2.9|xxx|Hubei sobemo like virus 46|rv 18 00590130
cls.26.2.9|xxx|Hubei sobemo like virus 47|rv 18 00590137
cls.26.2.9|xxx|Wuhan insect virus 17
cls.26.2.9|xxx|Wuhan house centipede virus 5
cls.26.2.9|xxx|Braid Burn virus
cls.26.2.9|xxx|Hubei sobemo like virus 49
cls.26.2.10|xxx|Wenzhou sobemo like virus 1
cls.26.2.10|xxx|Beihai sobemo like virus 2
cls.26.2.10|xxx|Hubei sobemo like virus 4
cls.26.2.10|xxx|Beihai sobemo like virus 3
cls.26.2.10|xxx|Beihai sobemo like virus 5
cls.26.2.10|xxx|Beihai sobemo like virus 6
cls.26.2.10|Alvernaviridae|Heterocapsa circularisquama RNA
cls.26.2.10|xxx|Beihai sobemo like virus 7
cls.26.2.10|xxx|Beihai sobemo like virus 10
cls.26.2.10|xxx|Beihai sobemo like virus 9
cls.26.2.10|xxx|Changjiang sobemo like virus 1
cls.26.2.10|xxx|Hubei sobemo like virus 3
OV.62|orf.117556
OV.62|orf.20629
OV.62|orf.21570
OV.62|orf.11002
OV.62|orf.8057
OV.62|orf.11535
OV.62|orf.184871
OV.62|orf.20548
OV.62|orf.26829
OV.62|orf.4925
cls.26.2.10|xxx|Hubei sobemo like virus 2|rv 18 00051374
cls.26.2.10|Barnaviridae|Colobanthus quitensis associated barnavirus 1
cls.26.2.10|Barnaviridae|Rhizoctonia solani barnavirus 1
cls.26.2.10|Barnaviridae|Mushroom bacilliform virus
cls.26.2.10|xxx|Beihai sobemo like virus 1
cls.26.2.10|Solemoviridae|Turnip rosette virus
Solemoviridae
cls.26.2.10|Luteoviridae|Grapevine enamovirus 1
cls.26.2.10|Luteoviridae|Bird s foot trefoil enamovirus 1
cls.26.2.10|Luteoviridae|Pepper enamovirus
cls.26.2.10|Luteoviridae|Suakwa aphid borne yellows virus
cls.26.2.10|Luteoviridae|Sugarcane yellow leaf virus
cls.26.2.10|xxx|Changjiang polero like virus 1
cls.26.2.10|Luteoviridae|Sauropus yellowing virus
cls.26.2.10|Luteoviridae|Cotton leafroll dwarf virus
cls.26.2.10|xxx|Hubei polero like virus 2
cls.26.2.10|Luteoviridae|White clover mottle virus
cls.26.2.10|Luteoviridae|Cassava Polero like virus
cls.26.2.10|Solemoviridae|Poinsettia latent virus
Tree scale: 1
Dataset legend
RdRp
Capsid protein_Jelly-roll
Chymotrypsin-like protease
Capping_methyltransferase_guanylyltransferase
Superfamily 3 helicase
r84.0
Tombusvirus p33_Luteo_P1 domain_auxiliary replication protein
Superfamily 1 helicase
Major capsid protein_Toti-like (T=1)
Putative protease co-factor_calici-como32K-like
Maturation_Levi
Phospholipase A2
VP4_Dicistro
Capsid protein_Levi-like
PrsW-protease
small_ubiquitin-related_modifier_SUMO
Zinc-binding domain
Others

Ov67

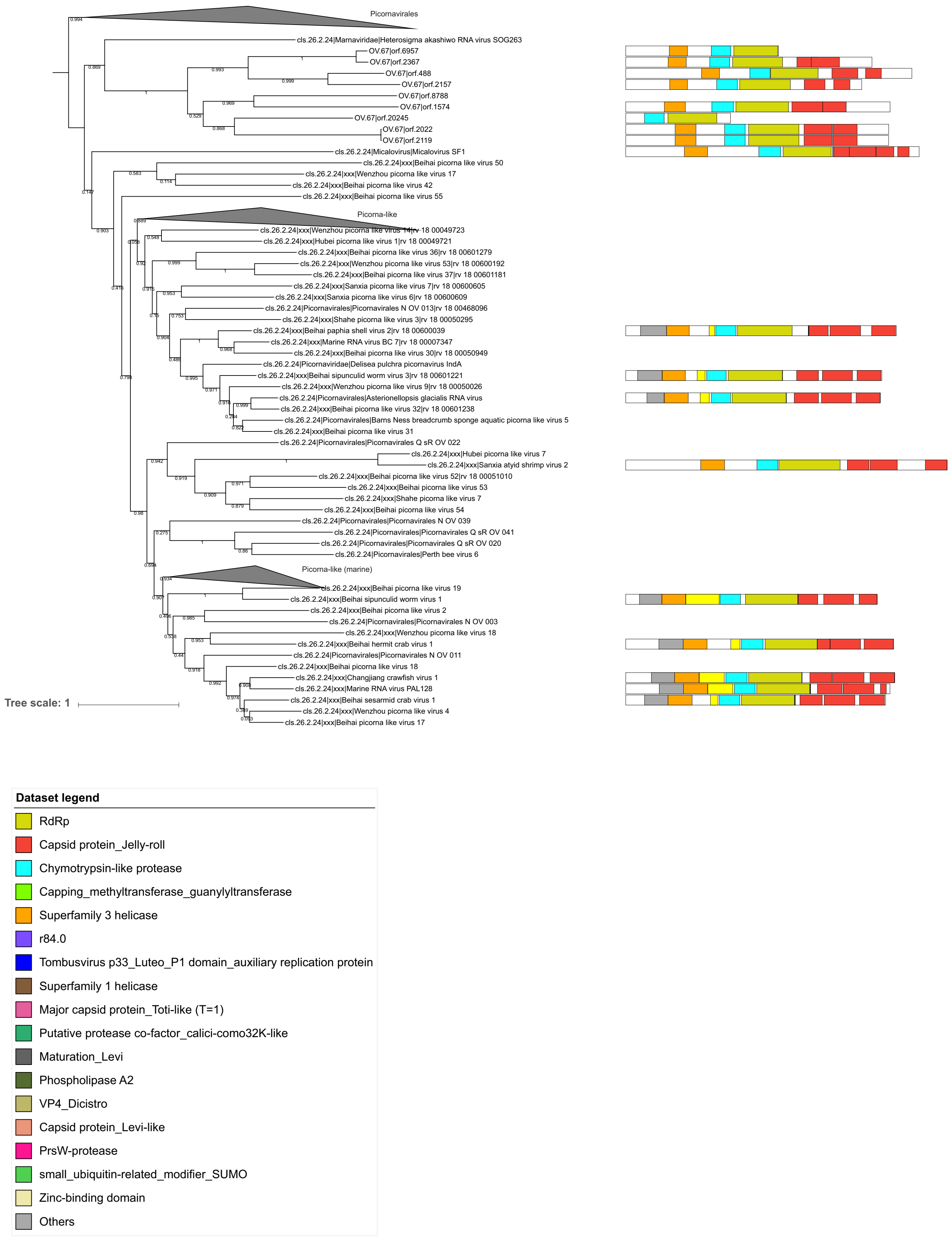

Picornavirales
cls.26.2.24|Marnaviridae|Heterosigma akashiwo RNA virus SOG263
OV.67|orf.6957
OV.67|orf.2367
OV.67|orf.488
OV.67|orf.2157
OV.67|orf.8788
OV.67|orf.1574
OV.67|orf.20245
OV.67|orf.2022
OV.67|orf.2119
cls.26.2.24|Micalovirus|Micalovirus SF1
cls.26.2.24|xxx|Beihai picorna like virus 50
cls.26.2.24|xxx|Wenzhou picorna like virus 17
cls.26.2.24|xxx|Beihai picorna like virus 42
cls.26.2.24|xxx|Beihai picorna like virus 55
Picorna-like
cls.26.2.24|xxx|Wenzhou picorna like virus 14|rv 18 00049723
cls.26.2.24|xxx|Hubei picorna like virus 1|rv 18 00049721
cls.26.2.24|xxx|Beihai picorna like virus 36|rv 18 00601279
cls.26.2.24|xxx|Wenzhou picorna like virus 53|rv 18 00600192
cls.26.2.24|xxx|Beihai picorna like virus 37|rv 18 00601181
cls.26.2.24|xxx|Sanxia picorna like virus 7|rv 18 00600605
cls.26.2.24|xxx|Sanxia picorna like virus 6|rv 18 00600609
cls.26.2.24|Picornavirales|Picornavirales N OV 013|rv 18 00468096
cls.26.2.24|xxx|Shahe picorna like virus 3|rv 18 00050295
cls.26.2.24|xxx|Beihai paphia shell virus 2|rv 18 00600039
cls.26.2.24|xxx|Marine RNA virus BC 7|rv 18 00007347
cls.26.2.24|xxx|Beihai picorna like virus 30|rv 18 00050949
cls.26.2.24|Picornaviridae|Delisea pulchra picornavirus IndA
cls.26.2.24|xxx|Beihai sipunculid worm virus 3|rv 18 00601221
cls.26.2.24|xxx|Wenzhou picorna like virus 9|rv 18 00050026
cls.26.2.24|Picornavirales|Asterionellopsis glacialis RNA virus
cls.26.2.24|xxx|Beihai picorna like virus 32|rv 18 00601238
cls.26.2.24|Picornavirales|Barns Ness breadcrumb sponge aquatic picorna like virus 5
cls.26.2.24|xxx|Beihai picorna like virus 31
cls.26.2.24|Picornavirales|Picornavirales Q sR OV 022
cls.26.2.24|xxx|Hubei picorna like virus 7
cls.26.2.24|xxx|Sanxia atyid shrimp virus 2
cls.26.2.24|xxx|Beihai picorna like virus 52|rv 18 00051010
cls.26.2.24|xxx|Beihai picorna like virus 53
cls.26.2.24|xxx|Shahe picorna like virus 7
cls.26.2.24|xxx|Beihai picorna like virus 54
cls.26.2.24|Picornavirales|Picornavirales N OV 039
cls.26.2.24|Picornavirales|Picornavirales Q sR OV 041
cls.26.2.24|Picornavirales|Picornavirales Q sR OV 020
cls.26.2.24|Picornavirales|Perth bee virus 6
Picorna-like (marine)
cls.26.2.24|xxx|Beihai picorna like virus 19
cls.26.2.24|xxx|Beihai sipunculid worm virus 1
cls.26.2.24|xxx|Beihai picorna like virus 2
cls.26.2.24|Picornavirales|Picornavirales N OV 003
cls.26.2.24|xxx|Wenzhou picorna like virus 18
cls.26.2.24|xxx|Beihai hermit crab virus 1
cls.26.2.24|Picornavirales|Picornavirales N OV 011
cls.26.2.24|xxx|Beihai picorna like virus 18
cls.26.2.24|xxx|Changjiang crawfish virus 1
cls.26.2.24|xxx|Marine RNA virus PAL128
cls.26.2.24|xxx|Beihai sesarmid crab virus 1
cls.26.2.24|xxx|Wenzhou picorna like virus 4
cls.26.2.24|xxx|Beihai picorna like virus 17
Tree scale: 1
Dataset legend
RdRp
Capsid protein_Jelly-roll
Chymotrypsin-like protease
Capping_methyltransferase_guanylyltransferase
Superfamily 3 helicase
r84.0
Tombusvirus p33_Luteo_P1 domain_auxiliary replication protein
Superfamily 1 helicase
Major capsid protein_Toti-like (T=1)
Putative protease co-factor_calici-como32K-like
Maturation_Levi
Phospholipase A2
VP4_Dicistro
Capsid protein_Levi-like
PrsW-protease
small_ubiquitin-related_modifier_SUMO
Zinc-binding domain
Others

Ov73

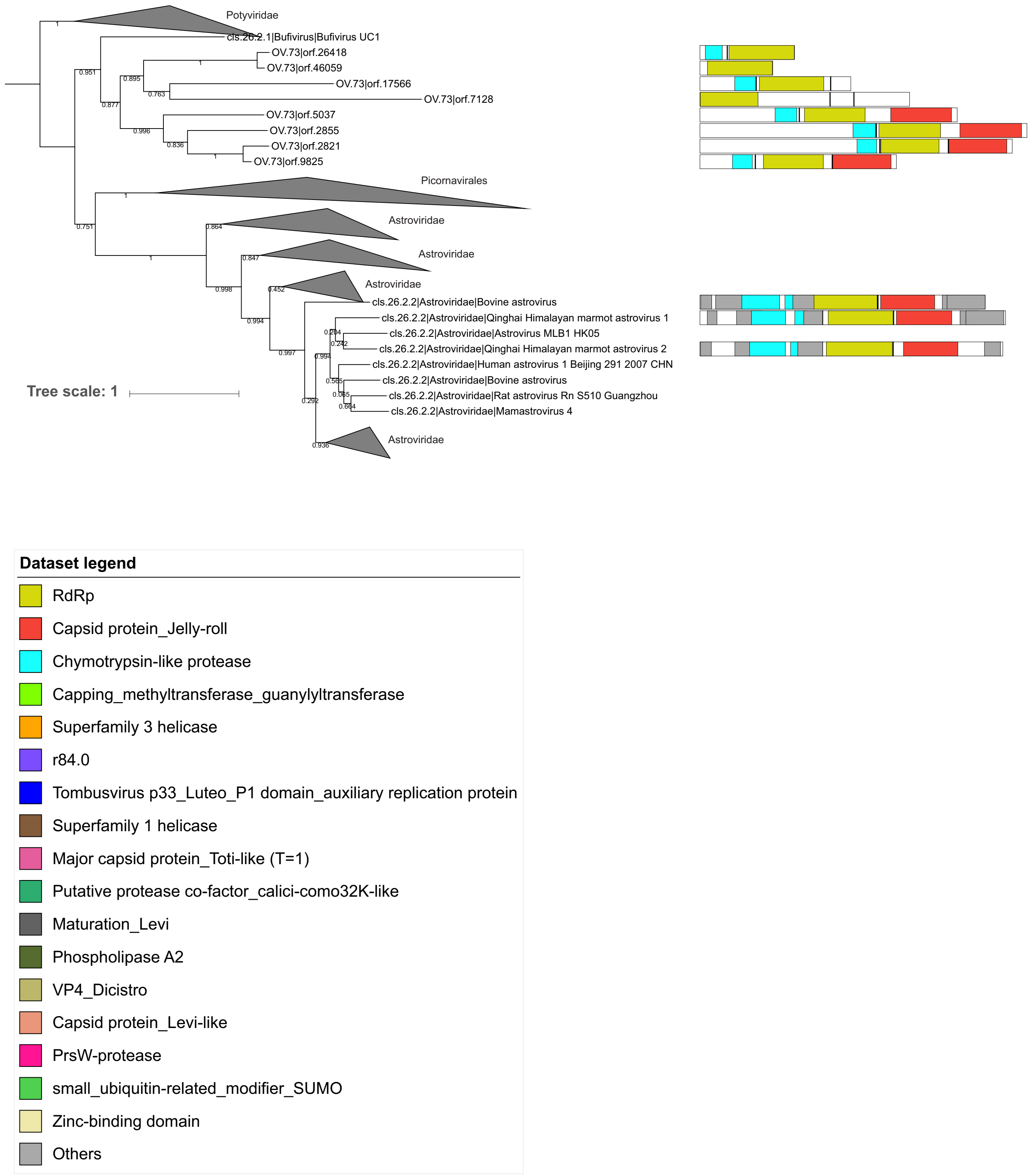
Potyviridae
cls.26.2.1|Bufivirus|Bufivirus UC1
OV.73|orf.26418
OV.73|orf.46059
OV.73|orf.17566
OV.73|orf.7128
OV.73|orf.5037
OV.73|orf.2855
OV.73|orf.2821
OV.73|orf.9825
Picornavirales
Astroviridae
Astroviridae
Astroviridae
cls.26.2.2|Astroviridae|Bovine astrovirus
cls.26.2.2|Astroviridae|Qinghai Himalayan marmot astrovirus 1
cls.26.2.2|Astroviridae|Astrovirus MLB1 HK05
cls.26.2.2|Astroviridae|Qinghai Himalayan marmot astrovirus 2
cls.26.2.2|Astroviridae|Human astrovirus 1 Beijing 291 2007 CHN
cls.26.2.2|Astroviridae|Bovine astrovirus
cls.26.2.2|Astroviridae|Rat astrovirus Rn S510 Guangzhou
cls.26.2.2|Astroviridae|Mamastrovirus 4
Astroviridae
Tree scale: 1
Dataset legend
RdRp
Capsid protein_Jelly-roll
Chymotrypsin-like protease
Capping_methyltransferase_guanylyltransferase
Superfamily 3 helicase
r84.0
Tombusvirus p33_Luteo_P1 domain_auxiliary replication protein
Superfamily 1 helicase
Major capsid protein_Toti-like (T=1)
Putative protease co-factor_calici-como32K-like
Maturation_Levi
Phospholipase A2
VP4_Dicistro
Capsid protein_Levi-like
PrsW-protease
small_ubiquitin-related_modifier_SUMO
Zinc-binding domain
Others

Ov76

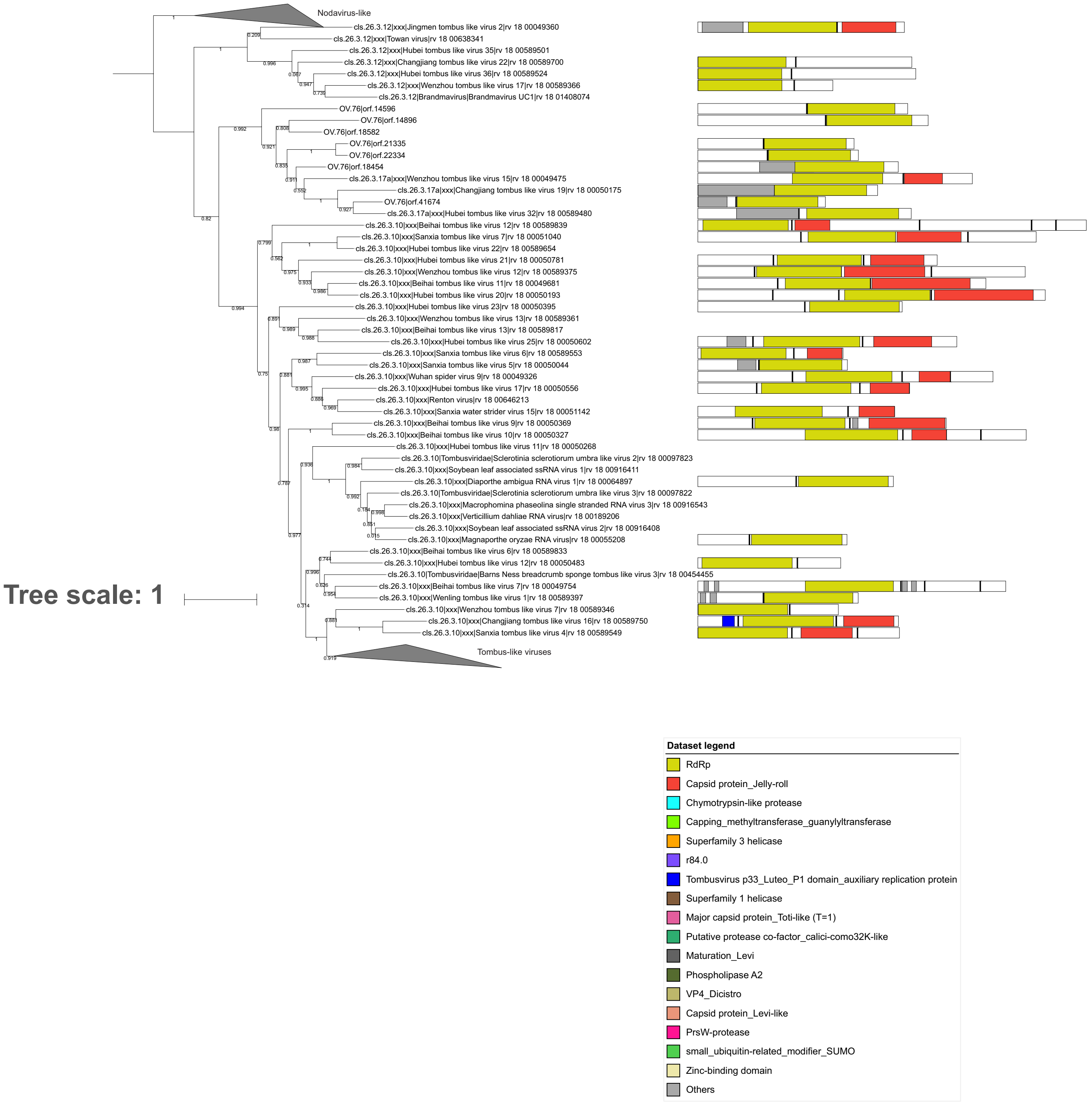
Nodavirus-like
cls.26.3.12|xxx|Jingmen tombus like virus 2|rv 18 00049360
cls.26.3.12|xxx|Towan virus|rv 18 00638341
cls.26.3.12|xxx|Hubei tombus like virus 35|rv 18 00589501
cls.26.3.12|xxx|Changjiang tombus like virus 22|rv 18 00589700
cls.26.3.12|xxx|Hubei tombus like virus 36|rv 18 00589524
cls.26.3.12|xxx|Wenzhou tombus like virus 17|rv 18 00589366
cls.26.3.12|Brandmavirus|Brandmavirus UC1|rv 18 01408074
OV.76|orf.14596
OV.76|orf.14896
OV.76|orf.18582
OV.76|orf.21335
OV.76|orf.22334
OV.76|orf.18454
cls.26.3.17a|xxx|Wenzhou tombus like virus 15|rv 18 00049475
cls.26.3.17a|xxx|Changjiang tombus like virus 19|rv 18 00050175
OV.76|orf.41674
cls.26.3.17a|xxx|Hubei tombus like virus 32|rv 18 00589480
cls.26.3.10|xxx|Beihai tombus like virus 12|rv 18 00589839
cls.26.3.10|xxx|Sanxia tombus like virus 7|rv 18 00051040
cls.26.3.10|xxx|Hubei tombus like virus 22|rv 18 00589654
cls.26.3.10|xxx|Hubei tombus like virus 21|rv 18 00050781
cls.26.3.10|xxx|Wenzhou tombus like virus 12|rv 18 00589375
cls.26.3.10|xxx|Beihai tombus like virus 11|rv 18 00049681
cls.26.3.10|xxx|Hubei tombus like virus 20|rv 18 00050193
cls.26.3.10|xxx|Hubei tombus like virus 23|rv 18 00050395
cls.26.3.10|xxx|Wenzhou tombus like virus 13|rv 18 00589361
cls.26.3.10|xxx|Beihai tombus like virus 13|rv 18 00589817
cls.26.3.10|xxx|Hubei tombus like virus 25|rv 18 00050602
cls.26.3.10|xxx|Sanxia tombus like virus 6|rv 18 00589553
cls.26.3.10|xxx|Sanxia tombus like virus 5|rv 18 00050044
cls.26.3.10|xxx|Wuhan spider virus 9|rv 18 00049326
cls.26.3.10|xxx|Hubei tombus like virus 17|rv 18 00050556
cls.26.3.10|xxx|Renton virus|rv 18 00646213
cls.26.3.10|xxx|Sanxia water strider virus 15|rv 18 00051142
cls.26.3.10|xxx|Beihai tombus like virus 9|rv 18 00050369
cls.26.3.10|xxx|Beihai tombus like virus 10|rv 18 00050327
cls.26.3.10|xxx|Hubei tombus like virus 11|rv 18 00050268
cls.26.3.10|Tombusviridae|Sclerotinia sclerotiorum umbra like virus 2|rv 18 00097823
cls.26.3.10|xxx|Soybean leaf associated ssRNA virus 1|rv 18 00916411
cls.26.3.10|xxx|Diaporthe ambigua RNA virus 1|rv 18 00064897
cls.26.3.10|Tombusviridae|Sclerotinia sclerotiorum umbra like virus 3|rv 18 00097822
cls.26.3.10|xxx|Macrophomina phaseolina single stranded RNA virus 3|rv 18 00916543
cls.26.3.10|xxx|Verticillium dahliae RNA virus|rv 18 00189206
cls.26.3.10|xxx|Soybean leaf associated ssRNA virus 2|rv 18 00916408
cls.26.3.10|xxx|Magnaporthe oryzae RNA virus|rv 18 00055208
cls.26.3.10|xxx|Beihai tombus like virus 6|rv 18 00589833
cls.26.3.10|xxx|Hubei tombus like virus 12|rv 18 00050483
cls.26.3.10|Tombusviridae|Barns Ness breadcrumb sponge tombus like virus 3|rv 18 00454455
cls.26.3.10|xxx|Beihai tombus like virus 7|rv 18 00049754
cls.26.3.10|xxx|Wenling tombus like virus 1|rv 18 00589397
cls.26.3.10|xxx|Wenzhou tombus like virus 7|rv 18 00589346
cls.26.3.10|xxx|Changjiang tombus like virus 16|rv 18 00589750
cls.26.3.10|xxx|Sanxia tombus like virus 4|rv 18 00589549
Tombus-like viruses
Tree scale: 1
Dataset legend
RdRp
Capsid protein_Jelly-roll
Chymotrypsin-like protease
Capping_methyltransferase_guanylyltransferase
Superfamily 3 helicase
r84.0
Tombusvirus p33_Luteo_P1 domain_auxiliary replication protein
Superfamily 1 helicase
Major capsid protein_Toti-like (T=1)
Putative protease co-factor_calici-como32K-like
Maturation_Levi
Phospholipase A2
VP4_Dicistro
Capsid protein_Levi-like
PrsW-protease
small_ubiquitin-related_modifier_SUMO
Zinc-binding domain
Others

Ov81

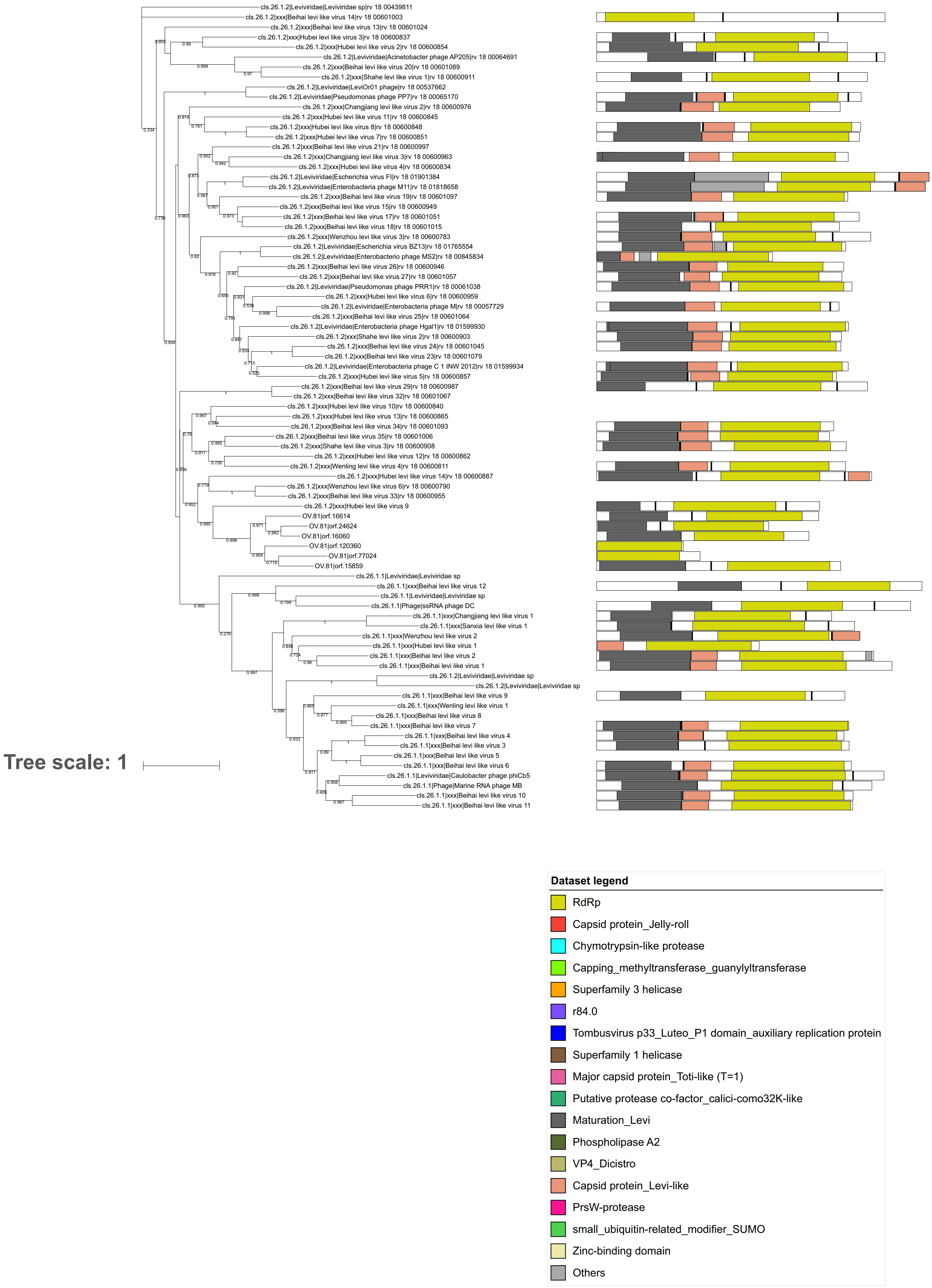
cls.26.1.2|Leviviridae|Leviviridae sp|rv 18 00439811
cls.26.1.2|xxx|Beihai levi like virus 14|rv 18 00601003
cls.26.1.2|xxx|Beihai levi like virus 13|rv 18 00601024
cls.26.1.2|xxx|Hubei levi like virus 3|rv 18 00600837
cls.26.1.2|xxx|Hubei levi like virus 2|rv 18 00600854
cls.26.1.2|Leviviridae|Acinetobacter phage AP205|rv 18 00064691
cls.26.1.2|xxx|Beihai levi like virus 20|rv 18 00601089
cls.26.1.2|xxx|Shahe levi like virus 1|rv 18 00600911
cls.26.1.2|Leviviridae|LeviOr01 phage|rv 18 00537662
cls.26.1.2|Leviviridae|Pseudomonas phage PP7|rv 18 00065170
cls.26.1.2|xxx|Changjiang levi like virus 2|rv 18 00600976
cls.26.1.2|xxx|Hubei levi like virus 11|rv 18 00600845
cls.26.1.2|xxx|Hubei levi like virus 8|rv 18 00600848
cls.26.1.2|xxx|Hubei levi like virus 7|rv 18 00600851
cls.26.1.2|xxx|Beihai levi like virus 21|rv 18 00600997
cls.26.1.2|xxx|Changjiang levi like virus 3|rv 18 00600963
cls.26.1.2|xxx|Hubei levi like virus 4|rv 18 00600834
cls.26.1.2|Leviviridae|Escherichia virus FI|rv 18 01901384
cls.26.1.2|Leviviridae|Enterobacteria phage M11|rv 18 01818658
cls.26.1.2|xxx|Beihai levi like virus 19|rv 18 00601097
cls.26.1.2|xxx|Beihai levi like virus 15|rv 18 00600949
cls.26.1.2|xxx|Beihai levi like virus 17|rv 18 00601051
cls.26.1.2|xxx|Beihai levi like virus 18|rv 18 00601015
cls.26.1.2|xxx|Wenzhou levi like virus 3|rv 18 00600783
cls.26.1.2|Leviviridae|Escherichia virus BZ13|rv 18 01765554
cls.26.1.2|Leviviridae|Enterobacterio phage MS2|rv 18 00845834
cls.26.1.2|xxx|Beihai levi like virus 26|rv 18 00600946
cls.26.1.2|xxx|Beihai levi like virus 27|rv 18 00601057
cls.26.1.2|Leviviridae|Pseudomonas phage PRR1|rv 18 00061038
cls.26.1.2|xxx|Hubei levi like virus 6|rv 18 00600959
cls.26.1.2|Leviviridae|Enterobacteria phage M|rv 18 00057729
cls.26.1.2|xxx|Beihai levi like virus 25|rv 18 00601064
cls.26.1.2|Leviviridae|Enterobacteria phage Hgal1|rv 18 01599930
cls.26.1.2|xxx|Shahe levi like virus 2|rv 18 00600903
cls.26.1.2|xxx|Beihai levi like virus 24|rv 18 00601045
cls.26.1.2|xxx|Beihai levi like virus 23|rv 18 00601079
cls.26.1.2|Leviviridae|Enterobacteria phage C 1 INW 2012|rv 18 01599934
cls.26.1.2|xxx|Hubei levi like virus 5|rv 18 00600857
cls.26.1.2|xxx|Beihai levi like virus 29|rv 18 00600987
cls.26.1.2|xxx|Beihai levi like virus 32|rv 18 00601067
cls.26.1.2|xxx|Hubei levi like virus 10|rv 18 00600840
cls.26.1.2|xxx|Hubei levi like virus 13|rv 18 00600865
cls.26.1.2|xxx|Beihai levi like virus 34|rv 18 00601093
cls.26.1.2|xxx|Beihai levi like virus 35|rv 18 00601006
cls.26.1.2|xxx|Shahe levi like virus 3|rv 18 00600908
cls.26.1.2|xxx|Hubei levi like virus 12|rv 18 00600862
cls.26.1.2|xxx|Wenling levi like virus 4|rv 18 00600811
cls.26.1.2|xxx|Hubei levi like virus 14|rv 18 00600887
cls.26.1.2|xxx|Wenzhou levi like virus 6|rv 18 00600790
cls.26.1.2|xxx|Beihai levi like virus 33|rv 18 00600955
cls.26.1.2|xxx|Hubei levi like virus 9
OV.81|orf.16614
OV.81|orf.24624
OV.81|orf.16060
OV.81|orf.120360
OV.81|orf.77024
OV.81|orf.15859
cls.26.1.1|Leviviridae|Leviviridae sp
cls.26.1.1|xxx|Beihai levi like virus 12
cls.26.1.1|Leviviridae|Leviviridae sp
cls.26.1.1|Phage|ssRNA phage DC
cls.26.1.1|xxx|Changjiang levi like virus 1
cls.26.1.1|xxx|Sanxia levi like virus 1
cls.26.1.1|xxx|Wenzhou levi like virus 2
cls.26.1.1|xxx|Hubei levi like virus 1
cls.26.1.1|xxx|Beihai levi like virus 2
cls.26.1.1|xxx|Beihai levi like virus 1
cls.26.1.2|Leviviridae|Leviviridae sp
cls.26.1.2|Leviviridae|Leviviridae sp
cls.26.1.1|xxx|Beihai levi like virus 9
cls.26.1.1|xxx|Wenling levi like virus 1
cls.26.1.1|xxx|Beihai levi like virus 8
cls.26.1.1|xxx|Beihai levi like virus 7
cls.26.1.1|xxx|Beihai levi like virus 4
cls.26.1.1|xxx|Beihai levi like virus 3
cls.26.1.1|xxx|Beihai levi like virus 5
cls.26.1.1|xxx|Beihai levi like virus 6
cls.26.1.1|Leviviridae|Caulobacter phage phiCb5
cls.26.1.1|Phage|Marine RNA phage MB
cls.26.1.1|xxx|Beihai levi like virus 10
cls.26.1.1|xxx|Beihai levi like virus 11
Tree scale: 1
Dataset legend
RdRp
Capsid protein_Jelly-roll
Chymotrypsin-like protease
Capping_methyltransferase_guanylyltransferase
Superfamily 3 helicase
r84.0
Tombusvirus p33_Luteo_P1 domain_auxiliary replication protein
Superfamily 1 helicase
Major capsid protein_Toti-like (T=1)
Putative protease co-factor_calici-como32K-like
Maturation_Levi
Phospholipase A2
VP4_Dicistro
Capsid protein_Levi-like
PrsW-protease
small_ubiquitin-related_modifier_SUMO
Zinc-binding domain
Others

Ov85

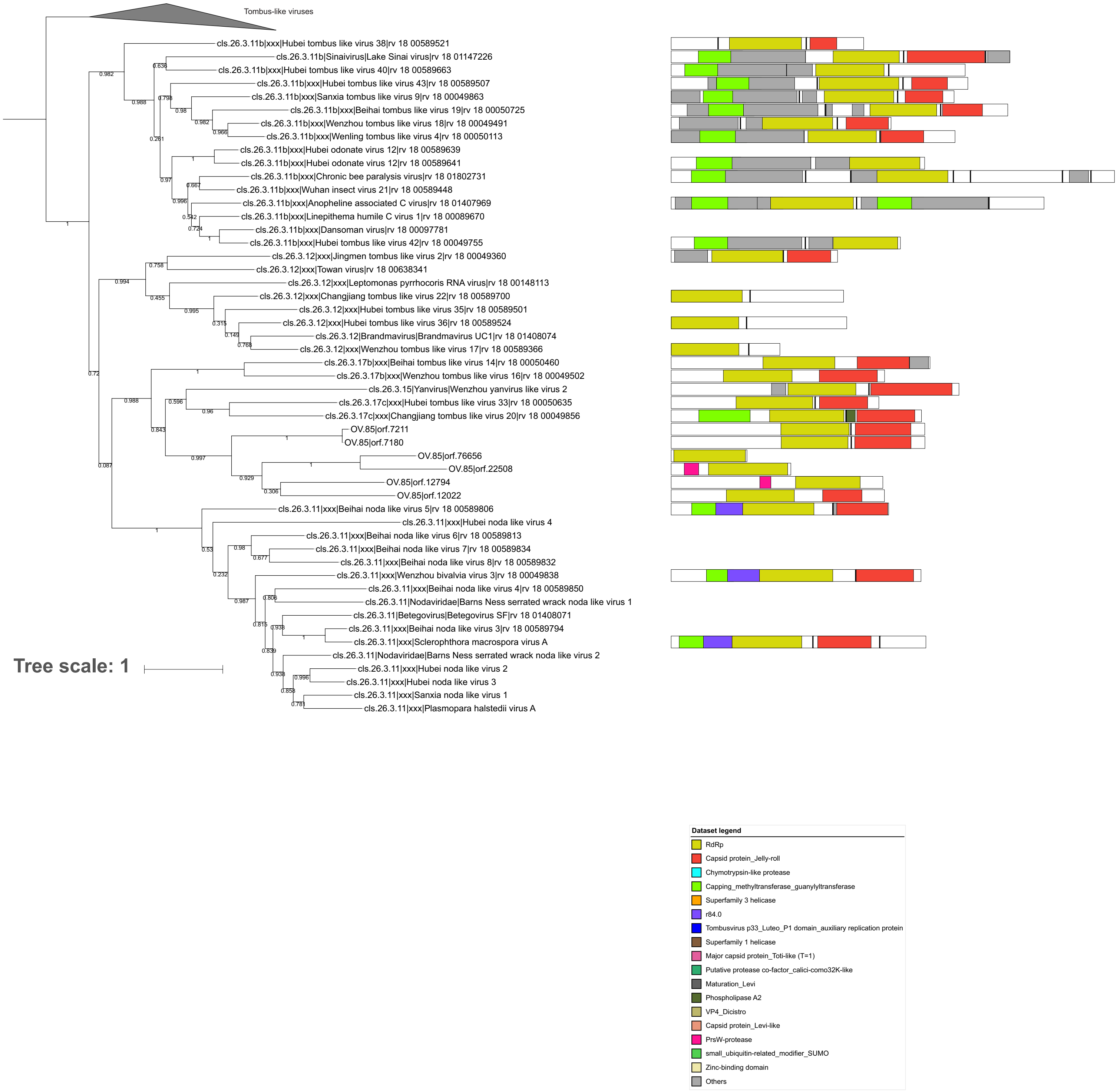

Tombus-like viruses
cls.26.3.11b|xxx|Hubei tombus like virus 38|rv 18 00589521
cls.26.3.11b|Sinaivirus|Lake Sinai virus|rv 18 01147226
cls.26.3.11b|xxx|Hubei tombus like virus 40|rv 18 00589663
cls.26.3.11b|xxx|Hubei tombus like virus 43|rv 18 00589507
cls.26.3.11b|xxx|Sanxia tombus like virus 9|rv 18 00049863
cls.26.3.11b|xxx|Beihai tombus like virus 19|rv 18 00050725
cls.26.3.11b|xxx|Wenzhou tombus like virus 18|rv 18 00049491
cls.26.3.11b|xxx|Wenling tombus like virus 4|rv 18 00050113
cls.26.3.11b|xxx|Hubei odonate virus 12|rv 18 00589639
cls.26.3.11b|xxx|Hubei odonate virus 12|rv 18 00589641
cls.26.3.11b|xxx|Chronic bee paralysis virus|rv 18 01802731
cls.26.3.11b|xxx|Wuhan insect virus 21|rv 18 00589448
cls.26.3.11b|xxx|Anopheline associated C virus|rv 18 01407969
cls.26.3.11b|xxx|Linepithema humile C virus 1|rv 18 00089670
cls.26.3.11b|xxx|Dansoman virus|rv 18 00097781
cls.26.3.11b|xxx|Hubei tombus like virus 42|rv 18 00049755
cls.26.3.12|xxx|Jingmen tombus like virus 2|rv 18 00049360
cls.26.3.12|xxx|Towan virus|rv 18 00638341
cls.26.3.12|xxx|Leptomonas pyrrhocoris RNA virus|rv 18 00148113
cls.26.3.12|xxx|Changjiang tombus like virus 22|rv 18 00589700
cls.26.3.12|xxx|Hubei tombus like virus 35|rv 18 00589501
cls.26.3.12|xxx|Hubei tombus like virus 36|rv 18 00589524
cls.26.3.12|Brandmavirus|Brandmavirus UC1|rv 18 01408074
cls.26.3.12|xxx|Wenzhou tombus like virus 17|rv 18 00589366
cls.26.3.17b|xxx|Beihai tombus like virus 14|rv 18 00050460
cls.26.3.17b|xxx|Wenzhou tombus like virus 16|rv 18 00049502
cls.26.3.15|Yanvirus|Wenzhou yanvirus like virus 2
cls.26.3.17c|xxx|Hubei tombus like virus 33|rv 18 00050635
cls.26.3.17c|xxx|Changjiang tombus like virus 20|rv 18 00049856
OV.85|orf.7211
OV.85|orf.7180
OV.85|orf.76656
OV.85|orf.22508
OV.85|orf.12794
OV.85|orf.12022
cls.26.3.11|xxx|Beihai noda like virus 5|rv 18 00589806
cls.26.3.11|xxx|Hubei noda like virus 4
cls.26.3.11|xxx|Beihai noda like virus 6|rv 18 00589813
cls.26.3.11|xxx|Beihai noda like virus 7|rv 18 00589834
cls.26.3.11|xxx|Beihai noda like virus 8|rv 18 00589832
cls.26.3.11|xxx|Wenzhou bivalvia virus 3|rv 18 00049838
cls.26.3.11|xxx|Beihai noda like virus 4|rv 18 00589850
cls.26.3.11|Nodaviridae|Barns Ness serrated wrack noda like virus 1
cls.26.3.11|Betegovirus|Betegovirus SF|rv 18 01408071
cls.26.3.11|xxx|Beihai noda like virus 3|rv 18 00589794
cls.26.3.11|xxx|Sclerophthora macrospora virus A
cls.26.3.11|Nodaviridae|Barns Ness serrated wrack noda like virus 2
cls.26.3.11|xxx|Hubei noda like virus 2
cls.26.3.11|xxx|Hubei noda like virus 3
cls.26.3.11|xxx|Sanxia noda like virus 1
cls.26.3.11|xxx|Plasmopara halstedii virus A
Tree scale: 1
Dataset legend
RdRp
Capsid protein_Jelly-roll
Chymotrypsin-like protease
Capping_methyltransferase_guanylyltransferase
Superfamily 3 helicase
r84.0
Tombusvirus p33_Luteo_P1 domain_auxiliary replication protein
Superfamily 1 helicase
Major capsid protein_Toti-like (T=1)
Putative protease co-factor_calici-como32K-like
Maturation_Levi
Phospholipase A2
VP4_Dicistro
Capsid protein_Levi-like
PrsW-protease
small_ubiquitin-related_modifier_SUMO
Zinc-binding domain
Others

Ov89

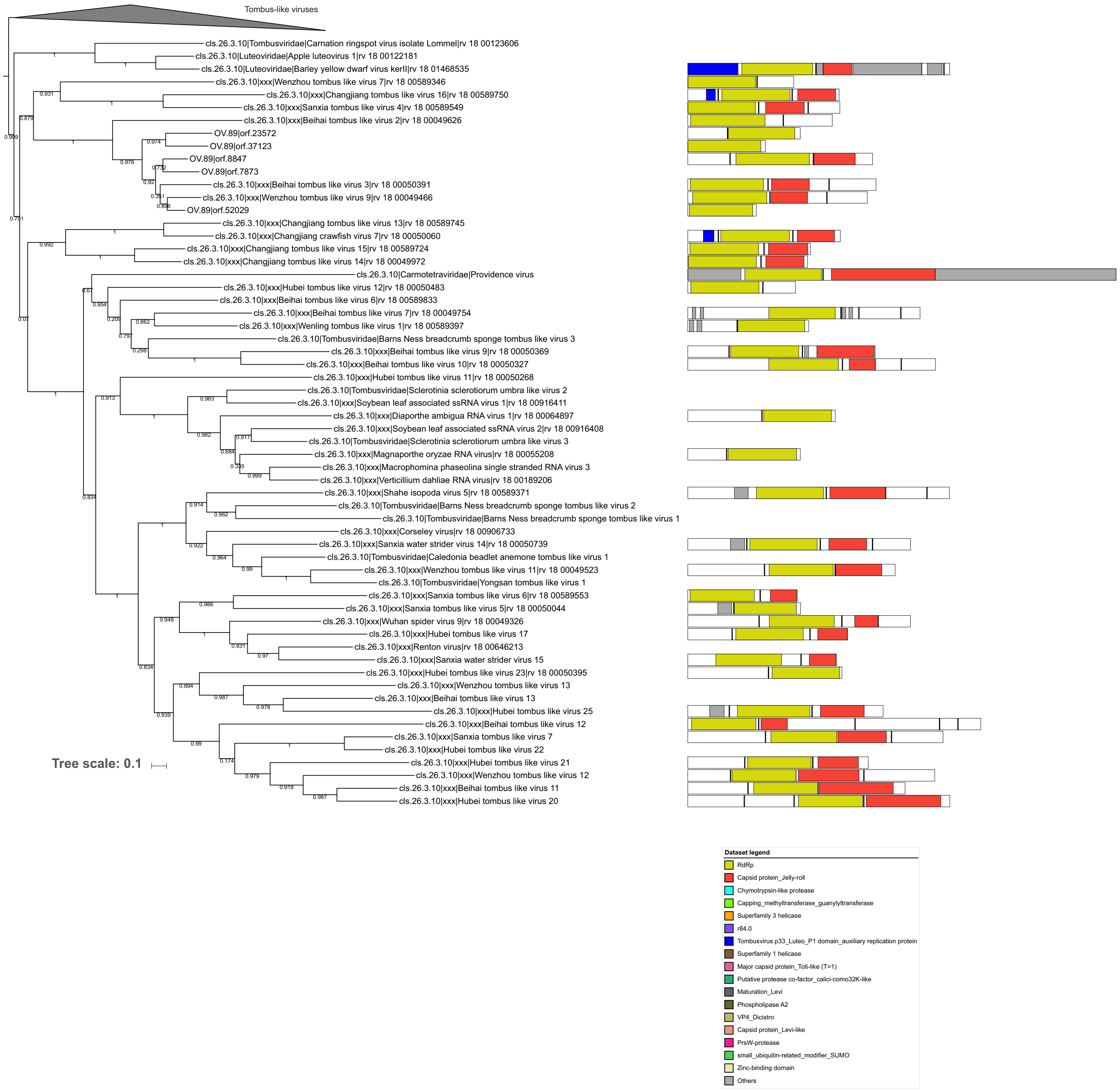

Tombus-like viruses
cls.26.3.10|Tombusviridae|Carnation ringspot virus isolate Lommel|rv 18 00123606
cls.26.3.10|Luteoviridae|Apple luteovirus 1|rv 18 00122181
cls.26.3.10|Luteoviridae|Barley yellow dwarf virus kerII|rv 18 01468535
cls.26.3.10|xxx|Wenzhou tombus like virus 7|rv 18 00589346
cls.26.3.10|xxx|Changjiang tombus like virus 16|rv 18 00589750
cls.26.3.10|xxx|Sanxia tombus like virus 4|rv 18 00589549
cls.26.3.10|xxx|Beihai tombus like virus 2|rv 18 00049626
OV.89|orf.23572
OV.89|orf.37123
OV.89|orf.8847
OV.89|orf.7873
cls.26.3.10|xxx|Beihai tombus like virus 3|rv 18 00050391
cls.26.3.10|xxx|Wenzhou tombus like virus 9|rv 18 00049466
OV.89|orf.52029
cls.26.3.10|xxx|Changjiang tombus like virus 13|rv 18 00589745
cls.26.3.10|xxx|Changjiang crawfish virus 7|rv 18 00050060
cls.26.3.10|xxx|Changjiang tombus like virus 15|rv 18 00589724
cls.26.3.10|xxx|Changjiang tombus like virus 14|rv 18 00049972
cls.26.3.10|Carmotetraviridae|Providence virus
cls.26.3.10|xxx|Hubei tombus like virus 12|rv 18 00050483
cls.26.3.10|xxx|Beihai tombus like virus 6|rv 18 00589833
cls.26.3.10|xxx|Beihai tombus like virus 7|rv 18 00049754
cls.26.3.10|xxx|Wenling tombus like virus 1|rv 18 00589397
cls.26.3.10|Tombusviridae|Barns Ness breadcrumb sponge tombus like virus 3
cls.26.3.10|xxx|Beihai tombus like virus 9|rv 18 00050369
cls.26.3.10|xxx|Beihai tombus like virus 10|rv 18 00050327
cls.26.3.10|xxx|Hubei tombus like virus 11|rv 18 00050268
cls.26.3.10|Tombusviridae|Sclerotinia sclerotiorum umbra like virus 2
cls.26.3.10|xxx|Soybean leaf associated ssRNA virus 1|rv 18 00916411
cls.26.3.10|xxx|Diaporthe ambigua RNA virus 1|rv 18 00064897
cls.26.3.10|xxx|Soybean leaf associated ssRNA virus 2|rv 18 00916408
cls.26.3.10|Tombusviridae|Sclerotinia sclerotiorum umbra like virus 3
cls.26.3.10|xxx|Magnaporthe oryzae RNA virus|rv 18 00055208
cls.26.3.10|xxx|Macrophomina phaseolina single stranded RNA virus 3
cls.26.3.10|xxx|Verticillium dahliae RNA virus|rv 18 00189206
cls.26.3.10|xxx|Shahe isopoda virus 5|rv 18 00589371
cls.26.3.10|Tombusviridae|Barns Ness breadcrumb sponge tombus like virus 2
cls.26.3.10|Tombusviridae|Barns Ness breadcrumb sponge tombus like virus 1
cls.26.3.10|xxx|Corseley virus|rv 18 00906733
cls.26.3.10|xxx|Sanxia water strider virus 14|rv 18 00050739
cls.26.3.10|Tombusviridae|Caledonia beadlet anemone tombus like virus 1
cls.26.3.10|xxx|Wenzhou tombus like virus 11|rv 18 00049523
cls.26.3.10|Tombusviridae|Yongsan tombus like virus 1
cls.26.3.10|xxx|Sanxia tombus like virus 6|rv 18 00589553
cls.26.3.10|xxx|Sanxia tombus like virus 5|rv 18 00050044
cls.26.3.10|xxx|Wuhan spider virus 9|rv 18 00049326
cls.26.3.10|xxx|Hubei tombus like virus 17
cls.26.3.10|xxx|Renton virus|rv 18 00646213
cls.26.3.10|xxx|Sanxia water strider virus 15
cls.26.3.10|xxx|Hubei tombus like virus 23|rv 18 00050395
cls.26.3.10|xxx|Wenzhou tombus like virus 13
cls.26.3.10|xxx|Beihai tombus like virus 13
cls.26.3.10|xxx|Hubei tombus like virus 25
cls.26.3.10|xxx|Beihai tombus like virus 12
cls.26.3.10|xxx|Sanxia tombus like virus 7
cls.26.3.10|xxx|Hubei tombus like virus 22
cls.26.3.10|xxx|Hubei tombus like virus 21
cls.26.3.10|xxx|Wenzhou tombus like virus 12
cls.26.3.10|xxx|Beihai tombus like virus 11
cls.26.3.10|xxx|Hubei tombus like virus 20
Tree scale: 0.1
Dataset legend
RdRp
Capsid protein_Jelly-roll
Chymotrypsin-like protease
Capping_methyltransferase_guanylyltransferase
Superfamily 3 helicase
r84.0
Tombusvirus p33_Luteo_P1 domain_auxiliary replication protein
Superfamily 1 helicase
Major capsid protein_Toti-like (T=1)
Putative protease co-factor_calici-como32K-like
Maturation_Levi
Phospholipase A2
VP4_Dicistro
Capsid protein_Levi-like
PrsW-protease
small_ubiquitin-related_modifier_SUMO
Zinc-binding domain
Others

Ov123

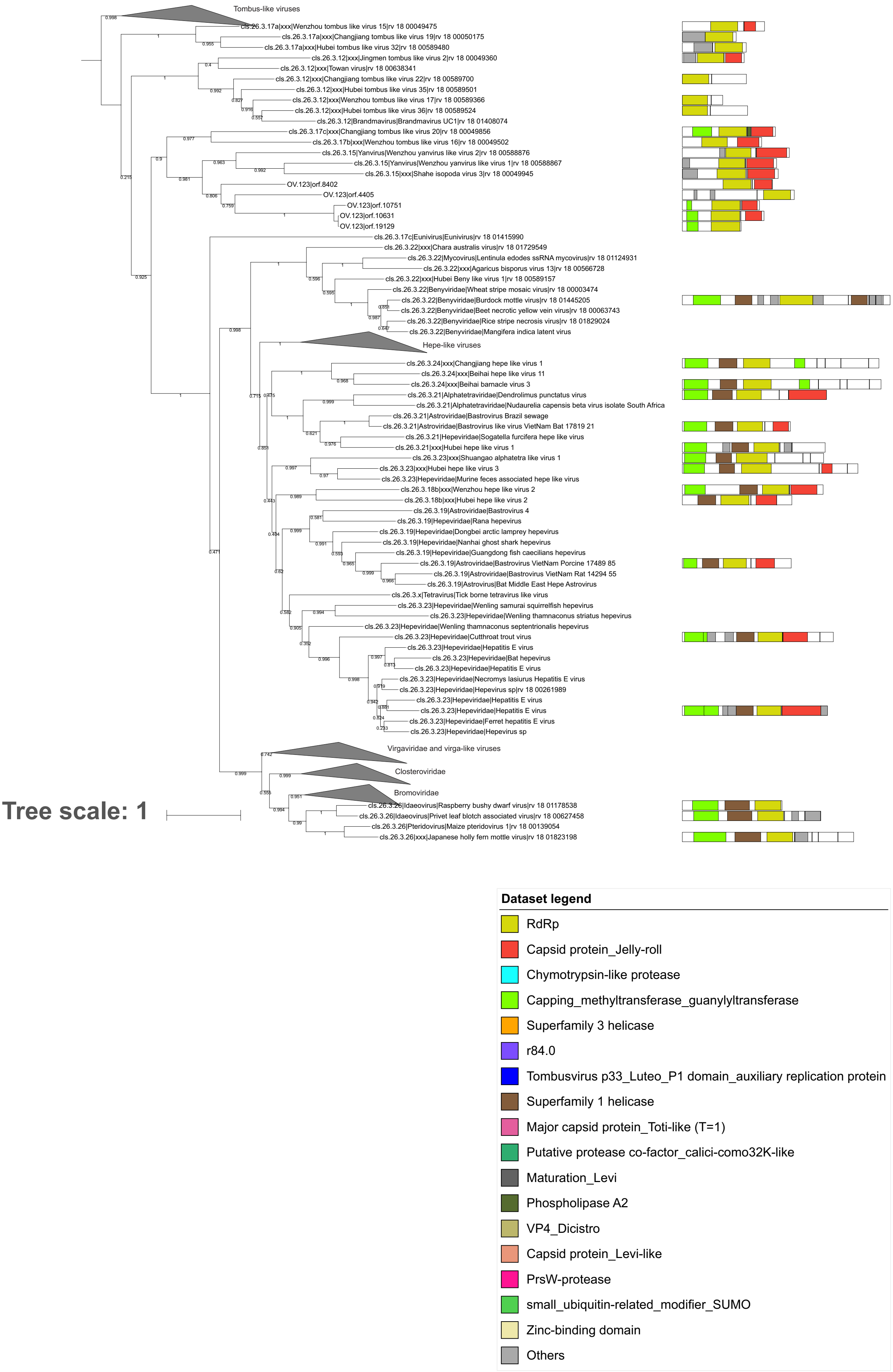

Tombus-like viruses
cls.26.3.17a|xxx|Wenzhou tombus like virus 15|rv 18 00049475
cls.26.3.17a|xxx|Changjiang tombus like virus 19|rv 18 00050175
cls.26.3.17a|xxx|Hubei tombus like virus 32|rv 18 00589480
cls.26.3.12|xxx|Jingmen tombus like virus 2|rv 18 00049360
cls.26.3.12|xxx|Towan virus|rv 18 00638341
cls.26.3.12|xxx|Changjiang tombus like virus 22|rv 18 00589700
cls.26.3.12|xxx|Hubei tombus like virus 35|rv 18 00589501
cls.26.3.12|xxx|Wenzhou tombus like virus 17|rv 18 00589366
cls.26.3.12|xxx|Hubei tombus like virus 36|rv 18 00589524
cls.26.3.12|Brandmavirus|Brandmavirus UC1|rv 18 01408074
cls.26.3.17c|xxx|Changjiang tombus like virus 20|rv 18 00049856
cls.26.3.17b|xxx|Wenzhou tombus like virus 16|rv 18 00049502
cls.26.3.15|Yanvirus|Wenzhou yanvirus like virus 2|rv 18 00588876
cls.26.3.15|Yanvirus|Wenzhou yanvirus like virus 1|rv 18 00588867
cls.26.3.15|xxx|Shahe isopoda virus 3|rv 18 00049945
OV.123|orf.8402
OV.123|orf.4405
OV.123|orf.10751
OV.123|orf.10631
OV.123|orf.19129
cls.26.3.17c|Eunivirus|Eunivirus|rv 18 01415990
cls.26.3.22|xxx|Chara australis virus|rv 18 01729549
cls.26.3.22|Mycovirus|Lentinula edodes ssRNA mycovirus|rv 18 01124931
cls.26.3.22|xxx|Agaricus bisporus virus 13|rv 18 00566728
cls.26.3.22|xxx|Hubei Beny like virus 1|rv 18 00589157
cls.26.3.22|Benyviridae|Wheat stripe mosaic virus|rv 18 00003474
cls.26.3.22|Benyviridae|Burdock mottle virus|rv 18 01445205
cls.26.3.22|Benyviridae|Beet necrotic yellow vein virus|rv 18 00063743
cls.26.3.22|Benyviridae|Rice stripe necrosis virus|rv 18 01829024
cls.26.3.22|Benyviridae|Mangifera indica latent virus
Hepe-like viruses
cls.26.3.24|xxx|Changjiang hepe like virus 1
cls.26.3.24|xxx|Beihai hepe like virus 11
cls.26.3.24|xxx|Beihai barnacle virus 3
cls.26.3.21|Alphatetraviridae|Dendrolimus punctatus virus
cls.26.3.21|Alphatetraviridae|Nudaurelia capensis beta virus isolate South Africa
cls.26.3.21|Astroviridae|Bastrovirus Brazil sewage
cls.26.3.21|Astroviridae|Bastrovirus like virus VietNam Bat 17819 21
cls.26.3.21|Hepeviridae|Sogatella furcifera hepe like virus
cls.26.3.21|xxx|Hubei hepe like virus 1
cls.26.3.23|xxx|Shuangao alphatetra like virus 1
cls.26.3.23|xxx|Hubei hepe like virus 3
cls.26.3.23|Hepeviridae|Murine feces associated hepe like virus
cls.26.3.18b|xxx|Wenzhou hepe like virus 2
cls.26.3.18b|xxx|Hubei hepe like virus 2
cls.26.3.19|Astroviridae|Bastrovirus 4
cls.26.3.19|Hepeviridae|Rana hepevirus
cls.26.3.19|Hepeviridae|Dongbei arctic lamprey hepevirus
cls.26.3.19|Hepeviridae|Nanhai ghost shark hepevirus
cls.26.3.19|Hepeviridae|Guangdong fish caecilians hepevirus
cls.26.3.19|Astroviridae|Bastrovirus VietNam Porcine 17489 85
cls.26.3.19|Astroviridae|Bastrovirus VietNam Rat 14294 55
cls.26.3.19|Astroviridae|Bat Middle East Hepe Astrovirus
cls.26.3.x|Tetravirus|Tick borne tetravirus like virus
cls.26.3.23|Hepeviridae|Wenling samurai squirrelfish hepevirus
cls.26.3.23|Hepeviridae|Wenling thamnaconus striatus hepevirus
cls.26.3.23|Hepeviridae|Wenling thamnaconus septentrionalis hepevirus
cls.26.3.23|Hepeviridae|Cutthroat trout virus
cls.26.3.23|Hepeviridae|Hepatitis E virus
cls.26.3.23|Hepeviridae|Bat hepevirus
cls.26.3.23|Hepeviridae|Hepatitis E virus
cls.26.3.23|Hepeviridae|Necromys lasiurus Hepatitis E virus
cls.26.3.23|Hepeviridae|Hepevirus sp|rv 18 00261989
cls.26.3.23|Hepeviridae|Hepatitis E virus
cls.26.3.23|Hepeviridae|Hepatitis E virus
cls.26.3.23|Hepeviridae|Ferret hepatitis E virus
cls.26.3.23|Hepeviridae|Hepevirus sp
Virgaviridae and virga-like viruses
Closteroviridae
Bromoviridae
cls.26.3.26|Idaeovirus|Raspberry bushy dwarf virus|rv 18 01178538
cls.26.3.26|Idaeovirus|Privet leaf blotch associated virus|rv 18 00627458
cls.26.3.26|Pteridovirus|Maize pteridovirus 1|rv 18 00139054
cls.26.3.26|xxx|Japanese holly fern mottle virus|rv 18 01823198
Tree scale: 1
Dataset legend
RdRp
Capsid protein_Jelly-roll
Chymotrypsin-like protease
Capping_methyltransferase_guanylyltransferase
Superfamily 3 helicase
r84.0
Tombusvirus p33_Luteo_P1 domain_auxiliary replication protein
Superfamily 1 helicase
Major capsid protein_Toti-like (T=1)
Putative protease co-factor_calici-como32K-like
Maturation_Levi
Phospholipase A2
VP4_Dicistro
Capsid protein_Levi-like
PrsW-protease
small_ubiquitin-related_modifier_SUMO
Zinc-binding domain
Others

Ov161

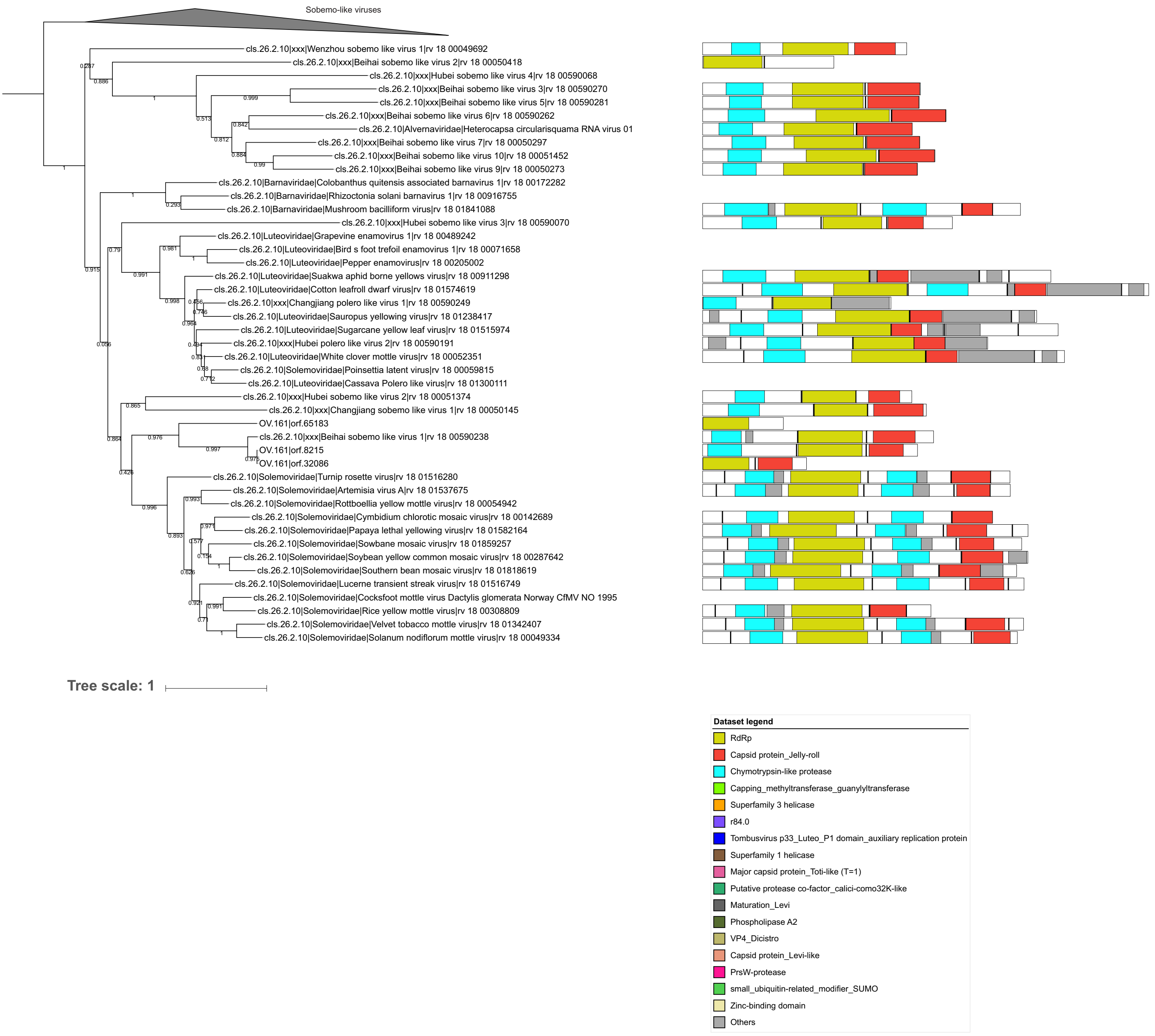
Sobemo-like viruses
cls.26.2.10|xxx|Wenzhou sobemo like virus 1|rv 18 00049692
cls.26.2.10|xxx|Beihai sobemo like virus 2|rv 18 00050418
cls.26.2.10|xxx|Hubei sobemo like virus 4|rv 18 00590068
cls.26.2.10|xxx|Beihai sobemo like virus 3|rv 18 00590270
cls.26.2.10|xxx|Beihai sobemo like virus 5|rv 18 00590281
cls.26.2.10|xxx|Beihai sobemo like virus 6|rv 18 00590262
cls.26.2.10|Alvernaviridae|Heterocapsa circularisquama RNA virus 01
cls.26.2.10|xxx|Beihai sobemo like virus 7|rv 18 00050297
cls.26.2.10|xxx|Beihai sobemo like virus 10|rv 18 00051452
cls.26.2.10|xxx|Beihai sobemo like virus 9|rv 18 00050273
cls.26.2.10|Barnaviridae|Colobanthus quitensis associated barnavirus 1|rv 18 00172282
cls.26.2.10|Barnaviridae|Rhizoctonia solani barnavirus 1|rv 18 00916755
cls.26.2.10|Barnaviridae|Mushroom bacilliform virus|rv 18 01841088
cls.26.2.10|xxx|Hubei sobemo like virus 3|rv 18 00590070
cls.26.2.10|Luteoviridae|Grapevine enamovirus 1|rv 18 00489242
cls.26.2.10|Luteoviridae|Bird s foot trefoil enamovirus 1|rv 18 00071658
cls.26.2.10|Luteoviridae|Pepper enamovirus|rv 18 00205002
cls.26.2.10|Luteoviridae|Suakwa aphid borne yellows virus|rv 18 00911298
cls.26.2.10|Luteoviridae|Cotton leafroll dwarf virus|rv 18 01574619
cls.26.2.10|xxx|Changjiang polero like virus 1|rv 18 00590249
cls.26.2.10|Luteoviridae|Sauropus yellowing virus|rv 18 01238417
cls.26.2.10|Luteoviridae|Sugarcane yellow leaf virus|rv 18 01515974
cls.26.2.10|xxx|Hubei polero like virus 2|rv 18 00590191
cls.26.2.10|Luteoviridae|White clover mottle virus|rv 18 00052351
cls.26.2.10|Solemoviridae|Poinsettia latent virus|rv 18 00059815
cls.26.2.10|Luteoviridae|Cassava Polero like virus|rv 18 01300111
cls.26.2.10|xxx|Hubei sobemo like virus 2|rv 18 00051374
cls.26.2.10|xxx|Changjiang sobemo like virus 1|rv 18 00050145
OV.161|orf.65183
cls.26.2.10|xxx|Beihai sobemo like virus 1|rv 18 00590238
OV.161|orf.8215
OV.161|orf.32086
cls.26.2.10|Solemoviridae|Turnip rosette virus|rv 18 01516280
cls.26.2.10|Solemoviridae|Artemisia virus A|rv 18 01537675
cls.26.2.10|Solemoviridae|Rottboellia yellow mottle virus|rv 18 00054942
cls.26.2.10|Solemoviridae|Cymbidium chlorotic mosaic virus|rv 18 00142689
cls.26.2.10|Solemoviridae|Papaya lethal yellowing virus|rv 18 01582164
cls.26.2.10|Solemoviridae|Sowbane mosaic virus|rv 18 01859257
cls.26.2.10|Solemoviridae|Soybean yellow common mosaic virus|rv 18 00287642
cls.26.2.10|Solemoviridae|Southern bean mosaic virus|rv 18 01818619
cls.26.2.10|Solemoviridae|Lucerne transient streak virus|rv 18 01516749
cls.26.2.10|Solemoviridae|Cocksfoot mottle virus Dactylis glomerata Norway CfMV NO 1995
cls.26.2.10|Solemoviridae|Rice yellow mottle virus|rv 18 00308809
cls.26.2.10|Solemoviridae|Velvet tobacco mottle virus|rv 18 01342407
cls.26.2.10|Solemoviridae|Solanum nodiflorum mottle virus|rv 18 00049334
Tree scale: 1
Dataset legend
RdRp
Capsid protein_Jelly-roll
Chymotrypsin-like protease
Capping_methyltransferase_guanylyltransferase
Superfamily 3 helicase
r84.0
Tombusvirus p33_Luteo_P1 domain_auxiliary replication protein
Superfamily 1 helicase
Major capsid protein_Toti-like (T=1)
Putative protease co-factor_calici-como32K-like
Maturation_Levi
Phospholipase A2
VP4_Dicistro
Capsid protein_Levi-like
PrsW-protease
small_ubiquitin-related_modifier_SUMO
Zinc-binding domain
Others

Ov201

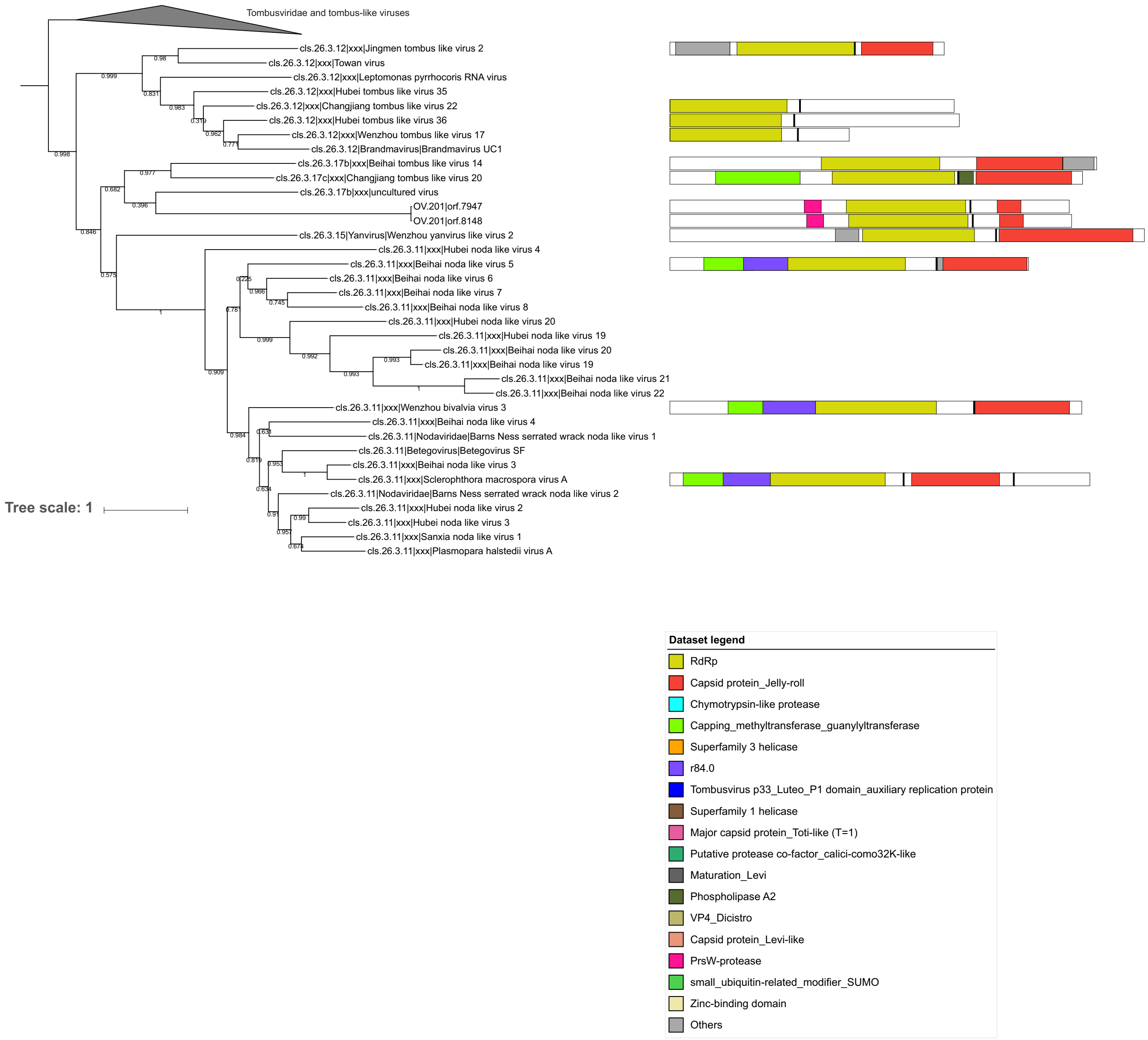
Tombusviridae and tombus-like viruses
cls.26.3.12|xxx|Jingmen tombus like virus 2
cls.26.3.12|xxx|Towan virus
cls.26.3.12|xxx|Leptomonas pyrrhocoris RNA virus
cls.26.3.12|xxx|Hubei tombus like virus 35
cls.26.3.12|xxx|Changjiang tombus like virus 22
cls.26.3.12|xxx|Hubei tombus like virus 36
cls.26.3.12|xxx|Wenzhou tombus like virus 17
cls.26.3.12|Brandmavirus|Brandmavirus UC1
cls.26.3.17b|xxx|Beihai tombus like virus 14
cls.26.3.17c|xxx|Changjiang tombus like virus 20
cls.26.3.17b|xxx|uncultured virus
OV.201|orf.7947
OV.201|orf.8148
cls.26.3.15|Yanvirus|Wenzhou yanvirus like virus 2
cls.26.3.11|xxx|Hubei noda like virus 4
cls.26.3.11|xxx|Beihai noda like virus 5
cls.26.3.11|xxx|Beihai noda like virus 6
cls.26.3.11|xxx|Beihai noda like virus 7
cls.26.3.11|xxx|Beihai noda like virus 8
cls.26.3.11|xxx|Hubei noda like virus 20
cls.26.3.11|xxx|Hubei noda like virus 19
cls.26.3.11|xxx|Beihai noda like virus 20
cls.26.3.11|xxx|Beihai noda like virus 19
cls.26.3.11|xxx|Beihai noda like virus 21
cls.26.3.11|xxx|Beihai noda like virus 22
cls.26.3.11|xxx|Wenzhou bivalvia virus 3
cls.26.3.11|xxx|Beihai noda like virus 4
cls.26.3.11|Nodaviridae|Barns Ness serrated wrack noda like virus 1
cls.26.3.11|Betegovirus|Betegovirus SF
cls.26.3.11|xxx|Beihai noda like virus 3
cls.26.3.11|xxx|Sclerophthora macrospora virus A
cls.26.3.11|Nodaviridae|Barns Ness serrated wrack noda like virus 2
cls.26.3.11|xxx|Hubei noda like virus 2
cls.26.3.11|xxx|Hubei noda like virus 3
cls.26.3.11|xxx|Sanxia noda like virus 1
cls.26.3.11|xxx|Plasmopara halstedii virus A
Tree scale: 1
Dataset legend
RdRp
Capsid protein_Jelly-roll
Chymotrypsin-like protease
Capping_methyltransferase_guanylyltransferase
Superfamily 3 helicase
r84.0
Tombusvirus p33_Luteo_P1 domain_auxiliary replication protein
Superfamily 1 helicase
Major capsid protein_Toti-like (T=1)
Putative protease co-factor_calici-como32K-like
Maturation_Levi
Phospholipase A2
VP4_Dicistro
Capsid protein_Levi-like
PrsW-protease
small_ubiquitin-related_modifier_SUMO
Zinc-binding domain
Others
